# Cross-tissue immune cell analysis reveals tissue-specific features in humans

**DOI:** 10.1126/science.abl5197

**Published:** 2022-05-13

**Authors:** C Domínguez Conde, C Xu, LB Jarvis, DB Rainbow, SB Wells, T Gomes, SK Howlett, O Suchanek, K Polanski, HW King, L Mamanova, N Huang, PA Szabo, L Richardson, L Bolt, ES Fasouli, KT Mahbubani, M Prete, L Tuck, N Richoz, ZK Tuong, L Campos, HS Mousa, EJ Needham, S Pritchard, T Li, R Elmentaite, J Park, E Rahmani, D Chen, DK Menon, OA Bayraktar, LK James, KB Meyer, N Yosef, MR Clatworthy, PA Sims, DL Farber, K Saeb-Parsy, JL Jones, SA Teichmann

**Affiliations:** 1 Wellcome Sanger Institute, Wellcome Genome Campus, Hinxton, Cambridge CB10 1SA, UK; 2 Department of Clinical Neurosciences, University of Cambridge; 3 Department of Systems Biology, Columbia University Irving Medical Center; 4 Molecular Immunity Unit, Department of Medicine, University of Cambridge, Cambridge, UK; 5 Centre for Immunobiology, Blizard Institute, Queen Mary University of London, London, UK; 6 Department of Microbiology and Immunology, Columbia University Irving Medical Center; 7 Department of Surgery, University of Cambridge and NIHR Cambridge Biomedical Research Centre, Cambridge, UK; 8 West Suffolk Hospital NHS Trust, Bury Saint Edmunds, UK; 9 Center for Computational Biology, University of California, Berkeley, Berkeley, CA, USA; 10 Department of Electrical Engineering and Computer Sciences, University of California, Berkeley, Berkeley, CA, USA; 11 Department of Anaesthesia, University of Cambridge, Cambridge, UK; 12 Chan Zuckerberg Biohub, San Francisco, CA, USA; 13 Ragon Institute of MGH, MIT and Harvard, Cambridge, MA, USA; 14 Theory of Condensed Matter, Cavendish Laboratory, Department of Physics, University of Cambridge, JJ Thomson Ave, Cambridge CB3 0HE, UK

## Abstract

Despite their crucial role in health and disease, our knowledge of immune cells within human tissues remains limited. Here, we surveyed the immune compartment of 16 tissues from 12 adult donors by single-cell RNA sequencing and VDJ sequencing generating a dataset of 360,000 cells. To systematically resolve immune cell heterogeneity across tissues, we developed CellTypist, a machine learning tool for rapid and precise cell type annotation. Using this approach, combined with detailed curation, we determined the tissue distribution of finely phenotyped immune cell types, revealing hitherto unappreciated tissue-specific features and clonal architecture of T and B cells. Our multi-tissue approach lays the foundation for identifying highly resolved immune cell types by leveraging a common reference dataset, tissue-integrated expression analysis and antigen receptor sequencing.

The immune system is a dynamic and integrated network made up of many different cell types distributed across the body that act together to maintain tissue homeostasis and mediate protective immunity. In recent years, a growing appreciation of immune ontogeny and diversity across tissues has emerged. For example, we have gained insights into how macrophages derived in embryogenesis contribute to the unique adaptation of tissue-resident myeloid cells, such as Langerhans cells in the skin, microglia in the brain and Kupffer cells in the liver (1–3). Other populations, such as innate lymphoid cells (ILCs), including natural killer (NK) cells and non-conventional (NKT, MAIT and γδ) T cells, have circulating counterparts but are highly enriched at barrier/mucosal sites where they mediate tissue defense and repair (4). In addition, following resolution of an immune response, antigen-specific, long-lived tissue-resident memory T cells (TRMs) persist in diverse sites, where they provide optimal protection against secondary infections (reviewed in (5–7)).

Studies in mice have revealed the central role of tissue immune responses in protective immunity, anti-tumor immunity, and tissue repair; however, human studies have largely focussed on peripheral blood as the most accessible site. Given that circulating immune cells comprise only a subset of the entire immune cell landscape, understanding human immunity requires a comprehensive assessment of the properties and features of immune cells within and across tissues. Recent progress in the analysis of tissue immune cells have implemented organ-focused approaches (8–12), while use of tissues obtained from organ donors allows for analysis of immune cells across multiple sites across an individual (13–19). We previously reported single-cell RNA sequencing (scRNA-seq) analysis of T cells in three tissues from two donors (20), identifying tissue-specific signatures. However, despite the effort to assemble murine (21) and human (22, 23) multi-tissue atlases, large-scale cross-tissue scRNA-seq studies that investigate tissue-specific features of innate and adaptive immune compartments have not been reported.

Furthermore, a fundamental challenge of increasingly large single cell transcriptomics data sets is cell type annotation, including identifying rare cell subsets and distinguishing novel discoveries from previously described cell populations. Currently available automated annotation workflows leverage organ-focussed studies and lack a comprehensive catalogue of all cell types found across tissues. Therefore, a single unified approach is required in order to provide an in-depth dissection of immune cell type and immune state heterogeneity across tissues.

To address these needs, we comprehensively profiled immune cell populations isolated from a wide range of donor-matched tissues from 12 deceased individuals, generating nearly 360,000 single cell transcriptomes. To systematically annotate multi-tissue immune cells we developed CellTypist, a machine learning framework for cell type prediction initially trained on data from studies across 20 human tissues (see Supplementary Text) and then updated and extended by integrating immune cells from our dataset.

## Results

### CellTypist: a pan-tissue immune database and a tool for automated cell type annotation

**Fig. 1:**
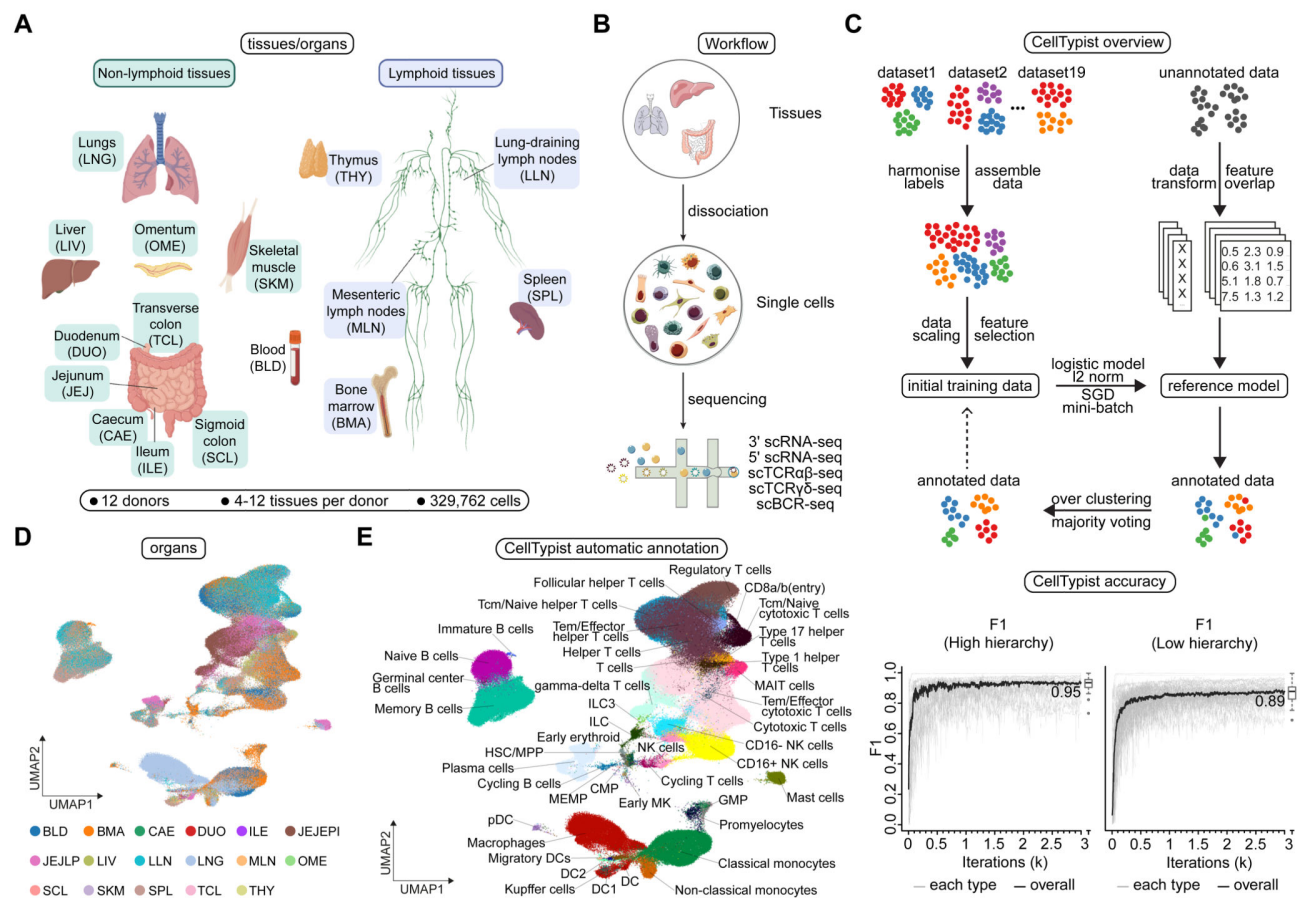
Automated annotation of immune cells across human tissues using CellTypist. (A) Schematic showing sample collections of human lymphoid and non-lymphoid tissues and their assigned tissue name acronyms. (B) Schematic of single-cell transcriptome profiling and paired sequencing of αβ TCR, γδ TCR and BCR variable regions. (C) Workflow of CellTypist including data collection, processing, model training and cell type prediction (upper panel). Performance curves showing the F1 score at each iteration of training with mini-batch stochastic gradient descent for high- and low-hierarchy CellTypist models, respectively (lower panel). The black curve represents the median F1 score averaged across the individual F1 scores of all predicted cell types. (D) UMAP visualization of the immune cell compartment colored by tissues. Note jejunum samples in (A) were further split into epithelial (JEJEPI) and lamina propria fractions (JEJLP). (E) As in (D), but colored by predicted cell types using CellTypist.

To systematically assess immune cell type heterogeneity across human tissues, we performed scRNA-seq on 16 different tissues from 12 deceased organ donors (Fig. 1, A and B, and table S1). The tissues studied included primary (bone marrow) and secondary (spleen, lung-draining and mesenteric lymph nodes) lymphoid organs, mucosal tissues (gut and lung), as well as blood and liver. When available, we also included additional donor-matched samples from tissues such as thymus, skeletal muscle and omentum. Immune cells were isolated from tissues as detailed in the Methods. After stringent quality control, we obtained a total of 357,211 high quality cells, of which 329,762 belonged to the immune compartment (fig. S1, A and B).

Robust cell type annotation remains a major challenge in single-cell transcriptomics. To computationally predict cellular heterogeneity in our dataset, we developed CellTypist (24), a cell type database, that in its current form is focused on immune cells, that provides a directly interpretable pipeline for the automatic annotation of scRNA-seq data (Fig. 1C). One of the unique and valuable aspects of CellTypist is that its training set encompasses a wide range of immune cell types across tissues. This is of critical importance given that immune compartments are shared across tissues, warranting accurate and automated cell annotation in an organ-agnostic manner. In brief, to develop CellTypist we integrated cells from 20 different tissues from 19 reference datasets (fig. S2) where we had deeply curated and harmonised cell types at two knowledge-driven levels of hierarchies (figs. S3 to S8). This was followed by a machine learning framework to train these cells using logistic regression with stochastic gradient descent learning (see methods). Performance of the derived models, as measured by the precision, recall and global F1-score, reached ~0.9 for cell type classification at both the high- and low-hierarchy levels (Fig. 1C and fig. S9, A and B). Notably, representation of a given cell type in the training data was a major determinant of its prediction accuracy (fig. S9C), implying higher model performance can be achieved by incorporating additional datasets. Moreover, CellTypist prediction was robust to differences between training and query datasets including gene expression sparseness (fig. S10) and batch effects (fig. S11).

First we applied CellTypist’s high-hierarchy (i.e. low-resolution, 32 cell types) classifier to our cross-tissue dataset (Fig. 1D), and found 15 major cell populations (fig. S1C). At this level of resolution, clear compositional patterns emerged in lymphoid *versus* non-lymphoid tissues, and within the lymphoid tissues between spleen *versus* lymph nodes, and appeared not to be driven by differences in dissociation protocols (fig. S12). As the training datasets of CellTypist contained hematopoietic tissues with definitive annotations for progenitor populations, the classifier was able to resolve several progenitors including hematopoietic stem cells and multipotent progenitors (HSC/MPP), promyelocytes, early megakaryocytes, pre-B and pro-B cells. Furthermore, CellTypist clearly resolved monocytes and macrophages, which often form a transcriptomic continuum in scRNA-seq datasets due to their functional plasticity. Thus CellTypist was successfully able to identify major groups of cell populations with different abundances in our dataset (fig. S1C).

To automatically annotate fine-grained immune sub-populations, we next applied the low-hierarchy (high-resolution, 91 cell types and subtypes) classifier, which was able to classify cells into 43 specific subtypes including subsets of T cells, B cells, ILCs, and mononuclear phagocytes (Fig. 1E). This revealed a high degree of heterogeneity within the T cell compartment, not only distinguishing between αβ and γδ T cells, but also between CD4+ and CD8+ T cell subtypes and their more detailed effector and functional phenotypes. Specifically, the CD4+ T cell cluster was classified into helper, regulatory and cytotoxic subsets, and the CD8+ T cell clusters contained unconventional T cell subpopulations such as MAIT cells. In the B cell compartment, a clear distinction was observed between naive and memory B cells. Moreover, CellTypist revealed three distinct subsets of dendritic cells (DC) - DC1, DC2 and migratory DCs (migDCs) (25, 26), again highlighting the granularity CellTypist can achieve. This fine-grained dissection of each compartment also allowed for the cross-validation and consolidation of cell types by examining the expression of marker genes derived from CellTypist models in cells from our dataset (fig. S1D).

In summary, we generated an in-depth map of immune cell populations across human tissues, and developed a framework for automated annotation of immune cell types and subtypes. CellTypist produced fine-grained annotations on our multi-tissue and multi-lineage dataset, and its performance, as assessed on multiple metrics, was comparable or better relative to other label-transfer methods with minimal computational cost (figs. S13 and S14). This approach allowed us to refine the description of many cell subtypes such as the progenitors and dendritic cell compartments at full transcriptomic breadth, resolving 43 cell states in total across our dataset. This automated annotation forms the basis for further cross-tissue comparisons of cell compartments in the sections below.

### Tissue-restricted features of mononuclear phagocytes

**Fig. 2:**
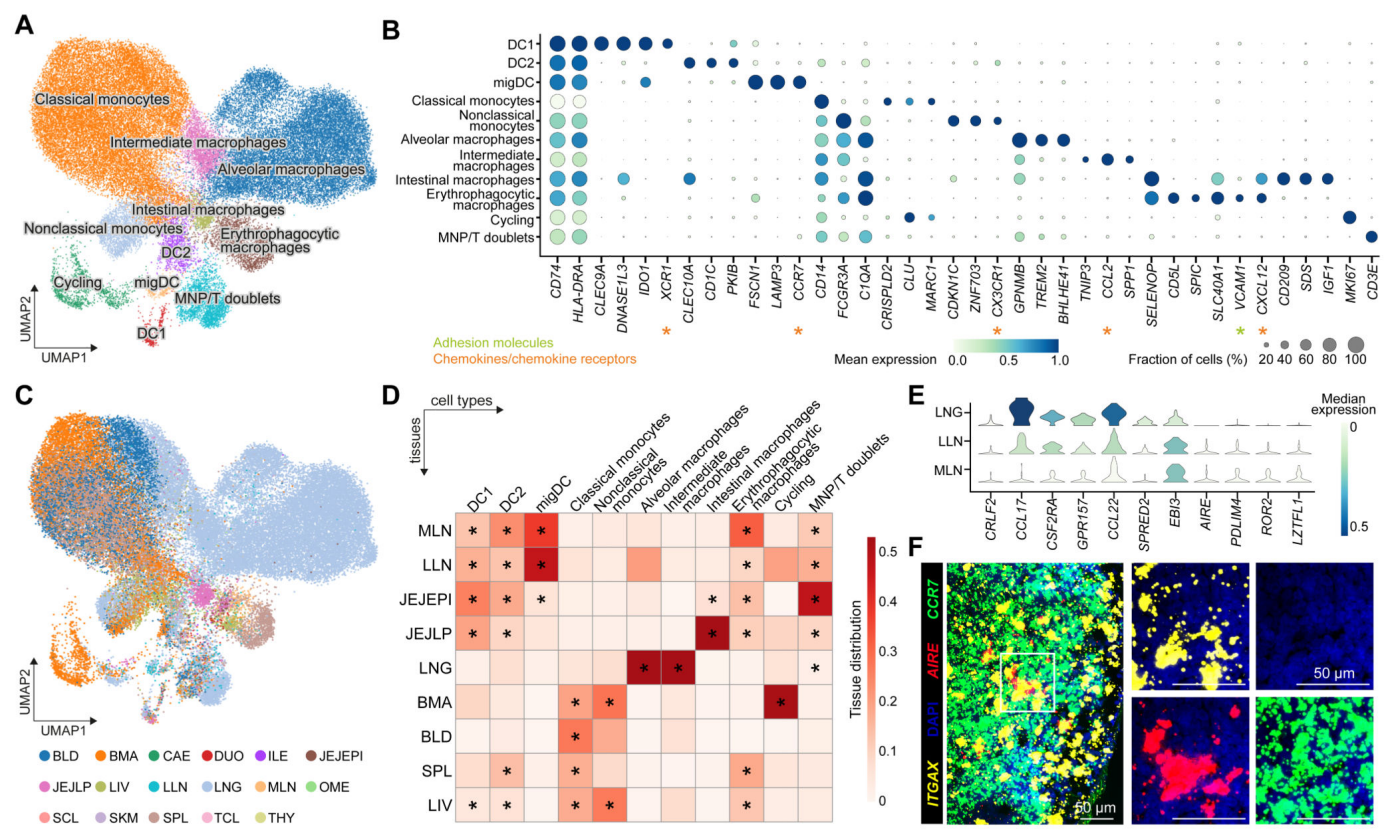
Myeloid compartment across tissues. (A) UMAP visualization of the cell populations in the myeloid compartment. (B) Dot plot for expression of marker genes of the identified myeloid populations. Color represents maximum-normalized mean expression of cells expressing marker genes, and size represents the percentage of cells expressing these genes. (C) UMAP visualization of the tissue distribution in the myeloid compartment. (D) Heatmap showing the distribution of each myeloid cell population across different tissues. Cell numbers are normalized within each tissue and later calculated as proportions across tissues. Only tissues containing more than 50 myeloid cells in at least two donors were included. Asterisks mark significant enrichment in a given tissue relative to the remaining tissues (poisson regression stratified by donors, *p* < 0.05 after Benjamini-Hochberg (BH) correction). (E) Violin plot for genes differentially expressed in migratory dendritic cells across tissues. Color represents maximum-normalized mean expression of cells expressing marker genes. (F) smFISH visualisation of *ITGAX, CCR7* and *AIRE* transcripts, validating the *AIRE+* migratory dendritic cells in lung-draining lymph nodes.

Mononuclear phagocytes, including monocytes, macrophages and dendritic cells, are critical for immune surveillance and tissue homeostasis. Automatic annotation by CellTypist identified eight populations in this compartment (fig. S15A). To explore macrophage heterogeneity further, we built on CellTypist’s annotation by performing additional manual curation, which revealed further heterogeneity within the macrophages (Fig. 2A and fig. S15B). The identities of these cells were supported by expression of well-established marker genes (Fig. 2B), and by markers independently identified from CellTypist models (fig. S15C). Moreover, existence of these cell types was cross-validated, and thus consolidated, in the training datasets of CellTypist (fig. S15D), as well as in myeloid cells from two additional studies of the human intestinal tract (27) (fig. S15E) and lung (28) (fig. S15F).

Among macrophages, lung-resident cells constituted the majority, and were classified into two major clusters: (i) alveolar macrophages expressing *GPNMB* and *TREM2*, markers that have been related to alveolar macrophages (29) and disease-associated macrophages (30), respectively; and (ii) intermediate macrophages with unique expression of *TNIP3* (Fig. 2, B to D). TNIP3 (TNFAIP3-interacting protein 3) binds to A20, encoded by *TNFAIP3*, and inhibits TNF, IL-1 and LPS-induced NF-kB activation (31). Its expression in lung macrophages may be related to underlying pathology as it was primarily detected in one donor (A29), a multitrauma donor with significant lung contusions. Notably, this population also expressed the monocyte-recruiting chemokine CCL2 (Fig. 2B), providing a means of replenishing the lung macrophage pool.

Other macrophage subsets in our data also showed a high degree of tissue restriction (Fig. 2D). Erythrophagocytic macrophages, including red pulp macrophages and Kupffer cells, mainly populated the spleen and liver, as expected, and shared high expression of *CD5L, SCL40A1* and the transcription factor *SPIC* (32). Notably, a number of macrophages from lymph nodes clustered together with erythrophagocytic macrophages, pointing to the presence of a small number of iron-recycling macrophages in lymph nodes (Fig. 2D). Another macrophage population specifically present in the gut expressed *CD209* (encoding DC-SIGN) and *IGF1*, markers that have been previously reported in mature intestinal macrophages and M2-like macrophages, respectively (33, 34). Lastly, monocytes were clearly grouped in two major subgroups, classical and non-classical monocytes, which differed in the expression of *CD14, FCGR3A* and *CX3CR1* as well as in their tissue distribution (Fig. 2, A to D).

Among dendritic cells, DC1 expressed *XCR1* and *CLEC9A*, consistent with their identity as conventional DCs (DC1), specialised for cross-presentation of antigens (Fig. 2B). Conventional DC2s expressed *CD1C* and *CLEC10A*, and migDCs were *CCR7*+ *LAMP3*+. *CCR7* is upregulated in tissue DCs following TLR or FcγR ligation (35, 36), enabling migration towards CCL19/21-expressing lymphatic endothelium and stromal cells in the T cell zone of lymph nodes (37, 38). Consistent with this, we observed a marked enrichment of CCR7+ migDCs in lung-draining and mesenteric lymph nodes (Fig. 2D). Interestingly, migDCs showed upregulation of *AIRE, PDLIM4* and *EBI3* in lymph nodes (Fig. 2E). Extra-thymic expression of the autoimmune regulator AIRE has been reported in humans (39, 40), however, its functional role in secondary lymphoid organs remains poorly understood and is a matter of intense research (41–43). We validated the presence of migDCs in lung-draining lymph nodes by immunofluorescence (fig. S16A) and *AIRE* expression by single-molecule FISH (smFISH) (Fig. 2F). In addition, another subpopulation of migDCs found in lung and lung-draining lymph nodes upregulated *CRLF2* (encoding TLSPR), chemokines (*CCL22, CCL17*), *CSF2RA* and *GPR157* (Fig. 2E). TLSPR is involved in the induction of Th2 responses in asthma (44). Expression of these genes in lung DCs was also observed in published scRNA-seq datasets (45, 46) (fig. S16, B and C). These observations suggest that dendritic cell activation coincides with the acquisition of tissue-specific markers that differ depending on the local microenvironment.

Overall, our analysis of the myeloid compartment has revealed shared and tissue-restricted features of mononuclear phagocytes, including alveolar macrophages in the lung, iron-recycling macrophages mostly localized in the spleen, and subtypes of migratory dendritic cells.

### B cell subsets and immunoglobulin repertoires across tissues

**Fig. 3:**
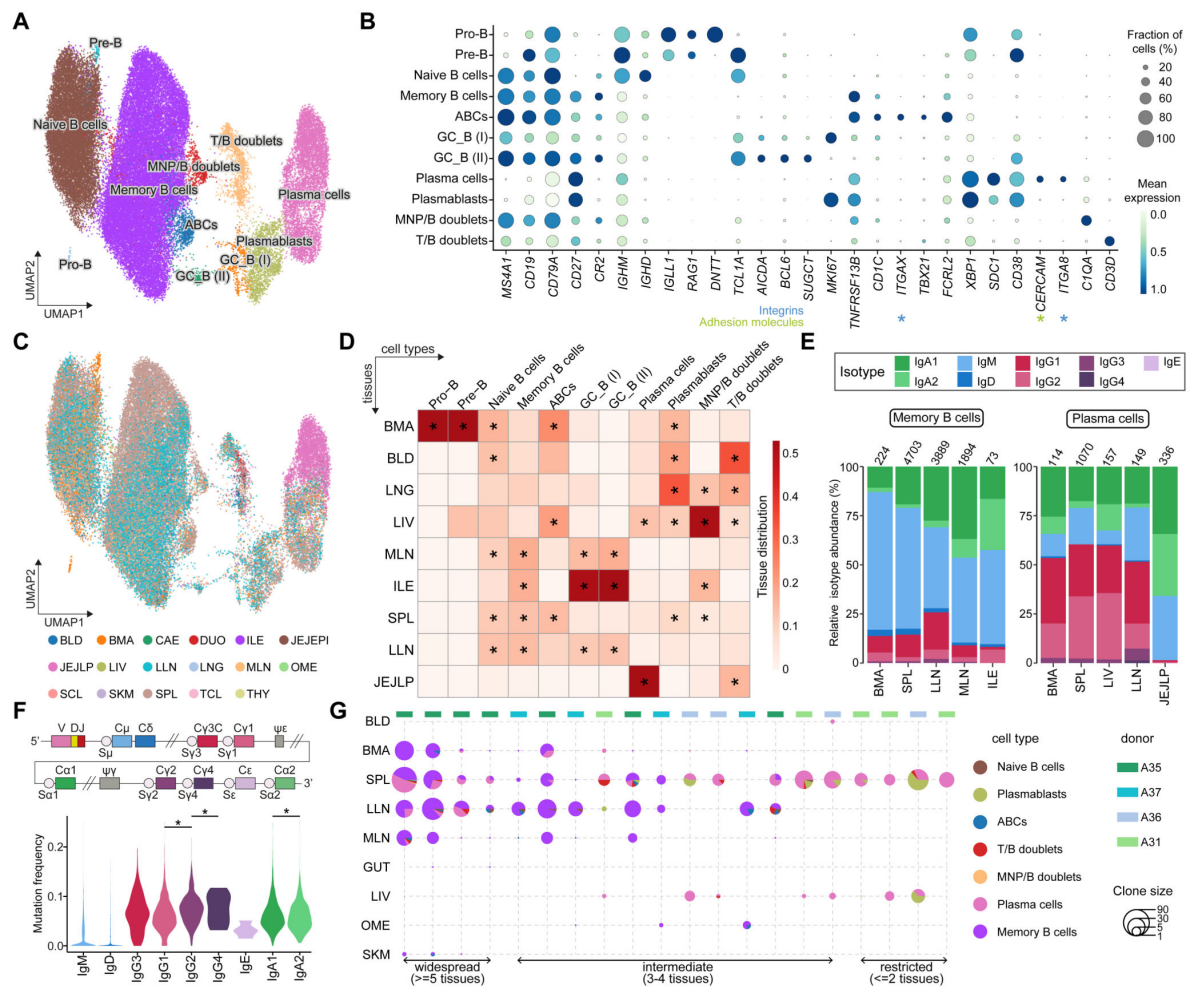
B cell compartment across tissues. (A) UMAP visualization of the cell populations in the B cell compartment. (B) Dot plot for expression of marker genes of the identified B cell populations. Color represents maximum-normalized mean expression of cells expressing marker genes, and size represents the percentage of cells expressing these genes. (C) UMAP visualization of the tissue distribution in the B cell compartment. (D) Heatmap showing the distribution of each B cell population across different tissues. Cell numbers are normalised within each tissue and later calculated as proportions across tissues. Only tissues containing more than 50 B cells in at least two donors were included. Asterisks mark significant enrichment in a given tissue relative to the remaining tissues (poisson regression stratified by donors, *p* < 0.05 after Benjamini-Hochberg (BH) correction). (E) Stacked bar plots showing the isotype distribution per tissue within memory B cells and the plasma cells. (F) Violin plot of the hypermutation frequency on the IgH chain across isotypes. Significant difference among IgG4, IgG2 and IgG1, as well as between IgA2 and IgA1 is marked by asterisks (wilcoxon rank sum test, *p* < 0.05). (G) Scatterpie plot showing the tissue distribution and B cell subsets of expanded clonotypes (>10 cells). Each vertical line represents one clonotype.

B cells comprise progenitors in the bone marrow, developmental states in lymphoid tissues and terminally differentiated memory and plasma cell states in lymphoid and non-lymphoid tissues. They play a central role in humoral immunity *via* the production of antibodies tailored to specific body sites. We first annotated the B cells using CellTypist and obtained six populations (fig. S17A). Manual curation revealed further heterogeneity in memory B cells and plasma cells, identifying 11 cell types in total (Fig. 3A and fig. S17B). As with the myeloid compartment, we cross-checked and verified these cell types in CellTypist training datasets (fig. S17, C and D) and in two independent immune datasets from gut (27) and lung (28) (fig. S17, E and F).

Naive B cells expressed *TCL1A* and were primarily found in lymphoid tissues (Fig. 3, B to D). In addition, we identified two populations of germinal center B cells, expressing *AICDA* and *BCL6*, that differed in their proliferative states (marked by *MKI67*). Of note, we did not find differential expression of dark zone and light zone marker genes in these two populations, probably reflecting limited germinal center activity in our adult donors. Moreover, these germinal center populations were present in lymph nodes and diverse gut regions (Fig. 3, C and D), presumably representing Peyer’s patches. Within memory B cells, which were characterized by expression of the B-cell lineage markers (*MS4A1, CD19*) and *TNFRSF13B*, we found a transcriptomically distinct cluster positive for *ITGAX, TBX21* and *FCRL2*, encoding CD11c, T-bet and the Fc receptor-like protein 2, respectively (Fig. 3B). CD11c+T-bet+ B cells, also known as age-associated B cells (ABCs), have been reported in autoimmunity and aging (47–49), and likely correspond to this *ITGAX+* memory B cell population. Notably, unlike conventional memory B cells, they showed relatively low expression of *CR2* (encoding CD21) and *CD27* (Fig. 3B). Interestingly, this subset was mainly present in the liver and bone marrow, while in secondary lymphoid organs, it was primarily found in the spleen (confirmed by flow cytometry and immunofluorescence (Fig. 3, C and D, and fig. S18). This data deepens our understanding of the phenotype of this non-classical subtype of memory B cells, and their tissue distribution.

We uncovered two populations of plasmablasts and plasma cells marked by expression of *CD38, XBP1* and *SDC1*. Whereas the former expressed *MKI67* and were found in the spleen, liver, bone marrow and blood, the latter expressed the integrin alpha-8-encoding gene *ITGA8* and the adhesion molecule *CERCAM* and were enriched in the jejunum and liver (Fig. 3, B to D). *ITGA8+* plasma cells have recently been reported in the context of an analysis of bone marrow plasma cells (50), and are likely a long-lived plasma cell population that is quiescent and tissue-resident. Here we expand their tissue distribution to the liver and gut, and describe their specific clonal distribution pattern below.

B cells have an additional source of variability due to VDJ recombination, somatic hypermutation and class-switching, which can relate to cell phenotype. Therefore, we performed targeted enrichment and sequencing of B-cell receptor (BCR) transcripts to assess isotypes, hypermutation levels and clonal architecture of the B cell populations identified above. This analysis revealed an isotype and subclass usage pattern that related to cellular phenotype (fig. S19A). As expected, naive B cells mainly showed a subclass preference for IgM and IgD. Interestingly, while evidence of class switching to IgA1 and IgG1 was seen within memory B cells (including ABCs), plasmablasts and plasma cells also showed class switching to IgA2 and IgG2.

To determine to what extent this isotype subclass bias correlated with the tissue of origin, we assessed each cell state independently (requiring a minimum cell count of 50). Memory B cells showed a bias towards IgA1 in the mesenteric lymph nodes, and towards IgA2 in the ileum, where Peyer’s patches are found (Fig. 3E). In the plasma cell compartment, we found an even more striking preference for IgA2 in the gut region (specifically in the jejunum lamina propria), consistent with the known dominance of this isotype at mucosal surfaces (Fig. 3E). Of note, plasma cells in the bone marrow, liver and spleen were composed of over 20% IgG2 subclass. With more limited numbers, we report isotype distributions across tissues for other B cell populations (fig. S19, B and C). ABCs were dominated by IgM in both the spleen and lung-draining lymph nodes, consistent with previous reports (51).

Somatic hypermutation (SHM) levels were, as expected, lowest in naive B cells and highest in plasma cells (fig. S19D). Between isotypes and subclasses, SHM did not differ significantly. Nonetheless, there was a tendency towards higher mutation rates in the distal classes IgG2, IgG4 and IgA2, which are downstream from the IgH locus and can thus accumulate more mutations during sequential switching (52) (Fig. 3F). We also explored the occurrence of sequential class switching events in our data by assessing the isotype frequency among expanded clonotypes (>10 cells). Mixed isotype clones were rare in our data (fig. S19E).

Next, we evaluated the distribution of expanded clones across tissues and cell types, and found three major groups of clones - present in only two tissues, three to four tissues or five or more tissues, respectively (Fig. 3G), similar to previously reported patterns of B cell clonal tissue distribution (53). While the clones restricted to two tissues, typically between the spleen and the liver or bone marrow, were enriched in plasma cells, those distributed across more than five tissues, including lymph nodes, were over-represented in memory B cells. Together, these findings suggest that tissue-restricted clones may represent long-term immunological memory maintained by long-lived plasma cells resident in the bone marrow and spleen as well as liver in our data.

Overall, we characterized nine cell states in the B cell compartment, and gained insights from in-depth characterisation of both naive and memory B cell as well as plasma cell subsets. We identified distinct clonal distribution patterns for the more tissue-restricted long-lived quiescent plasma cells *versus* the broad tissue distribution of classical memory B cell clones.

### scRNA-seq analysis of T cells and innate lymphocytes reveals lineage and tissue-specific subsets

**Fig. 4:**
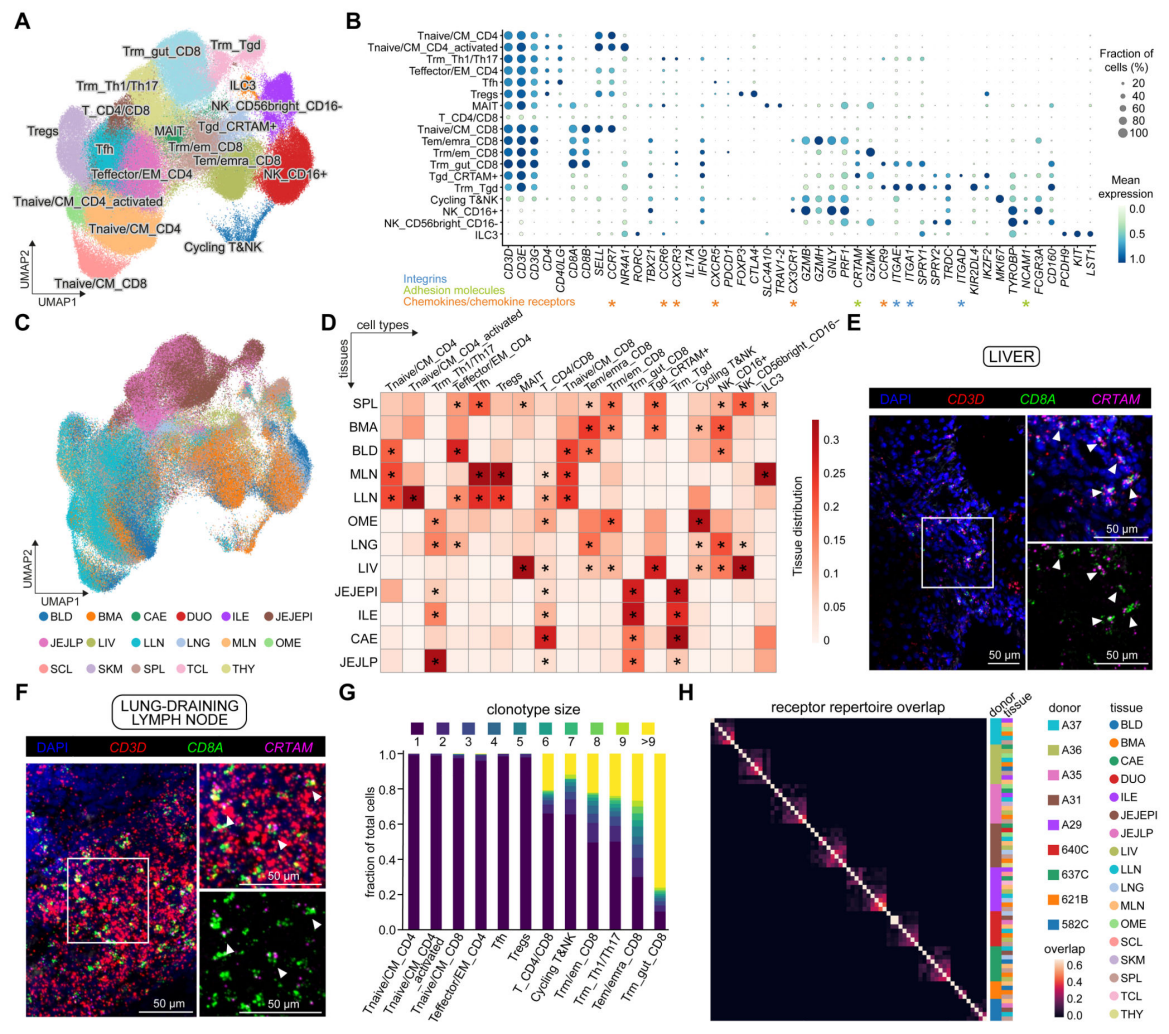
Tissue compartmentalization and site-specific adaptations of T cells and innate lymphoid cells (ILCs). (A) UMAP visualization of T cells and ILCs across human tissues colored by cell types. (B) Dot plot for expression of marker genes of the identified immune populations. Color represents maximum-normalized mean expression of cells expressing marker genes, and size represents the percentage of cells expressing these genes. (C) UMAP visualization of T cells and ILCs colored by tissues. (D) Heatmap showing the distribution of each T cell or ILC population across different tissues. Cell numbers are normalized within each tissue and later calculated as proportions across tissues. Only tissues containing more than 50 ILC/T cells in at least two donors were included. Asterisks mark significant enrichment in a given tissue relative to the remaining tissues (Poisson regression stratified by donors, *p* < 0.05 after Benjamini-Hochberg (BH) correction). (E and F) smFISH visualisation of *CD3D, CD8A* and *CRTAM* transcripts, validating the tissue-resident memory CD8+ T cell population in the liver and lung-draining lymph nodes. (G) TCR repertoire analysis of T cells across tissues. Stacked bar plot shows the fraction of cells in a given cluster binned by clonotype size. (H) Heatmap showing the repertoire overlap between expanded clones (>1 cell) across tissues and donors as determined by jaccard distance.

For annotation of the T cell/innate lymphocyte compartment, CellTypist identified 18 cell types (fig. S20A). After manual inspection, these clusters were further divided into additional subtypes (e.g. for cytotoxic T cells) (Fig. 4A and fig. S20B). As described above for the myeloid and B cell compartments, identities of the derived cell types were cross-validated in the immune compartment of the CellTypist training datasets (fig. S20, C and D) and the two independent studies of gut (27) and lung (28) (fig. S20, E and F).

Naive/central memory CD4+ T cells were transcriptionally close to naive CD8+ T cells as defined by high expression of *CCR7* and *SELL* and were mainly found in lymphoid sites (Fig. 4B). Other CD4+ T cells identified included follicular helper T cells (Tfh) expressing *CXCR5*, regulatory T cells (Tregs) expressing *FOXP3* and *CTLA4*, effector memory CD4+ T cells, and tissue-resident memory Th1 and Th17 cells expressing *CCR9, ITGAE* and *ITGA1* found largely in intestinal sites (jejunum and ileum) and lungs (Fig. 4, B to D). Within the memory CD8+ T compartment, we found three major subsets: Trm_gut_CD8 (resident memory T cells, Trm), Tem/emra_CD8 (effector memory, Tem; effector memory re-expressing CD45RA, Temra) and Trm/em_CD8. These subsets were characterized by differential expression of the chemokine receptors *CCR9* and *CX3CR1* and the activation marker *CRTAM* (Fig. 4B). The Trm_gut_CD8 population (CCR9+) expressed the tissue-residency markers *ITGAE* and *ITGA1*, encoding CD103 and CD49a respectively and localized to intestinal sites (Fig. 4B). By contrast, the Tem/Temra_CD8 population expressing *CX3CR1* was found in blood-rich sites (blood, bone marrow, lung, and liver) and was excluded from lymph nodes and gut (Fig. 4, C and D), consistent with previous flow cytometry analysis of Temra cells (54), and results showing CX3CR1+CD8+ T cells as blood-confined and absent from thoracic duct lymph (55). The Trm/em_CD8 population expressed high levels of *CRTAM*, a gene previously shown to be expressed by Trm (56) and was found in spleen, bone marrow, and to a lesser extent in lymph nodes and lungs. This may be a resident population more prevalent in lymphoid sites (16). We validated and mapped the Trm/em_CD8 population using smFISH in the liver (Fig. 4E) and lung-draining lymph nodes (Fig. 4F). Furthermore, we validated all three memory CD8+ T cell populations at the protein level by flow cytometry of cells purified from human spleen (fig. S21). Although we could validate *CRTAM* at the RNA level by smFISH, the protein could not be detected without stimulation, suggesting that CRTAM is subject to post-translational regulation upon T-cell receptor (TCR) activation. These three distinct populations represent different states of tissue adaptation and maturation between effector memory and tissue-resident T cell memory states.

We also detected invariant T cell subsets such as MAIT cells, characterised by expression of *TRAV1-2* and *SLC4A10*, and two populations of γδ T cells: Trm_Tgd and Tgd_CRTAM+. The *CCR9+* Trm_Tgd population populated the gut and expressed the tissue-residency markers *ITGAE* and *ITGA1*, whereas the Tgd_CRTAM+ population overexpressed *CRTAM, IKZF2* (encoding HELIOS) and the integrin molecule *ITGAD* (encoding CD11d) and was found primarily in the spleen, bone marrow and liver (Fig. 4, B to D, and fig. S22, A and B). We validated the latter population by quantitative PCR (qPCR) of flow sorted CD3+TCRγδ+ and CD3+TCRαβ+ cells from cryopreserved spleen samples from three donors (fig. S22C, D). As a small fraction of ɑβ T cells, marked by low expression of CD52 and CD127, were also noted to express *ITGAD*, the CD3+TCRαβ population was split into CD52-CD127- and CD52+CD127+ subpopulations. In keeping with our scRNA-seq data, *ITGAD* expression was high in CD3+TCRγδ and CD52-CD127-CD3+TCRαβ, providing additional evidence for the specific expression of this integrin alpha subunit in this subpopulation of γδ T cells.

Lastly, NK cells in our data were represented by two clusters with high expression of either *FCGR3A* (encoding CD16) or *NCAM1* (encoding CD56). We also defined an ILC3 population within a small cluster mixed with NK cells, via expression of markers including *PCDH9* (Fig. 4, A and B). Analyses of the tissue distribution of these populations revealed that, whereas the majority of CD4+ T and ILC3 cells were located in the lymph nodes and to some extent in the spleen, cytotoxic T and NK cells were more abundant in the bone marrow, spleen and non-lymphoid tissues (Fig. 4, C and D).

### TCR repertoire analysis shows clonal expansion and distribution patterns within and across tissues

To understand T cell-mediated protection in more depth, we analysed T cell clonal distribution in a subset of the data within different tissues of a single individual and across different individuals. Chain pairing analysis showed that cells from the T cell clusters mostly contained a single pair of chains (50-60%), with orphan (5-20%) and extra (5-10%) chains present in small fractions of cells (fig. S23A). Notably, the frequency of extra α chains (extra VJ) was higher than that of β chains (extra VDJ), potentially due to more stringent and multi-layered allelic exclusion mechanisms at the TCRβ locus compared to TCRα (57). As expected, the NK and ILC clusters held no productive TCR chains. Within the γδ T cell clusters, only a small proportion had a productive TCR chain, which may result from cytotoxic T cells co-clustering with γδ T cells. We also carried out γδ TCR sequencing in selected spleen, bone marrow and liver samples. The γδ TCR sequencing data was subjected to a customized analysis pipeline that we developed and optimised based on cellranger followed by contig re-annotation with dandelion (see Materials and Methods), facilitating the full recovery of γδ chains in our data. This analysis confirmed that the majority of productive γδ TCR chains originated from the *ITGAD*-expressing γδ T cells (fig. S23B), supporting the robust identification of this population. The Trm_Tgd population could not be confirmed by γδ TCR sequencing due to the lack of sample availability.

We next examined V(D)J gene usage in relation to T cell identity. In the MAIT population, we detected significant enrichment of *TRAV1-2*, as expected (fig. S23C). Selecting only the *TRAV1-2+* cells (MAIT cluster and other clusters) revealed a notable tissue-specific distribution of *TRAJ* segments with *TRAJ33* in spleen and liver, *TRAJ12* in liver and *TRAJ29/TRAJ36* in jejunum (fig. S23D). This suggests that there may be different antigens for MAIT cells in the spleen, liver and gut corresponding to the different metabolomes in these tissues. In addition, full analysis of the TCR repertoire of the MAIT cells revealed previously unappreciated diversity of V segment usage in the beta chain (fig. S23D).

We then defined clonally related cells on the basis of identical CDR3 nucleotide sequences to investigate their TCR repertoires. Using this approach, we found that clonally expanded cells were primarily from the resident memory T cell compartment, including the Th1/Th17 populations mentioned above (Fig. 4G and fig. S23E). As expected, these clonotypes were restricted to single individuals and within an individual they were distributed across tissues and subsets (Fig. 4H and fig. S23, F to H). We found a small number of isolated CD4+ T cell clones that shared Tregs and effector T cell phenotypes, possibly due to low levels of plasticity or due to (trans)differentiation from the same naive precursor cell in the periphery (fig. S23H). Focusing on the most expanded clonotypes (>20 cells), the majority were widespread across five or more tissues, supporting the systemic nature of tissue-resident immune memory (Fig. 4G). Moreover, we found that several clonotypes present across tissues consisted of a mixture of cells from the Tem/emra_CD8 and Trm/em_CD8 populations (fig. S23H), suggesting that a single naive CD8+ T cell precursor can give rise to diverse cytotoxic T cell states, which harbour immune memory across multiple non-lymphoid tissues, emphasizing the plasticity of phenotype and location within a clone.

In summary, we have described 18 T/innate cell states in our data by integrating CellTypist logistic regression models, manual curation and V(D)J sequencing. This has yielded insights into the MAIT cell compartment and its antigen receptor repertoire distribution that differed between spleen, liver and gut. For the cytotoxic T cell memory compartment there was broad sharing of clones across gut regions for Trm_gut_CD8, and mixed Tem/emra_CD8 and Trm/em_CD8 T cell clonotypes with broad tissue distributions.

### A cross-tissue updatable reference of immune cell types and states

**Fig. 5:**
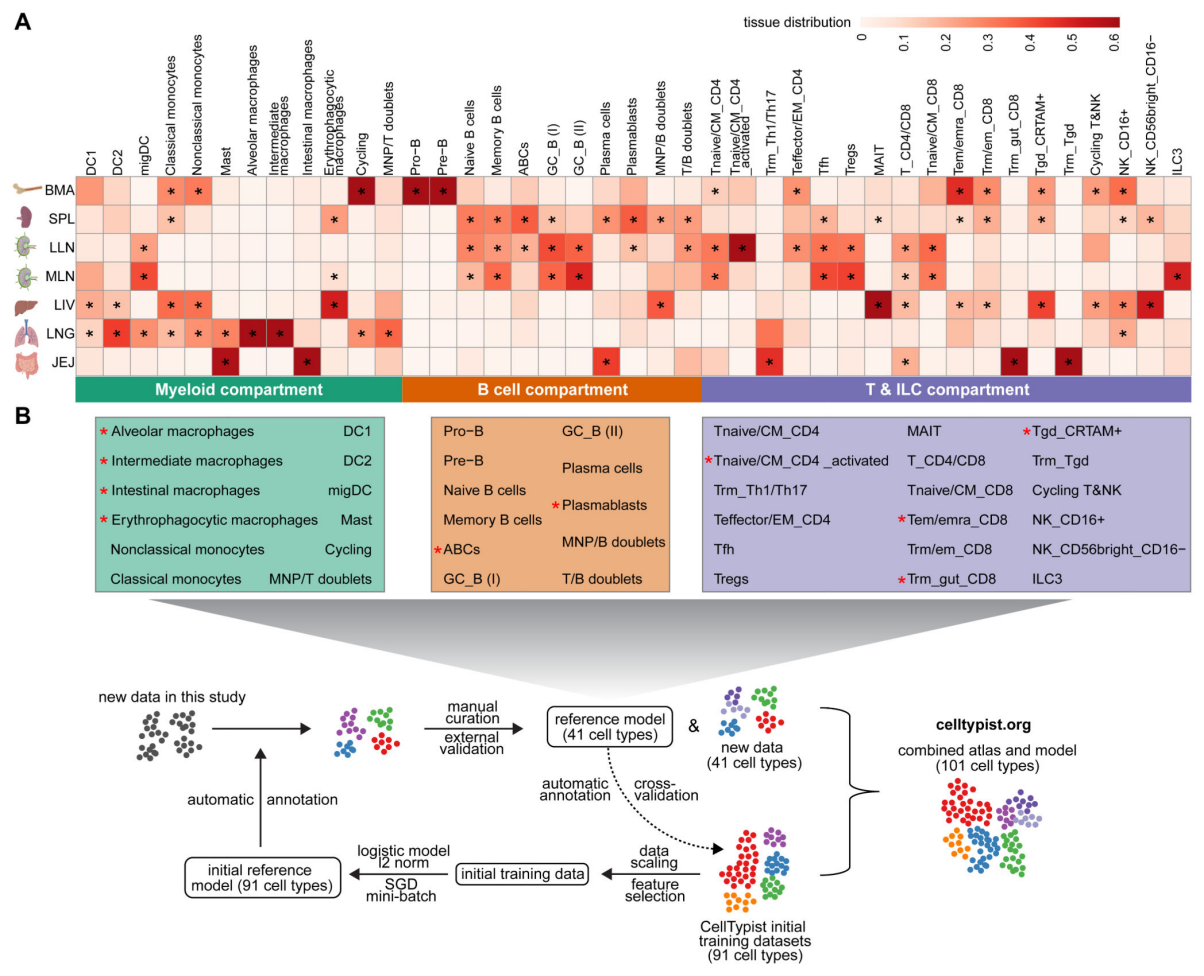
A cross-tissue updatable reference of immune cell types and cell states. (A) Heatmap showing the distribution of manually curated cell types across selected tissues. Cell numbers are normalized within each tissue and later calculated as proportions across tissues. Asterisks mark significant enrichment in a given tissue relative to the remaining tissues (Poisson regression stratified by donors, *p* < 0.05 after Benjamini-Hochberg (BH) correction). (B) Workflow for the iterative update of CellTypist through the periodic incorporation of curated cell type labels.

After focusing on individual immune compartments, we next took a combined approach in order to better understand the immune landscape of selected tissues. As shown in Fig. 5A, each tissue has its own immune neighborhood, for example, while spleen and lymph nodes are rich in B cells, composition of their myeloid compartment varies. In particular, a large population of erythrophagocytic macrophages, known as red pulp macrophages, are evident in the spleen (in keeping with their role in red blood cell turnover), whereas lymph nodes are rich in dendritic cells. As expected, bone marrow uniquely contains progenitor populations. Furthermore lung and liver contain significant numbers of monocytes, including *CX3CR1+* nonclassical monocytes whereas these cells are absent from the jejunum, perhaps reflecting different degrees of vascularization. In contrast, the jejunum has an abundance of resident memory T cells (CD8+ T cells and Th1/Th17) as well as plasma cells.

Our long-term vision for CellTypist is to provide a reference atlas with deeply curated cell types publicly available to the community. Therefore, via a semi-automatic process, we fed the identities of the 41 immune cell types identified in our dataset (including both shared and novel cell type labels) back into CellTypist, demonstrating how CellTypist can be updated and improved over time. Combined with the initial 91 cell types and states included in the reference datasets, CellTypist now comprises a total of 101 annotated cell types (Fig. 5B).

## Discussion

Here, we present a multi-tissue study of immune cells across the human body within diverse organ donors. By sampling multiple organs from the same individuals, which allows for robust control of age, sex, medical history, drug exposure and sampling backgrounds, we reveal tissue-specific expression patterns across the myeloid and lymphoid compartments.

We also introduce CellTypist, a publicly available and updatable framework for automated immune cell type annotation that, in addition to identifying major cell types, is able to perform fine-grained cell subtype annotation - normally a time-consuming process that requires expert knowledge. We developed CellTypist by integrating and curating data obtained from 19 studies performed across a range of tissues, with in-depth immune cell analysis comprising 91 harmonized cell type labels. However, as demonstrated here, for example in the γδ T cell compartment, manual curation following automated annotation still has a role to play in revealing specific cell subtypes that may be absent from the database/training set. To reduce the need for this, in the longer term, the CellTypist models will be periodically updated and extended to include further immune and non-immune sub-populations as more data become available.

Within the myeloid compartment, macrophages showed the most prominent features of tissue specificity. Erythrophagocytic macrophages in the liver and spleen shared features related to iron-recycling (58) with macrophages in other locations, such as the mesenteric lymph nodes, suggesting that macrophages participate in iron metabolism across a range of tissues. In addition, we characterized subsets of migratory dendritic cells (CCR7+) revealing specific expression of *CRLF2, CSF2RA* and *GPR157* in the lung and lung-draining lymph nodes, and expression of *AIRE* in the mesenteric and lung-draining lymph nodes. These migratory dendritic cell states are interesting targets for future in depth functional characterisation in the context of allergy, asthma and other related pathologies (59, 60).

In the lymphoid compartment, we combined single-cell transcriptome and VDJ analysis, which allowed the phenotype of adaptive immune cells to be dissected using complementary layers of single cell genomics data. Of note, we detected a subset of memory B cells expressing *ITGAX* (CD11c) and *TBX21* (T-bet) that resemble ABCs previously reported to be expanded in ageing (48), following malaria vaccination (61) and in systemic lupus erythematosus (SLE) patients (62). In our data, these B cells did not show clonal expansion and at least 50% showed IgM subclass, suggesting that they may be present at low levels in healthy tissues and expand upon challenge as well as ageing. BCR analysis revealed isotype usage biased towards IgA2 in gut plasma cells, which may be related to structural differences (63) or higher resistance to microbial degradation as compared to IgA1 (64).

In the T cell compartment, our results provided insights into the heterogeneity of T cell subtypes and their tissue adaptations. Notably, we identified subsets of CD4+ Trm based on functional capacity for IFN-γ or IL-17 production that were mostly localized to intestinal sites, analogous to mouse CD4+ Trm generated from IL-17-producing effector T cells in the gut (65). We also identified different subsets of CD8+ Trm including a gut-adapted subset expressing *CCR9*, which mediates homing to intestinal sites via binding to CCL25 (66) and another Trm-like subset more targeted to lymphoid sites. TCR clone sharing between memory subtypes of CD8+ T cells suggests their origin from a common precursor, or their differentiation or conversion during migration or maintenance, such as conversion of effector memory T cells (Tem) to resident memory T cells (Trm) (56). We also identified distinct subsets of γδ T cells based on tissue-specific gene expression patterns, showing distinct integrin gene expression and tissue distributions.

In summary, using this dataset of nearly 360,000 single cell transcriptomes (of which ~330,000 were immune cells) from donor-matched tissues from 12 deceased individuals, we have shown how a combination of CellTypist-based automated annotation, expert-driven cluster analysis and antigen receptor sequencing can synergize to dissect specific and functionally relevant aspects of immune cells across the human body. We have revealed previously unrecognized features of tissue-specific immunity in the myeloid and lymphoid compartments, and have provided a comprehensive framework for future cross-tissue cell type analysis. Further investigation of human tissue-resident immunity is needed to determine the effect of important covariates such as underlying critical illness, donor age and gender as well as considering the immune cell activation status, to gain a defining picture of how human biology influences immune functions. Our deeply characterised cross-tissue immune cell dataset has implications for the engineering of cells for therapeutic purposes and addressing cells to intended tissue locations, and for understanding tissue-specific features of infection as well as distinct modes of vaccine delivery to tissues.

## Materials and methods

### Tissue acquisition, processing and single-cell sequencing

Tissue was obtained from deceased organ donors via the Cambridge Biorepository for Translational Medicine (CBTM, https://www.cbtm.group.cam.ac.uk/), REC 15/EE/0152. Detailed sample locations taken can be found in Fig. 1 and protocols are described in detail in Supplementary Materials. Additional tissue samples were from Columbia University and were obtained from deceased organ donors at the time of organ acquisition for clinical transplantation through an approved protocol and material transfer agreement with LiveOnNY.

Six donors were processed with a uniform protocol at Cambridge university where solid tissues were cut into small pieces, then homogenised with enzymatic digestion for 2x 15 minute heating/mixing steps at 37°C. The remaining six donors were subjected to a tissue adapted protocol with the aim of improving immune cell recovery, and this protocol was harmonised as closely as possible between the two collection sites.

For scRNA-seq experiments, single cells were loaded onto the channels of a Chromium chip (10x Genomics). cDNA synthesis, amplification, and sequencing libraries were generated using either the Single Cell 5′ Reagent (v1 and v2) (Cambridge University) or 3′ Reagent (v3) (Columbia University) Kit. TCRαβ, BCR and TCRγδ paired VDJ libraries were prepared from samples made with the 5′ Reagent kit. All libraries were sequenced on either a HiSeq 4000 or NovaSeq 6000 instrument.

### scRNA-seq and scVDJ-seq data analysis

scRNA-seq data was aligned and quantified using the cellranger software (version 6.1.1, 10x Genomics Inc.). Cells from hashtagged samples were demultiplexed using Hashsolo (67). Cells with fewer than 1,000 UMI counts and 600 detected genes were excluded. Doublets were detected using Scrublet (68). Downstream analysis from data normalization to graph-based clustering were performed using Scanpy (version 1.6.0) (69), with details described in Supplementary Materials. Data integration was done using BBKNN (70) and scVI (71), and the results were compared using kBET (72).

scTCR-seq and scBCR-seq data were aligned and quantified using the cellranger-vdj software (version 2.1.1 and 4.0, respectively). For TCRγδ we implemented a customized pipeline (https://sc-dandelion.readthedocs.io/en/latest/notebooks/gamma_delta.html) due to cellranger being tuned towards alpha/beta TCR chains. scTCR-seq analysis including productive TCR chain pairing and clonotype detection was performed using the scirpy package (73).

### CellTypist

Details of CellTypist, including cross-data cell type label harmonization and automated cell annotation, can be found in Supplementary Text. Briefly, immune cells from 20 tissues of 19 studies were collected and harmonised into consistent labels. These cells were split into equal-sized mini-batches, and these batches were sequentially trained by the l2-regularized logistic regression using stochastic gradient descent learning. Feature selection was performed to choose the top 300 genes from each cell type, and the union of these genes were supplied as the input for a second round of training.

### Single molecule FISH, flow cytometry, qPCR and Immunofluorescence

For single molecule FISH, samples were run using the RNAscope 2.5 LS fluorescent multiplex assay (automated). Slides were imaged on the Perkin Elmer Opera Phenix High-Content Screening System, in confocal mode with 1 μm z-step size, using 20X (NA 0.16, 0.299 μm/pixel) and 40X (NA 1.1, 0.149 μm/pixel) water-immersion objectives.

For flow cytometry, mononuclear cells (MNCs) were either stained *ex vivo* or post activation with PMA+I for two hours. Cells were stained with the live/dead marker Zombie Aqua for 10 minutes at room temperature, and then washed with PBS+0.5%FCS, with the CD8 and B cell panels of antibodies.

qPCR was performed in three spleen samples. Cells were stained with the live/dead marker Zombie Aqua for 10 minutes at room temperature, and then washed with PBS+0.5%FCS, followed by staining with the antibodies at 4°C for 45 minutes. Cell sorting was performed on a BD Fusion 4 laser sorter and RNA was extracted using a Zymo Research RNA micro kit.

For immunofluorescence, samples were fixed in 1% paraformaldehyde for 24 hours followed by 8 hours in 30% sucrose in PBS, and were stained for 2h at RT with the appropriate antibodies, washed three times in PBS and mounted in Fluoromount-G® (Southern Biotech). Images were acquired using a TCS SP8 (Leica, Milton Keynes, UK) confocal microscope.

## Supplementary Material

Supplementary Materials

## Data Availability

raw single-cell sequencing data have been deposited in the ArrayExpress database at EMBL-EBI (www.ebi.ac.uk/arrayexpress) under accession number E-MTAB-11536. Processed data can be downloaded and interactively explored at https://www.tissueimmunecellatlas.org. Code used for data analysis can be found on Zenodo at https://doi.org/10.5281/zenodo.6334988 (74).

## One Sentence Summary

We provide an immune cell atlas of human innate and adaptive immune cells across lymphoid, mucosal, and exocrine sites revealing tissue-specific compositions and features, and introduce CellTypist, a machine learning tool for rapid and precise cell type annotation.
