# Supplementary material for: Cross-tissue immune cell analysis reveals tissue-specific features in humans
Source: Science. Author manuscript; Available in PMC 2022 May 17. (PMC7612735; doi:10.1126/science.abl5197)
Supplement: Supplementary Materials [file EMS144957-supplement-Supplementary_Materials.pdf]

Science AAAS

Supplementary Materials for

Cross-tissue immune cell analysis reveals tissue-specific features in humans

Domínguez Conde C[1,#], Xu C[1,#], Jarvis LB[2,#], Rainbow DB[2#], Wells SB[3,#], Gomes T[1], Howlett SK[2], Suchanek O[4], Polanski K[1], King HW[5], Mamanova L[1], Huang N[1], Szabo PA[6], Richardson L[1], Bolt L[1], Fasouli ES[1], Mahbubani KT[7], Prete M[1], Tuck L[1], Richoz N[4], Tuong ZK[1,4], Campos L[1,8], Mousa HS[2], Needham EJ[2], Pritchard S[1,], Li T[1,], Elmentaite R[1,], Park J[1,], Rahmani E[9,10], Chen D[3], Menon DK[11], Bayraktar OA[1], James LK[5], Meyer KB[1,], Yosef N[9,10,12,13], Clatworthy MR[1,4], Sims PA[3], Farber DL[6], Saeb-Parsy K[7,]*, Jones JL[2,]*, Teichmann SA[1,14,]*

correspondence to: st9@sanger.ac.uk, jls53@medschl.cam.ac.uk, ks10014@cam.ac.uk

This PDF file includes:

Materials and Methods
Supplementary Text
Figs. S1 to S26
Tables S1 to S3

Materials and Methods

Tissue acquisition

All work was completed under ethically approved studies. Tissue was obtained from deceased organ donors following circulatory death (DCD) or brain death (DBD) via the Cambridge Biorepository for Translational Medicine (CBTM, https://www.cbtm.group.cam.ac.uk/), REC 15/EE/0152. Briefly, donors proceeded to organ donation after cessation of circulation. Organs were then perfused *in situ* with cold organ preservation solution and cooled with topical application of ice. Samples for the study were obtained within 60 minutes of cessation of circulation and placed in University of Wisconsin (UW) organ preservation solution for transport at 4°C to the laboratory. Gut samples were taken from the locations indicated in Fig. 1A. Additional samples were obtained from the left lower lobe of the lung and the right lobe of the liver. Skeletal muscle was taken from the third intercostal space and bone marrow was obtained from the vertebral bodies. In addition, two donor-matched blood samples were taken just prior to treatment withdrawal, under REC approval 97/290.

At Columbia University, human tissues were obtained from deceased organ donors at the time of organ acquisition for clinical transplantation through an approved protocol and material transfer agreement with LiveOnNY, the organ procurement organization (OPO) for the New York metropolitan area, as previously described (*54*, *75*). All donors were free of cancer and seronegative for hepatitis B, hepatitis C, and HIV. As tissues were obtained from brain-dead organ donors, this study does not qualify as "human subjects" research, as confirmed by the Columbia University Institutional Review Board.

Donor metadata which includes age, sex, cause of death, CMV/EBV/TOXO status and medication is described in Table S1.

Tissue processing

The first six donors recruited were processed with a uniform protocol. The remaining donors were processed with a tissue adapted protocol with the aim of improving immune cell recovery, and this protocol was harmonised as closely as possible between the Cambridge University and Columbia University collection sites. Immune cell composition was broadly similar between all protocols used (**fig. S12**).

Tissue processing for donors A29, A31, A35, A36, A37, A52 (Cambridge University)

Tissues from these donors were processed using a uniform protocol. Briefly, solid tissues were transferred to a 100mm tissue culture dish, cut into small pieces and transferred to Gentlemacs C-tubes (Miltenyi Biotec) at a maximum of 5g/tube in 5mL of X-vivo15 media (Lonza LZBE02) containing 0.13U/m Liberase TL (Roche 5401020001), 10U/mL DNase (benzonase nuclease, Merck 70746-4) supplemented with 2% (v/v) heat-inactivated fetal bovine serum (FBS; Merck F6178), penicillin and streptomycin (100 U/ml and 0.1 mg/ml, respectively, Sigma-Aldrich P0781), and 10mM HEPES (Sigma Aldrich H0887). The samples were then dissociated using a GentleMACS Octo dissociator (Miltenyi Biotec) using a protocol that provided gradual ramping up of homogenisation speed along with 2x 15 minute heating/mixing steps at 37°C. Digested tissue was filtered through a 70-µm MACS Smartstrainer (Miltenyi Biotec 130-098-462) and washed with media containing 2mM EDTA (ThermoFisher 15575020) , prior to washing with PBS (Merck D8537). A ficoll density centrifugation step (400g for 30min at RT) was performed to isolate mononuclear cells (MNCs). After gradient centrifugation, cells were washed once with PBS prior to counting and resuspending PBS containing 0.04% (v/v) BSA (Gibco 15260037).

Bone marrow aspirates and blood samples were diluted 1:1 with PBS and layered directly onto ficoll for mononuclear cell isolation as described above. Cells taken from the interphase layer were washed with PBS and exposed to the same enzymatic conditions as solid tissues by resuspending cell pellets in tissue dissociation media containing liberase TL for 30 minutes at 37°C prior to counting and resuspending in PBS containing 0.04% (v/v) BSA.

Tissue processing for donors 582C, 621B, 637C and 640C (Cambridge University)

Lymphoid tissues like spleen and lymph nodes were mashed through a 70 µM filter placed on top of a 50ml falcon, using the plunger from a 2 ml syringe as a pestle. The filter was occasionally washed with x-vivo + 1% FBS + 10U/ml Benzonase (Merck). Depending on the size of the tissue, the filtered cell suspension was topped up to 30-50ml with x-vivo + 1% FBS + 10U/ml Benzonase, and placed on ice as required.

Non-lymphoid tissue like lung, liver and kidney were first chopped up with scissors into 0.2-0.5cm pieces, then transferred into Gentlemacs C-tubes and 2.5ml of collagenase (Merck C7926) and 2.5 ml x-vivo media added, then homogenised using a protocol that provided gradual ramping up of homogenisation speed along with 2x 15 minute heating/mixing steps at 37°C. Post-gentlemacs homogenisation 20 µl of 0.5 mM EDTA (2mM final conc) per 5 ml of collagenase was added to neutralise, and the digested tissue transferred into a 70µM cell strainer placed on top of a 50 ml falcon. Using the plunger of a 2 ml syringe the tissue was mashed through the filter, with occasional washing of the filter with x-vivo + 1% FBS + 10U/ml Benzonase. Depending on the size of the tissue, the filtered cell suspension was topped up to 30-50ml with x-vivo + 1% FBS+ 10U/ml Benzonase, and placed on ice as required.

The protocol used to process jejunum was adapted from (*11*) with the aim of separating the lamina propria (LP) and intraepithelial layers (IEL), abbreviated to JEJLP and JEJEPI in the dataset. The jejunum was washed with PBS + 0.04% BSA to remove any chime, chopped up with scissors into 0.5 cm pieces then transferred into a 50 ml falcon tube and 10 ml of x-vivo + 2 mM DTT (0.1M solution, ThermoFisher 707265ML) + 5 mM EDTA (0.5M solution, Invitrogen) + 1% FBS added, then placed in the 37°C incubator for 20 minutes. The tube was shaken after 10 minutes. The jejunum chemical digest was then put through a 70µM filter placed on top of a 50 ml falcon and rinsed with 10 ml of x-vivo + 1% FBS + 10U/ml Benzonase. The wash through from the filter contains the IEL cells, and was kept on ice. Excess tissue from the filter was scraped back into a 50 ml falcon and the digest and filtering steps repeated. Remaining tissue from the filter was next scraped into a Gentlemacs C tube and digested with 2.5 ml of

collagenase IV (Merck) and 2.5ml of x-vivo and run on the same Gentlemacs programme used for the non-lymphoid tissues. 20 µl of 0.5 mM EDTA (Invitrogen) to give a 2mM final concentration per 5 ml of collagenase was then added to neutralise and the digested tissue placed into a 70µM cell strainer placed on top of a 50 ml falcon. Using the plunger of a 2 ml syringe the tissue was mashed through the filter which was occasionally washed with x-vivo + 1% FBS + 10U/ml Benzonase. The cells that pass through the filter are LP cells. Depending on the size of the tissue, the filtered cell suspension was topped up to 30-50ml with x-vivo + 1% FBS + 10U/ml Benzonase (Merck), and placed on ice as required.

Once a single cell suspension was obtained from all tissues, they were centrifuged at 600g x 10 minutes and resuspended in x-vivo + 1% FBS ready for layering over ficoll. Blood and bone marrow was diluted 1:1 in x-vivo + 1% FBS and layered over ficoll with no additional processing steps. The mononuclear cell isolation using ficoll was performed as described above. This protocol is available on protocols.io (dx.doi.org/10.17504/protocols.io.bz4qp8vw).

Hashtag labeling of cells for donors 582C, 621B, 637C and 640C (Cambridge University)

Cells were hashtagged to allow pooling of samples for loading on the 10X Chromium instrument and the hashtags used are listed in Table S2. Approximately 500k MNC per tissue was transferred into a 1.5ml lo-bind eppendorf. Cells were spun at 600g for 5 minutes and as much supernatant as possible was removed and the cells resuspended in 50µl PBS+0.04% BSA. 5µl of FC block (BioLegend 422301) was added to reduce background labelling and incubated at 4ºC for 10 minutes. Each hashtag was spun at 14,000g for 10 minutes, and then added 0.5µl of hashtag to each tube. Incubate at 4ºC for 30 minutes then top up to 500µl with PBS + 0.04% BSA, and spin at 600g x 5 mins, and remove supernatant. Wash cells twice more with 500µl with PBS + 0.04% BSA, then resuspend cells in 100µl with PBS + 0.04% BSA. Count cells and pool equal numbers of cells from each tissue and proceed to loading of the 10X Chromium Controller.

Tissue processing for donors D496 and D503 (Columbia University)

Each tissue was subjected to a tissue specific protocol to maximize cell recovery and viability across a diversity of sites:

Blood samples and bone marrow aspirates shared a protocol in which they were diluted 1:4 and layered directly onto Ficoll-paque for a density centrifugation step (1200 x g for 20min at 20°C) and subsequent mononuclear cell isolation.

Spleen samples were mechanically digested with scissors, and mashed and washed through a 100µM filter with a solution of PBS containing 5% (v/v) FBS and 2mM EDTA. The single cell suspension was spun down (400 x g for 10 minutes at 20°C), washed with PBS containing 5% (v/v) FBS and 2mM EDTA, and layered onto Ficoll-paque for a density centrifugation step (1200 x g for 20min at 20°C) and subsequent mononuclear cell isolation.

Lung and all lymph node samples shared a protocol where they were mechanically digested with scissors and enzymatically digested on a shaker for 30 minutes at 37°C in IMDM Media (Gibco 12440053) containing 1mg/mL Collagenase D (Millipore Sigma 11088882001) and 0.1mg/mL DNase (Worthington LS002139). After digestion, the tissue was mashed and washed through a 100µM filter with a solution of PBS containing 5% (v/v) FBS and 2mM EDTA. The single cell suspension was spun down (400 x g for 10 minutes at 20°C), washed and resuspended with IMDM Media with 10% (v/v) FBS, and layered onto Ficoll-paque for a density centrifugation step (1200 x g for 20min at 20°C) and subsequent mononuclear cell isolation.

Jejunum tissue was processed to separate the Epithelial Layer (EL) from the Lamina Propria (LP) abbreviated to JEJEPI and JEJLP in the dataset respectively. The process begins by washing the tissue of intestinal contents or chyme with cold PBS containing 5% (v/v) FBS. The EL was stripped by twice incubating the tissue at 37°C on a shaker for 30 minutes with IMDM Media containing 2 mM DTT, 10 mM EDTA, and 10% (v/v) FBS. After each strip, the media was removed from the tissue, filtered, and washed through a 100µM filter with a solution of PBS containing 5% (v/v) FBS and 2mM EDTA to collect the EL fraction, which is stored on ice until the LP fraction has

been collected. In order to collect the cells of the LP, after the second stripping step, the tissue was mechanically digested with scissors and enzymatically digested on a shaker for 30 minutes at 37°C in IMDM Media (Gibco 12440053) containing 1mg/mL Collagenase D (Millipore Sigma 11088882001) and 0.1mg/mL DNase (Worthington LS002139). After digestion, the tissue was mashed and washed through a 100μM filter with a solution of PBS containing 5% (v/v) FBS and 2mM EDTA. At this step, the single cell suspensions of both the EL and LP fractions were spun down (400 x g for 10 minutes at 20°C), washed and resuspended in IMDM Media with 0.25U/ml Benzonase (Millipore Sigma E1014-5KU). The samples were incubated for 30 minutes at 37°C, washed and resuspended with IMDM Media with 10% (v/v) FBS, and layered onto Ficoll-paque for a density centrifugation step (1200 x g for 20min at 20°C) and subsequent mononuclear cell isolation.

Once isolated, all single cell suspensions were centrifuged (400 x g, 10 minutes at 4°C) and washed twice with PBS containing 5% (v/v) FBS and 2mM EDTA. Cell counts were acquired using the NC-2000 Cell Counter (Chemometec) and 50 million viable cells from each site were treated with Trustain FcX (BioLegend 422302) and FcR Blocking Reagent (Miltenyi 130-059-901). Cells were subsequently labeled for 30 minutes at 4°C with biotinylated anti-CD66B (BioLegend 305120), anti-CD235ab (BioLegend 306618), anti-CD326 (BioLegend 324216) to remove granulocytes, red blood cells, and epithelial cells respectively via streptavidin-coated magnetic particles and negative selection (Bangs Laboratories BP628). Finally, all single cell suspensions were subjected to dead cell removal using a Dead Cell Removal Kit (Miltenyi).

Hashtag labeling of cells for donors D496 and D503 (Columbia University)

Each single cell suspension was hashtagged to allow pooling of samples for loading on the 10X Chromium instrument and the hashtags used are listed in Table S2. Approximately one million MNC per tissue were transferred into 4mL flow cytometry tubes. Cells were centrifuged at 400 x g for 5 minutes, 4°C, supernatant removed, and resuspended in PBS containing 5% (v/v) FBS and 2mM EDTA. Cells were treated with Trustain FcX (BioLegend 422302) and FcR Blocking Reagent (Miltenyi 130-059-901)

to reduce background labelling and incubated at 4°C for 10 minutes. Each hashtag was spun at 14,000 x g for 10 minutes, and 1µL of hashtag was added to each tube. The samples were incubated at 4°C for 30 minutes, and subsequently centrifuged at 400 x g for 5 minutes, 4°C and washed three times with PBS containing 5% (v/v) FBS and 2mM EDTA. 200K from each sample was added to a single 4mL flow cytometry tube. This tube was centrifuged at 400 x g for 5 minutes, 4°C and resuspended in PBS containing 5% (v/v) FBS and 2mM EDTA.

Single-cell RNA library preparation and sequencing

For scRNA-seq experiments, single cells were loaded onto the channels of a Chromium chip (10x Genomics) for a target recovery of 5,000 cells. Immune cells from donors A29, A31, A35, A36, A37, A52 were loaded into a single 10x channel per tissue per donor. For the remaining donors, as the cells were hashtag labelled, each donor's cells were pooled and loaded into a maximum of sixteen 10x channels. Single-cell cDNA synthesis, amplification, and sequencing libraries were generated using either the 10X Genomics Single Cell 5′ Reagent (v1 1000006 and 1000020 and v2 1000263 and 1000190) (Cambridge University) or 3′ Reagent (v3.1 1000121) (Columbia University) Kit from 10x Genomics following the manufacturer's instructions. The gene expression libraries were sequenced on an Illumina NovaSeq 6000 platform at a target depth of 50,000 reads per cell. For samples prepared with the Single Cell 5′ Reagent Kit, VDJ libraries for TCRαβ and BCR were prepared with the V(D)J enrichment Kit from 10x Genomics (v1 1000005 and 1000016 and v2 1000252 and 1000253) following the manufacturer's instructions. VDJ libraries for TCRγδ were prepared using previously published primers (*76*) compatible with the Single Cell 5′ Reagent (v1) Kit from 10x Genomics. VDJ libraries for B and T cells were sequenced on either a HiSeq 4000 or pooled with gene expression libraries on the NovaSeq 6000 platform at a target depth of 5,000 reads per cell. Hashtag libraries were pooled and sequenced on either an Illumina NextSeq 500, Illumina HiSeq 4000 or pooled with gene expression libraries on the NovaSeq 6000 platform.

Single-cell RNA-seq data pre-processing

scRNA-seq data was aligned and quantified using the cellranger software (version 6.1.1, 10x Genomics Inc.) using the GRCh38 human reference genome (official Cell Ranger reference, version 1.2.0). For hashtagged and multiplexed samples (applies to donors 582C, 621B, 637C, 640C, D496 and D503) hashtag-based cell demultiplexing was performed using Hashsolo (*67*). Cells with fewer than 1,000 UMI counts and 600 detected genes were excluded from downstream analysis. scTCR-seq data was aligned and quantified using the cellranger-vdj software (version 2.1.1, 10x Genomics Inc). scBCR-seq data was aligned and quantified using the cellranger-vdj software (version 4.0, 10x Genomics Inc). For TCRγδ we implemented a customized pipeline due to the cellranger vdj annotation algorithm being tuned towards alpha/beta TCR chains. Briefly, TCRγδ libraries were mapped with cell ranger 4.0.0, using the 10x VDJ 4.0.0 reference. All contigs deemed high quality were selected, and reannotated with IgBlast via the workflow provided in dandelion 0.1.3 (https://github.com/zktuong/dandelion). We have made available an example notebook showcasing this workflow (https://sc-dandelion.readthedocs.io/en/latest/notebooks/gamma_delta.html).

Doublet detection

Doublet detection was performed on a per sample basis using the Scrublet algorithm (https://github.com/AllonKleinLab/scrublet (*68*)) with percolation as previously described (*77*). Briefly, scrublet scores were obtained per cell and the percolation step was performed on over-clustered data using the *scanpy.tl.louvain* function from the scanpy package. Each cluster was subsequently separately clustered again, yielding an over-clustered manifold, and each of the resulting clusters had its Scrublet scores replaced by the median of the observed values. The resulting scores were assessed for statistical significance, with P values computed using a right-tailed test from a normal distribution centred on the score median and a median absolute deviation (MAD)-derived standard deviation estimate. The P-values were corrected for false discovery rate with the Benjamini-Hochberg procedure, and a significance threshold of

0.1 was imposed. Cells with a Benjamini-Hochberg-corrected P-value less than 0.1 were excluded from downstream analysis.

Clustering, batch alignment and annotation

Downstream analysis included data normalisation (*scanpy.pp.normalize_per_cell* method, scaling factor 10,000), log-transformation (*scanpy.pp.log1p*), variable gene detection (*sc.pp.highly_variable_genes*), data feature scaling (*scanpy.pp.scale*), PCA analysis (*scanpy.pp.pca*, from variable genes), and Leiden graph-based clustering (*scanpy.tl.leiden*, clustering resolution manually adjusted) performed using the python package scanpy (version 1.6.0). Data integration across donors was done using batch-balanced KNN (https://github.com/Teichlab/bbknn) (*70*). Prior to donor integration, batch correction for chemistry-associated effects was performed using ridge regression as implemented in the BBKNN package. Cell identities were first predicted using CellTypist, and then underwent manual curation including: 1) examination of expression of known marker genes and marker genes derived from CellTypist models; 2) cross-validation and cross-prediction with CellTypist training datasets and two independent datasets from the human gut and lung (*27*, *28*). Differential expression across clusters was assessed using the *sc.tl.rank_gene_groups* function from scanpy using the wilcoxon rank sum method. To achieve a high-resolution annotation, we sub-clustered ILC/T, B and myeloid cells and repeated the procedure of highly variable gene selection, which allowed for fine-grained cell type annotations.

Comparisons of BBKNN integration with scVI

To examine the influence of alternative data integration methods on our dataset, scVI was used to integrate the dataset with information of different donors as a covariate. Specifically, we extracted the same set of highly variable genes as used in BBKNN, and set up the input data with a raw count matrix. For the scVI model, the number of latent representations was set as 20, and the dropout rate was set as 0.2. We obtained the ultimate latent variables after 500 epochs of training (*max_epochs=500* and

early_stopping=True) which were input to the Scanpy pipeline for neighborhood graph construction (*sc.pp.neighbors*) and generation of UMAP coordinates (*sc.tl.umap*).

To assess the batch-mixing effect after BBKNN (**fig. S24A**) and scVI integration (**fig. S24B**), we conducted the k-nearest neighbour batch effect test (kBET) (*72*) to assess the degree of batch mixing for each cluster (**fig. S24C**) and cell type (**fig. S24D**). Clusters were derived from respective neighborhood graph output by BBKNN or scVI using a resolution of 1, and cell types were based on CellTypist predictions. For each method, the resulting KNN graph was used as the input for kBET to reflect the post-batch-correction distance between cells, and kBET observed rejection rates were calculated using the function *kBET* from kBET. This analysis revealed that high kBET acceptance rates can be obtained after data integration using both methods, and that BBKNN slightly outperformed scVI in our case.

<u>scTCR-seq downstream analysis</u>

VDJ sequence information was extracted from the output file "filtered_contig_annotations.csv" using the scirpy package (*73*). We determined productive TCR chain pairing features using the *scirpy.tl.chain_pairing* function and selected cells with a single pair of productive αβ TCR chains for downstream analysis. Clonotypes were determined using the *scirpy.pp.tcr_neighbors* function using the CDR3 nucleotide sequence identity from both TCR chains as a metric.

<u>scBCR-seq downstream analysis</u>

VDJ sequence information was extracted from the output file "filtered_contig_annotations.csv". Further single-cell VDJ analysis for B cells was performed broadly as described previously (*11*, *78*), with all sequences from a given patient grouped together for analysis. AssignGenes.py (*79*) and IgBLAST (*80*) were used to reannotate IgH sequences prior to correction of ambiguous V gene assignments using TIgGER (v1.0.0) (*81*). Clonally-related IgH sequences were identified using DefineClones.py with a nearest neighbour distance threshold of 0.15 before running

CreateGermlines.py (ChangeO) (*82*) to infer germline sequences for each clonal family and calculate somatic hypermutation frequencies with observedMutations (Shazam) (*82*). IgH diversity analyses were performed using the rarefyDiversity and testDiversity of Alakazam (v1.0.2; (*82*)). scVDJ sequences were then integrated with single-cell gene expression objects by determining the number of high quality annotated IgH, IgK or IgL per unique cell barcode. If more than one contig per chain was identified, metadata for that cell was ascribed as “Multi”. To assess clonal relationships between scRNA-seq clusters, co-occurrence of expanded clone members between cell types and tissues was reported as a binary event for each clone that contained a member within two different cell types or tissues in single-cell repertoires.

Single molecule FISH

Samples were either snap frozen in chilled isopentane (-70^{0}C) or fixed in 10% NBF, dehydrated through an ethanol series, and embedded in paraffin wax. Samples were run using the RNAscope 2.5 LS fluorescent multiplex assay (automated). Briefly, FFPE tissue sections (5 μm) and fresh frozen tissue sections (10um) were cut. Fresh frozen tissues were pre-treated offline (4% PFA fixation 4°C 15 mins followed by 90mins at room temperature, sequential dehydration steps (50%, 70%, 100%, 100% ethanol, air dry)) and protease III was used. FFPE tissues required no pretreatment offline, but a Heat Induced Epitope Retrieval (HIER) step was performed by the instrument for 15mins using Epitope Retrieval 2 (ER2) at 95°C. These tissues also had protease III treatment. RNAscope probes used were from adcbio and included Hs-CD3D-C2 (599398-C2), Hs-CD8A-C3 (560398-C3), Hs-CRTAM (430248), Hs-AIRE (551248), Hs-ITGAX-C2 (419158-C2) and Hs-CCR7-C3 (410721-C3). Opal fluorophores (Opal 520, Opal 570 and Opal 650) were used at 1:300 dilution. Slides were imaged on the Perkin Elmer Opera Phenix High-Content Screening System, in confocal mode with 1 μm z-step size, using 20X (NA 0.16, 0.299 μm/pixel) and 40X (NA 1.1, 0.149 μm/pixel) water-immersion objectives.

Flow cytometry

Spleen, bone marrow and thoracic lymph nodes from additional donors different to the scRNA-seq study were used to validate the discovered cell populations. The MNCs were either stained ex vivo or post activation with PMA+I (eBioscience, Cell Stimulation Cocktail) for two hours. Cells were washed with PBS and then stained with the live/dead marker Zombie Aqua for 10 minutes at room temperature, and then washed with PBS+0.5%FCS. The MNCs were stained in PBS+0.5% FCS at 4°C for 45 minutes with the following panels of antibodies:

CD8 panel: CD3-BUV395 (SK7, BioLegend), CD56-BUV737 (NCAM16.2, BD Horizon), CCR9-BV421 (L053E8, BioLegend), CD4-BV605 (OKT4, BioLegend), TCRgd-Fitc (B1.1, Invitrogen), CX3CR1-PE (2A9-1, BioLegend), CRTAM-PECy7 (CR24.1, Invitrogen), CD16-APC (3G8, BioLegend), CD8-APCCy7 (RPA-T8, BioLegend).

B cell panel: IgD-BUV395 (IA6-2, BD Horizon), CCR7-BV421 (G043H7, BioLegend), CD3-BV605 (SK7, BioLegend), CD11c-BV785 (3.9, BioLegend), CD27-PE (0323, eBioscience), CD19-APC (HIB19, BioLegend), Tbet-PECy7 (eBio4810, eBioscience, Tbet staining was done after the surface staining using the eBioscience Foxp3 transcription factor staining buffer kit).

Cells were fixed with PBS+0.25%PFA and stored at 4°C until they were run on the Fortessa flow cytometry instrument, located in the Cambridge NIHR BRC Cell Phenotyping Hub. Spleen MNCs were used for single stain controls to calculate compensation and FMOs were used to calculate background fluorescence. FlowJo was used to analyse the flow cytometry data.

qPCR

qPCR was performed to validate the existence ITGAD-expressing γδ T cells in the spleen using three additional samples that were different to those used in the scRNA-seq

study. Spleen MNCs were stained with the live/dead marker Zombie Aqua for 10 minutes at room temperature, and then washed with PBS+0.5%FCS. Cells were then stained with the following antibodies at 4°C for 45 minutes: CD56-BV421 (HCD56, BioLegend), CD4-BV605 (OKT4, BioLegend), TCRgd-Fitc (B1.1, Invitrogen), TCRab-PerCPCy5.5 (IP26, BioLegend), CD52-PE (MHCD5204, Life Technologies), CD127-PECy7 (eBioRDR5, eBioscience), CD8-APC (RPA-T8, BioLegend), CD3-APCfire (UCHT1, BioLegend). Cells were washed with PBS+0.5% FCS and passed through a Celltrics (Partec) 30 μm filter prior to cell sorting. Cell sorting was performed on a BD Fusion 4 laser sorter and an example of the gating strategy used is shown in (**fig. S22C**).

Sorted cells were pelleted at 600g for 5 minutes and lysed in RNA lysis buffer and frozen until RNA could be extracted. RNA was extracted using a Zymo Research RNA micro kit with on column DNAse digestion. The standard protocol was followed and RNA eluted in 11 μl of water. This RNA was then used to make cDNA using both oligo dT and random hexamer primers with the reverse transcriptase SuperscriptIII. Probes to B2M (housekeeping gene) and two assays to ITGAD were purchased from ThermoFisher, and qPCR reactions were performed in duplicate with the following recipe: 8 μl master mix, 0.7 μl probe, 4.3 μl water and 3 μl cDNA. A ThermoFIsher QuantStudio7 instrument was used for the qPCR, and the Ct values were determined with the DCt being calculated as the Ct of ITGAD - Ct of B2M.

<u>Immunofluorescence</u>

Spleen and thoracic lymph node samples from unrelated donors were fixed in 1% paraformaldehyde (Electron Microscopy Services, 50-980-487) for 24 hours followed by 8 hours in 30% sucrose in PBS. 30μm sections were permeabilized and blocked in 0.1M TRIS, containing 0.1% Triton (Sigma, T8787-50ML), 1% normal mouse serum (Invitrogen, 10410), 1% normal rat serum (Invitrogen, 10710C) and 1% BSA (R&D Systems, DY995). Samples were stained for 2h at RT in a wet chamber with the appropriate antibodies, washed 3 times in PBS and mounted in Fluoromount-G®

(Southern Biotech, 0100-01). Images were acquired using a TCS SP8 (Leica, Milton Keynes, UK) confocal microscope. Raw imaging data were processed using Imaris (Bitplane).

Antibodies used: CD3-AF488, clone UCHT1, 1/100 dilution (BioLegend, 300415); CD1c-PE, clone L161, 1/50 dilution (BioLegend, 331505); CCR7-PE, clone 3D12, 1/50 dilution (eBioscience, 12-1979-42); CD19-AF594, clone HIB19, 1/100 dilution (BioLegend, 302250); CD11c-APC, clone MJ4-27G12, 1/100 dilution (Miltenyi, 130-114-102); HLA-DR-AF647, clone TAL 1B5, 1/100 dilution (Abcam, ab223907). Nuclei were stained with Hoechst 33258, 1/10,000 dilution (Biotum, 40044).

Supplementary Text

CellTypist: an interpretable pan-tissue database and automated tool for cell type annotation

Rationale

With the growing size of single-cell RNA-sequencing (scRNA-seq) datasets and their wide applications in tissue and disease biology (*83*, *84*), fast and accurate cell type annotation becomes of crucial value in order to accelerate the interpretation of newly generated scRNA-seq datasets. A variety of approaches have been put forward to perform the matching of cell identities between datasets (*85*). However, there are few tools that can harbor all features critical to the classification of a cell type, including attributing a classification to cells that are not represented in the training dataset, distinguishing highly homogeneous cell populations, easily integrating the existing analysis workflow, and being scalable to large datasets.

Furthermore, in order to transfer cell type labels to a query dataset, most of the existing tools use particular published datasets with cell annotations from individual publications and transfer the cell labels from these reference data sets. The comprehensiveness and quality of the reference training dataset, as a result, is not guaranteed. Many cell compartments are shared across tissues, such as immune cells. For these types of cell states, it is more useful to build a reference database with cross-dataset and cross-tissue cell types including both organ-specific ones (e.g., tissue-resident macrophages like liver Kupffer cells, placenta Hofbauer cells and kidney-resident macrophages) and shared ones (e.g., monocytes). Several efforts have focused on building scRNA-seq references for cell type classification, such as a recent approach which integrated query datasets with the reference atlas using conditional neural network models (*86*).

In this study, we focused on immune cells and their large variety of subtypes. Immune cells are ubiquitous and mobile across tissues, with specific adaptations to corresponding local environments. This leads to a high degree of cell type heterogeneity, which is further augmented by other factors such as developmental

lineage dynamics. Despite this heterogeneity, immune cells can still be grouped into cell types characterized by expression of definitive markers, functional roles, and parent lineages. Therefore, both cross-tissue integration and domain-specific knowledge are necessary in order to assemble a high-quality and well-curated pan-tissue immune reference followed by transferring cell types from this reference to query datasets, providing organ-agnostic automated annotation of immune cell types within a single search.

Here we introduce CellTypist, a cell type database and server focused on immune cells in its first incarnation as well as a directly interpretable pipeline for automatic annotation of scRNA-seq data. CellTypist currently includes a wide assortment of immune cell types collected from 20 tissues across 19 studies, with these deeply curated cell types publicly available to the community. The prediction of CellTypist is based on logistic regression classifiers optimized by the stochastic gradient descent (SGD) algorithm. Extensive model tuning and optimization is performed to ensure its applicability, with the derived models easily updatable for further releases by incorporating new cell annotations, as well as by including non-annotated cells which in future iterations may be described as specific cell types. Notably, our current CellTypist release involves both low- and high-resolution models which classify cells with coarse and fine granularities, respectively. CellTypist can be readily installed and used from *https://github.com/Teichlab/celltypist* and the cell type resource is available at *https://www.celltypist.org*.

Dataset compilation and integration

We sought to assemble a cross-tissue immune reference as a training set to facilitate downstream cell type label transfer in an organ-agnostic manner. scRNA-seq data were collected from 19 publications covering 20 different tissues (**fig. S2A**). A raw count matrix was obtained for each dataset and subsequently combined across datasets based on their common genes.

In order to focus the model's training data on *bona fide* immune cells, the combined expression matrix was filtered to include only cells expressing *PTPRC*, a general marker for immune cells, as well as not expressing *EPCAM* and *PDGFRA*,

markers for epithelial cells and fibroblasts, respectively. In addition, for the datasets which were already annotated in the original publications, only cells identified as immune cell types were included. Exceptions to these rules were “Epithelial cells”, “Endothelial cells” and “Fibroblasts” which were retained in the reference dataset to serve as umbrella categories representing fall-backs for non-immune cell types. For each of these datasets, meta-information was also collected, including the tissues of origin, sequencing protocols, and original cell type annotations where possible.

To get an overview of this assembled immune atlas, we integrated the 19 datasets by correcting the confounders derived from batches across datasets and sequencing protocols using scVI (**fig. S2, B and C**) (*71*). Specifically, we set up the input data with a raw count matrix and covariate keys of “Dataset” and “Protocol”. For the scVI model, the number of latent representations was set as 20 (*n_latent=20*), and the dropout rate was set as 0.2 (*dropout_rate=0.2*). We obtained the ultimate latent variables after 500 epochs of training (*max_epochs=500* and *batch_size=1024*) which were input to the Scanpy pipeline for neighborhood graph construction (*sc.pp.neighbors(use_rep=‘X_scVI’)*) and generation of UMAP coordinates (*sc.tl.umap*). Our integrated atlas can be browsed at *https://www.celltypist.org/training-data-cellxgene/*.

Cell type label harmonization

In order to train CellTypist models using uniform cell type labels, cell identities across datasets were summarized into consistent names using a cell type label harmonization pipeline (**fig. S3**).

For the vast majority of cells we collected, they were previously annotated by the original studies. These cells were categorized into different cell types and subtypes with knowledge inputs from experts (see the *Acknowledgements* section for contributions from the CellTypist Annotation Team). These cell type labels encompass two levels of hierarchies: a high-hierarchy (low-resolution) level which includes a total of 32 broad cell types; and a low-hierarchy (high-resolution) level which comprises 91 detailed cell types and subtypes through subdivision of broad cell types. These two levels are arranged hierarchically, such that the low-hierarchy annotations are able to

consistently match corresponding high-level classes. This two-level knowledge-based system was adopted for several reasons. First and foremost, the numbers of data points for each tissue and cell compartment are limited in a tractable range, given that the entire initial training dataset of CellTypist is less than one million cells, and our cross-tissue immune resource is also less than half a million cells. This limited size of data means that the knowledge about the immune cells still trumps data-driven approaches. In future, as data sets expand and are added, we expect that building a cell type hierarchy by the inputs from both domain knowledge and large-scale scRNA-seq data can result in a refined and full cell type structure. However, at present, it is not sufficient to assign certain types in the hierarchy, such as the different subtypes of T cells whose transcriptomes are too similar to be organized in a hierarchy with >2 levels. Second, with CellTypist we seek to provide an immune cell atlas that includes an accurate and community-wide accepted "cell type encyclopedia". Restricting to fewer but high-quality and high-confidence hierarchies serves this purpose. Third, with limited knowledge, some newly discovered cell types are purely based on the transcriptomic data. Inserting such cell types into a hierarchy with many levels based on their gene expression patterns alone is a risk with respect to the strength of biological evidence supporting their origins.

To derive homogenized high-quality annotations, we refined the annotations of all CellTypist training datasets through the label harmonization pipeline, including cell type categorization, data integration, clustering, and external data validation, which together were organized into four control modules: removal, correction, subdivision and mining (**fig. S3**).

First, we removed cells that were mis-annotated by the original publications. Specifically, we integrated the same cell type from different datasets by scVI with batch covariates of different studies and sequencing protocols as in the section "*Dataset compilation and integration*", and after that, removed cells that did not belong to the given category. Using mast cells as an example, when we combined 10 sources of mast cells (**fig. S4A, upper**), the clusters 9 and 11 were transcriptomically separated from other cells (**fig. S4A, bottom**). To confirm this phenomenon with external validations,

we built two CellTypist models (see the section "*Model training*" for building CellTypist models) from the gut (*27*) and lung immune cell populations (*28*), and then transferred cell type labels from the two models to our training data sets. The prediction results consistently showed that cluster 9 is a plasma cell population and cluster 11 is a monocyte/macrophage population (**fig. S4B**). Moreover, their cell identities were supported by the canonical plasma cell marker *MZB1* and monocyte/macrophage marker *CD74* (**fig. S4C**). With all the above evidence, we removed clusters 9 and 11 from the mast cell population. This removal process was performed for several other cell types as well, such as the innate lymphoid cell (ILC) precursors (**fig. S4, D to F**).

Second, we corrected cell type labels for some cells that were mis-classified in original studies. Before this, integration of cells from the same cell type, unsupervised clustering, and generation of two independent models for external validations, were performed as above. Next, cell types that were misclassified mostly due to their transcriptomic similarity with other close cell types were corrected (i.e., relabeled). Using regulatory T cells as an example, in CellTypist we combined nine sources of regulatory T cells (**fig. S5A, upper**), and clustered them into 14 clusters (**fig. S5A, bottom**). Among these clusters, clusters 0, 1, 2, 5, 7 and 10, which mainly originated from two studies, were consistently predicted as naive/central memory CD4+ T cells by the two independent CellTypist models (**fig. S5B**). Expression of definitive regulatory T cell markers (*CTLA4* and *FOXP3*) also supported the exclusion of these cells as regulatory T cells (**fig. S5C**). Thus we relabeled these regulatory T cells as naive/central memory CD4+ T cells in the CellTypist training datasets. This correction process was performed for several other cell types as well, such as the ILCs (**fig. S5, D to F**).

Third, we subdivided some broad cell types into clear and community-recognized cell subtypes. Similarly, before this, cells from a given cell type were integrated and clustered, and two CellTypist models for external validations were built. Next, high-confidence cell subtypes with consistent predictions from the two CellTypist models and with evidence of well-established marker gene expression were subdivided from a broad cell type. Using the monocytes as an example, after we combined 12 sources of monocytes and clustered them into 15 clusters (**fig. S6A**), we

resolved two clear monocyte subpopulations: non-classical monocytes and classical monocytes (**fig. S6B**). Expression of non-classical monocyte marker *FCGR3A* (encoding CD16) and classical monocyte marker *CD14* (encoding CD14) were also in line with this subdivision scheme for monocytes (**fig. S6C**). In CellTypist we therefore subdivided these monocytes into non-classical and classical ones. This subdivision process was also applied to other cell types such as natural killer (NK) cells which comprised CD16+ and CD16- NK cells (**fig. S6, D to F**).

Fourth, we identified cell populations that were hidden within other cell types and neglected by original publications. Specifically, after cells from a given cell type were integrated, clustered and predicted using the same strategy as before, we located the hidden cell types and expanded their cell numbers and tissue distributions. For example, within the cytotoxic T cell populations which were combined from 14 sources and clustered into 16 clusters (**fig. S7A**), we found a MAIT cell population. This cell type, though transcriptomically similar with cytotoxic T cells, was confidently predicted out of the cytotoxic T cells using the CellTypist model trained from Madissoon et al., 2021 (*28*) (**fig. S7B**). Expression of MAIT cell markers *SLC4A10* and *TRAV1-2* was also prominent in these cells, further supporting their cell identity as MAIT cells (**fig. S7C**). By adding back these cells, we expanded the number of MAIT cells from 1,132 to 2,367, and thus improved its representation (i.e., cell type size) in the CellTypist training datasets and extended its tissue distribution to additional organs like the spleen. Other examples included rare germinal center B cells in the adult immune system for which we expanded the number from 391 to 516 by mining them out from the memory B cells (**fig. S7, D to F**).

Through all of these, we deeply homogenized the cell type annotations of the CellTypist training datasets and used them as the input for CellTypist training and for obtaining annotations for non-annotated cells (see below).

<u>Propagating annotations to non-annotated cells</u>

After label standardization, a small subset of cells included in CellTypist still had no designated cell type labels. These cells were also subject to the same expression filtering as in the section "*Dataset compilation and integration*". Given that the

non-annotated cells may contain similar cell types as those in the annotated cells, we next sought to minimize the label duplication and facilitate the incorporation of both known and yet-to-be-annotated cell identities into the CellTypist models (**fig. S3**). Specifically, the non-annotated cells from each combination of tissue and dataset were clustered independently using a canonical Scanpy pipeline. The resulting clusters were then compared with their predicted cell type labels which were inferred from the CellTypist models trained from the annotated cells (for details of model training, see the section of "*Model training*"). For a given cluster where at least 75% of its cells matched a specific low-hierarchy annotation label, the whole cluster was annotated as such, and meanwhile was assigned a corresponding high-hierarchy cell type label. For the remaining clusters where this condition was not met, we assigned them cell type labels at the high-hierarchy level where possible, through the same procedure as the low-hierarchy labels. This resulted in a final set of harmonized labels between non-annotated and annotated cells for a total of 738,647 cells, including 91 detailed cell subtypes corresponding to 32 broad cell types across different datasets and tissues (**fig. S8**).

<u>Model training</u>

Different classifiers for cell type predictions have been described (*85*, *87*). Of note, high performance can be achieved even when the classifiers are constructed using canonical machine learning methods, notably the logistic regression models (*88*, *89*). We based the models of CellTypist on a logistic regression framework with several adaptations.

First, randomly sampled mini-batches, instead of the whole training dataset, were used during the training procedure. This approach not only bypassed the possible memory excess when modelling our large dataset, but also ensured the fast convergence not readily available for datasets with hundreds of thousands of cells. Each mini-batch comprised 1,000 cells sampled from the whole dataset, and in a single epoch 100 mutually exclusive mini-batches were sequentially trained. This step was repeated 30 epochs, enabling the CellTypist models to see cell numbers with six orders of magnitude. In practice, the number of epochs needed will be fewer, with the

performance plateau reached within 10 epochs (1,000 iterations) (**Fig. 1C**), highlighting the usefulness of the mini-batch training approach in CellTypist. In CellTypist, we have also implemented a functionality to balance cell types within mini-batches. Specifically, during the mini-batch sampling, cells from a given cell type are sampled with a probability inversely proportional to the number of cells belonging to this cell type. This ensures that a rare cell type is sampled into the mini-batches with a higher probability, and close numbers of cell types will ultimately stay in the mini-batches (subject to the maximum number of cells that can be provided by a given cell type). We also tested how this option will influence our models and the downstream prediction, and found that with the cell types balanced in mini-batches, the model prediction result for our resource was similar (**fig. S25B**) to that based on randomly sampled mini-batches (**fig. S25A**), indicating a high prediction accuracy that is already achieved by the model with random mini-batch sampling. Some inconsistencies, mainly from the "Cytotoxic T cells" and "Tcm/Naive helper T cells", were also observed (**fig. S25C**), possibly resulting from the undersampling of these populations which had high intra-cell type heterogeneity due to the oversampling of other rare cell types.

Second, SGD algorithm was used in combination with the mini-batch training to derive the solutions of the model cost/loss function. This was implemented using the scikit-learn package in Python (*90*) by the "*partial_fit*" method from the class "*SGDClassifier*". SGD also allows for online training, meaning that if new data are fed, it can be easily incorporated into the model.

Third, L2 regularization was imposed on the logistic models to make the predictions more applicable to external query datasets. This also allows each gene in the model to have a weight of greater than 0 such that more genes can be utilized when predicting query data with varying numbers of input features. The regularization term (alpha) was chosen by training the models with alpha set to 0.01, 0.001, 0.0001, 0.00001 or 0.000001, and the alpha yielding the best performance on an independently left-out data (10% of the total dataset) was chosen as the optimal hyper-parameter. Ultimately, the alpha was set to 0.0001 for the low-hierarchy model and 0.001 for the high-hierarchy model.

Last, feature selection was conducted before the final models were trained. Specifically, we performed an initial training based on the entire gene set, and selected the top 300 genes from each class (cell type) by ranking the genes according to their absolute weights associated with the given class. After combining the genes from all the cell types, a total of 3,278 genes were obtained and later supplied as the input to a second round of training. This step effectively reduces the complexity of the sample space and emphasizes the major contributions of highly informative genes to the classification of cell identities.

Processing of training and query datasets

As the input for CellTypist model training, the datasets were normalized to 10,000 counts per cell and log-transformed (with a pseudocount of 1). While this step cannot fully resolve the sequencing depth-related batches, genes detected in the cells after this step will have more comparable expression scales across different datasets. Meanwhile, for the gene expression matrix in the query data, CellTypist will detect the expression matrix and transform it into the same format as the CellTypist training datasets (or report an error and require the user to input the same format) to again ensure gene expression comparability between the training and query datasets. Later, to enable the fast convergence using the optimal SGD learning rate, as well as to ensure a comparable scale of weights across genes when L2 regularization was applied, expression of each gene was standardized to a mean of zero and unit variance. In the meantime, the mean and standard variation of each gene during this step are recorded in the CellTypist models and will be applied to the shared genes in the query dataset. Through this, we are able to further minimize the differences in expression scales and sparseness across datasets.

To strengthen the applicability of CellTypist to datasets with different sequencing protocols, an automatic feature selection step (see the section "*Model training*") was performed to reduce the gene numbers in the CellTypist models. This is particularly important considering that even though different sequencing protocols have different gene expression sparseness, marker or driving genes of cell types are stable within the gene expression matrix across protocols. With such a feature selection step,

we restrict the cell type-determining signals to fewer but more informative genes and bypass the expression noises between training and query datasets.

Practically, in addition to utilizing the CellTypist models to predict our cross-tissue immune cell dataset in this study representing intermediate gene expression sparseness (10X, on average 1,932 expressed genes per cell), we examined the performance of CellTypist models on two other datasets: 1) 2,494 immune cells processed by SmartSeq2 from Travaglini et al., 2020 (*91*) representing rich gene expression (on average 2,555 expressed genes per cell). CellTypist prediction of this dataset revealed cell populations that corresponded well with the cell type labels provided by the original study (**fig. S10A**). Moreover, using CellTypist we added previously unappreciated information to the cell types identified. For example, while the original study roughly annotated a B cell cluster, with CellTypist we were able to identify it as a naive B cell population. This was also the case for the natural killer cells from the original study for which CellTypist predicted as the CD16+ natural killer subtype. 2) 103,766 blood and immune cells processed using sci-RNA-seq3 from Cao et al., 2020 (*92*) representing sparse gene expression (on average 414 expressed genes per cell). CellTypist successfully assigned cell type labels to these cells that matched well with the original labels provided by the publication (**fig. S10B**). Additional information was also revealed, such as the original "Erythroblasts" cell population which was predicted by CellTypist into "Mid erythroid" and "Late erythroid", and the "B cells" population which was predicted into "Pro-B cells", "Plasma cells" and "Naive B cells".

Notably, though we performed batch-correction and data integration of the training datasets (see the section "*Dataset compilation and integration*"), we didn't use the resulting batch-corrected expression matrix as the input for CellTypist model training, but instead relied on the normalized and scaled gene expression matrix for several reasons.

First, with the data processing steps of normalization and scaling, the gene expression scales across different batches have largely been made comparable. Though some batches are still seen, genes with different expression levels can be equally penalized when a L2 regularization term is applied to the logistic regression classifiers.

Moreover, after proper normalization and scaling, the SGD learning can converge towards the optimum of the loss function more quickly and accurately, which cannot be achieved by a batch-corrected matrix. Therefore, through our optimized logistic regression framework, the generalization of cross-batch predictions is significantly improved. A minor advantage is that running the normalization and scaling is much faster in training new models than using batch-correction methods which usually take a long time to get a fully batch-corrected result.

Second, the normalization and scaling for the training datasets can be easily reproduced in the query datasets. CellTypist recorded the mean and standard deviation of each gene during training, and applied these parameters to the shared genes in the query datasets. This is almost not possible with batch-correction methods where a batch-correction procedure in the training datasets is hard to reproduce in the query datasets. That is, batch-correction methods cannot ensure that the degrees of noise removal in training and query datasets from two independent runs are comparable (concatenating the training and query datasets together for a holistic batch-correction can alleviate this problem but will result in the information leak from the query datasets to the training datasets during model training, thereby skewing the cell type prediction).

Third, for the CellTypist training datasets, we have collected cell types from different sources with a variety of batches. This creates a scenario where cells from a given cell type already contain inter-batch variations. CellTypist prediction, under this context, is the procedure of judging whether cross-cell-type differences are significantly larger than within-cell-type variations. Moreover, each predicted cell will have a confidence score ranging from 0 to 1 to quantify the significance of cell type prediction (see the section "*Cell type prediction*" below). Therefore even when the query datasets are from a different batch as compared to the training datasets, the prediction result will possibly persist albeit with a decreased confidence score.

Fourth, for most batch-correction methods, a large proportion of genes are usually discarded during the correction process, namely, only highly variable genes (hvgs) are used for batch correction and data integration. While in CellTypist, we train the models using all expressed genes and still maintain high prediction accuracy. After

that, there is also an option in CellTypist to perform an automatic feature selection step based on the first round of CellTypist run by selecting the cell type-driving genes across all cell types. Using normalized and scaled gene expression matrix, instead of hvgs-based batch-corrected gene expression matrix, can fit in with this pipeline.

Nevertheless, to assess how batch effects can impact the performance of CellTypist models in practice, we extracted the batch-corrected expression matrix using scVI, and repeated the training pipeline in CellTypist with all other parameters being constant. This led to a new model trained from this batch-corrected expression matrix, which was subsequently used to predict cell types from our cross-tissue immune resource (**fig. S11**). The results showed that the predictions from the new model were quite similar with those based on normalized and scaled gene expression matrices (**fig. S11, A and B**). However, for a number of cell types, the new model yielded coarse or incorrect predictions. For instance, the "Type 1 helper T cells", "Tcm/Naive helper T cells", "CD8a/b(entry)", "Helper T cells", "Follicular helper T cells", "Regulatory T cells", and "Tem/Effector helper T cells" are now grossly predicted as "Tcm/Naive helper T cells" by the new model (**fig. S11C**), and the "ILC", "Cytotoxic T cells", "Migratory DCs", "Non-classical monocytes" and "Early MK" are incorrectly predicted as "Epithelial cells" (**fig. S11C**). We therefore reason that a normalized and scaled expression matrix is more suited to our CellTypist pipeline and is able to produce more accurate and fine-grained cell type predictions.

<u>Cell type prediction</u>

Before the prediction, the input query data was normalized to 10,000 counts per cell and log-transformed (with a pseudocount of 1). Only genes shared between the CellTypist model and the input data were used in the downstream prediction. For each gene, as noted in "*Processing of training and query datasets*", we standardized it by subtracting the mean and scaling the standard deviation using the corresponding mean and standard deviation recorded in the training step for that gene.

For each cell type involved in the model, the decision scores of the query cells are defined as the linear combination of the scaled gene expression and the model coefficients associated with the given cell type ("*decision_function*" from the class

"*SGDClassifier*" in sklearn), and the probabilities are calculated by transforming the decision scores with a sigmoid function ("*scipy.special.expit*" in scipy). The two metrics are recorded in CellTypist outputs. Next, the cell type with the maximal decision score (or probability) is selected as the predicted identity for the query cell. Of note, we trained the models with an one-vs-rest (OVR) strategy, resulting in multiple independent binary classifiers with their decision scores and probabilities being comparable among cell types. Different from the multinomial logistic regression framework where the probabilities of all cell types for a given query cell are constrained to a sum of 1 during training and directly output by the tools, in CellTypist these probabilities are calculated from the decision scores and later kept as is, enabling the examination of novel and ambiguous cell types in the query data.

Specifically, depending on what the users want to achieve, CellTypist has two modes during the prediction step (*mode='best match'* or *mode='prob match'*), with the former assigning the most likely cell type to a given query cell for the purpose of distinguishing between homogeneous cell types, and the latter assigning 0 (i.e., a novel cell type only in the query dataset), 1 (i.e., unique assignment), or >=2 (i.e., multi-label assignments) to a given query cell using a probability threshold (default to 0.5 in CellTypist, which is well applied in the logistic regression framework in practice).

<u>Over-clustering and majority voting</u>

The prediction step is performed to infer the identities of input cells, which renders the prediction of each cell independent. To combine the cell type predictions with the cell-cell transcriptomic relationships, CellTypist offers a majority voting approach based on the idea that transcriptionally similar cells in the query dataset are more likely to form a (sub)cluster regardless of their individual prediction outcomes. In this study, the query data was first over-clustered using the Leiden algorithm on the basis of an existing neighborhood graph in the input object ("*scanpy.tl.leiden*" in Scanpy) with the resolution set to 25. If no neighborhood graph exists for the input data, a neighborhood graph will be constructed before the over-clustering ("*scanpy.pp.neighbors*" in Scanpy). Each resulting subcluster was then assigned the identity supported by the dominant cell type predicted for this subcluster. Through this

step, distinguishable small subclusters will be assigned distinct cell type labels, and homogenous subclusters will be assigned the same labels and iteratively converge to a bigger cluster.

However, after the majority-voting step, there may still exist heterogeneous clusters due to the bias in technical confounders and algorithmic inability to further resolve a subcluster. To bypass this, CellTypist has a proportion threshold defined as the proportion of the dominant cell type required to name a given subcluster by this cell type. Specifically, if the proportion of the dominant cell type fails to pass this cutoff (for example, <70%, which means that the remaining cell populations occupy >30% of the total number of cells in a given subcluster), the whole subcluster will be assigned "Heterogeneous" by CellTypist. Moreover, CellTypist outputs two results: the predicted labels for individual cells, and the labels after majority voting local subclusters. Through this, if a subcluster is assigned "Heterogeneous", the users are able to check the composition of this subcluster and determine the confidence of this majority-voted cell type.

We then applied this proportion threshold to our cross-tissue immune resource, and located two "Heterogeneous" clusters which were previously annotated as "HSC/MPP" and "Early MK", respectively (**fig. S26A**). The first cluster is mainly composed of progenitor cells, including 51.6% of "HSC/MPP", 18.2% of "CMP", 11.3% of "GMP", 9.3% of "ELP", 3.2% of "Granulocytes", 2.3% of "Megakaryocyte precursor", 1.7% of "Neutrophil-myeloid progenitor", 1.4% of "Double-negative thymocytes", and 1% of "Early MK" (**fig. S26B**). The second cluster is a megakaryocyte population including 54.2% of "Early MK" and 45.8% of "Megakaryocytes/platelets" (**fig. S26C**). Therefore through this approach, we are able to identify heterogeneous subclusters in our resource. However, these cases of heterogeneous clusters are rare and a vast majority of clusters after over-clustering and majority voting by CellTypist are dominated by one cell type (**fig. S26A**), indicating the usefulness of the majority-voting approach.

Benchmarking with other label-transferring methods

We focused on the comparisons among five methods: CellTypist, traditional logistic regression (lr) classifier, support vector machine (svm) classifier, Azimuth (*93*), and scNym (*94*). To this end, 10,000 cells were randomly sampled from our compiled dataset as an independent test dataset. We further generated three training datasets with the sizes being 5,000, 50,000, and 250,000 cells respectively, through sampling the cells from the remaining dataset. This allows us to examine the effect of sizes of training datasets on the prediction accuracy, representing small, medium and big training datasets, respectively.

To make the comparisons unbiased across different methods, both the training and test data were properly preprocessed beforehand (the time used for preprocessing is not included in the benchmarking of running time): i) For CellTypist, lr and svm, the training data was normalized and scaled as in "*Processing of training and query datasets*". The test data was normalized in the same way while scaled using the recorded mean and standard deviation as in the section "*Cell type prediction*"; ii) For scNym, the training and test datasets were both normalized to 1,000,000 counts per cell as suggested by the scNym guidelines and then log-transformed (with a pseudocount of 1); iii) For Azimuth, the training and test datasets were both normalized to 10,000 counts per cell and log-transformed (with a pseudocount of 1). For all the five methods, we used the same set of highly variable genes extracted from the reference object ("*scanpy.pp.highly_variable_genes*" in Scanpy).

We split the whole label-transferring procedure into the "training" and "prediction" steps. Moreover, we define a "user time" as the time needed for a user to get their prediction results after supplying the query data to the programs. This is critical as the user time is more related with the user experience in practice. **fig. S14A** lists the detailed split for the five methods. Specifically, in CellTypist, lr and svm, the training steps are only dependent on the training data, while in scNym and Azimuth, the training steps rely on both the training and test data ("*scnym_api(task='train')*" in scNym, and "*FindTransferAnchors*" in Seurat, respectively). Therefore, from the perspective of a

user, the user time in CellTypist, lr and svm equals to the prediction time while to the sum of training and prediction time in scNym and Azimuth.

For each method, we recorded both the training and prediction time, as well as the predicted cell types for the test data. The performance was then assessed for each cell type separately using three metrics: precision ("*sklearn.metrics.precision_score*"), recall ("*sklearn.metrics.recall_score*"), and F1 score ("*sklearn.metrics.f1_score*").

The results show that when the training data size is small (5,000 cells), CellTypist has a comparable performance as compared to the traditional logistic regression, Azimuth and scNym, all of which outperform svm using our datasets (**fig. S13**). When the training data size is medium (50,000 cells) or large (250,000 cells), CellTypist has a similar performance with the traditional logistic regression and scNym, which is slightly better than Azimuth and much better than svm. Importantly, our mini-batch training approach with SGD learning dramatically decreases the time needed for the model training and thus represents a more scalable method for large-scale scRNA-seq datasets (**fig. S14B**). In terms of the user time, as with canonical machine learning methods, CellTypist predicts the query data much more efficiently and quickly than Azimuth and scNym (see the user time marked by asterisks in the **fig. S14B**), largely due to the independence between the data training and prediction steps in CellTypist.

<u>CellTypist performance on the cross-tissue immune reference</u>

We next examined the performance of CellTypist on our assembled immune cell atlas. For an independently left-out dataset (10%), the CellTypist models trained from the remaining dataset (90%) demonstrated the precision of 0.97 and 0.91 at the high- and low-hierarchy levels, respectively (**fig. S9A**). The recall scores were relatively lower, but still reached 0.88 and 0.84 at the two levels, respectively (**fig. S9B**). Further summarizing the two metrics into the F1 score, the CellTypist models overall exhibited the F1 scores of 0.95 and 0.89 at the two levels (**Fig. 1C**). Examination of the F1 score for each cell type annotated in the models revealed that part of the models' prediction errors came from a low number of cells associated with certain labels (**fig. S9C**), indicating a future need of collecting more rare cell types.

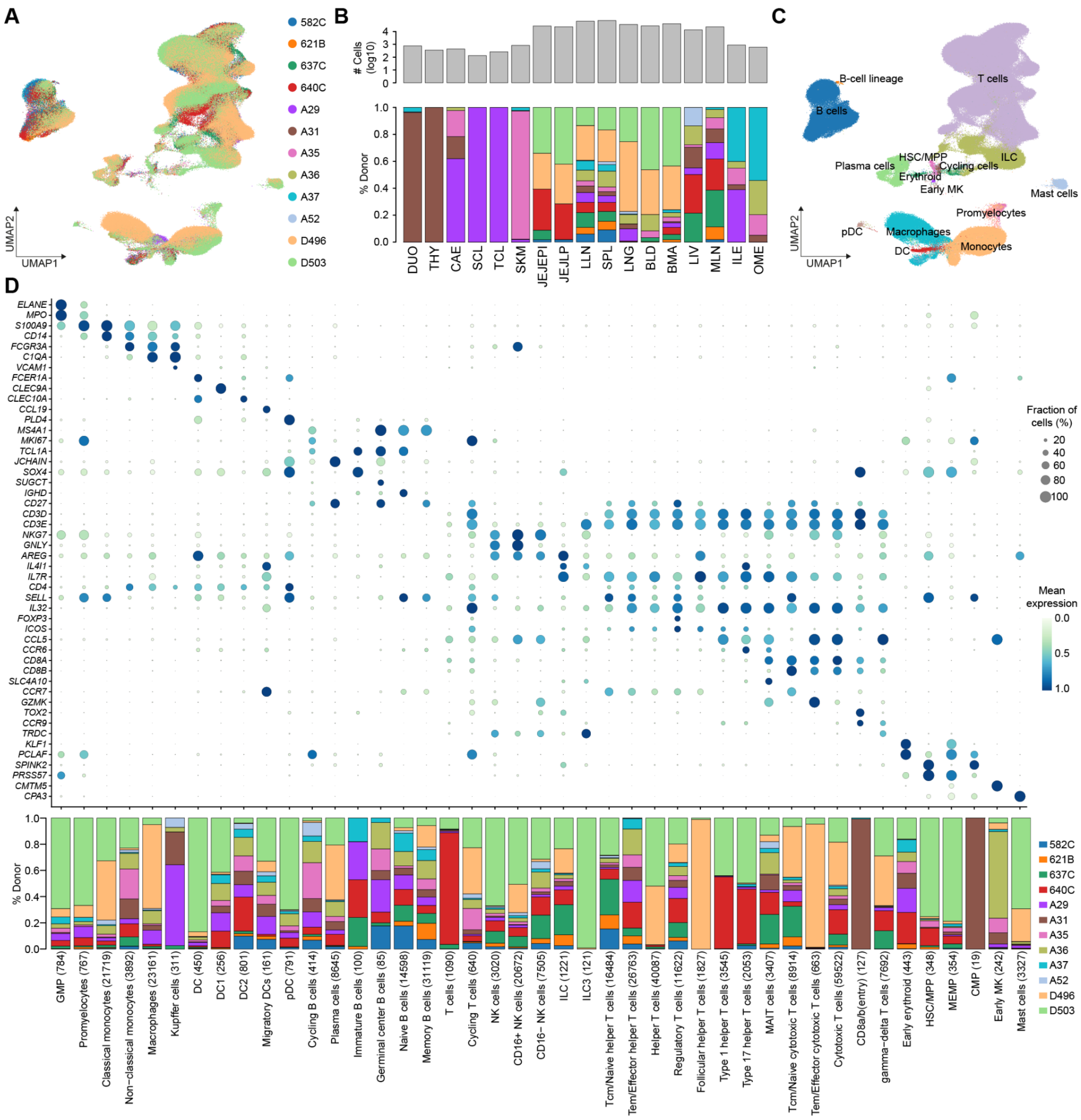

Fig. S1. Overview of the immune cell compartment of the dataset. (**A**) UMAP visualization of the immune cell compartment showing the donor distribution. (**B**) Bar plot showing the number of cells in each tissue (upper), as well as the stacked bar plot showing the percentages of donors per tissue (bottom). (**C**) As with (A), but colored by high-hierarchy cell types predicted using CellTypist. (**D**) Dot plot displaying the expression of CellTypist-derived marker genes for the predicted immune populations. Color gradient represents maximum-normalized mean expression of cells expressing the marker genes, and size represents the percentage of cells expressing these genes. The bottom stacked bar plot shows the number and percentage of donors across the predicted cell types.

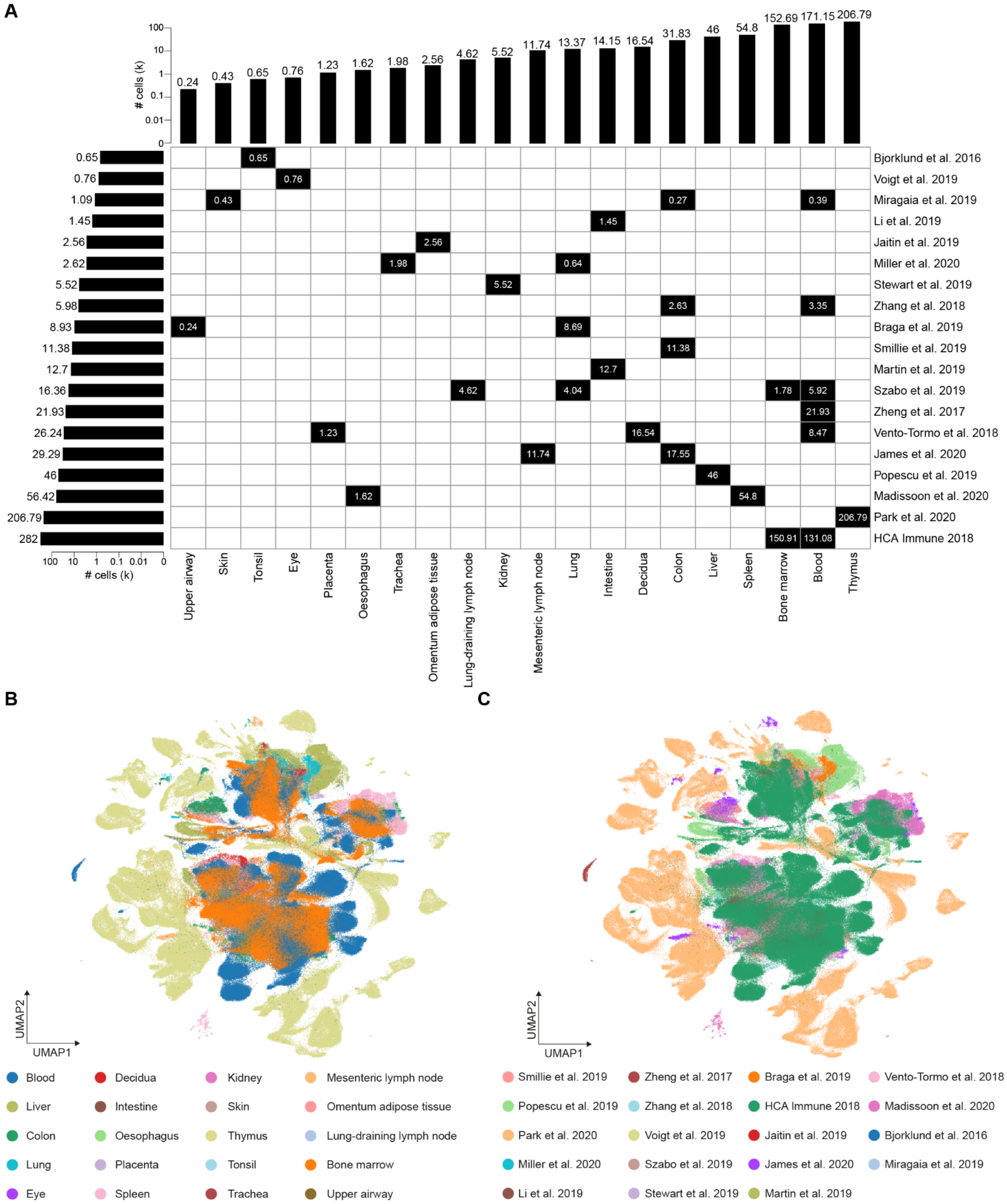

Fig. S2. Summary of the assembled immune atlas across tissues and datasets. (**A**) Heat map showing the number of cells in each combination of dataset (row) and tissue (column), as well as the total cell number in each dataset (horizontal bar plot) and each tissue (vertical bar plot). Cell numbers are denoted in units of thousands. (**B** and **C**) UMAP representations of the integrated immune cell atlas with information of tissues (B) and datasets (C) overlaid. Integration is performed using scVI with covariates of datasets and sequencing protocols.

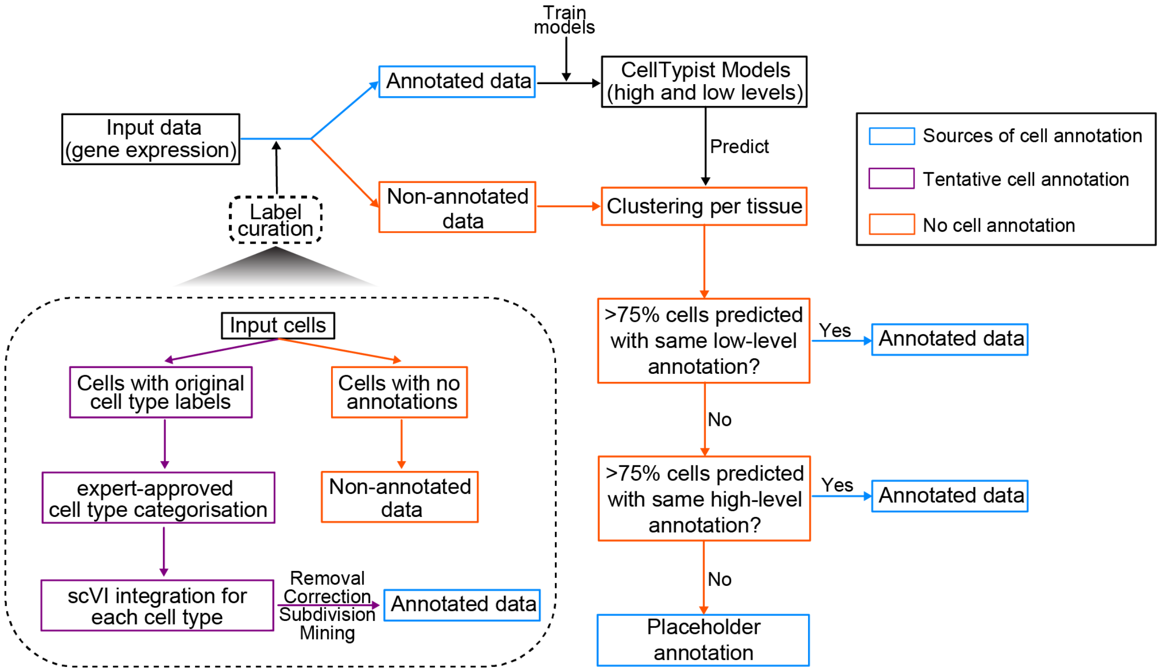

Fig. S3. Schematic of the CellTypist pipeline to harmonize cell type labels across training datasets. Among the input cells, annotated cells (i.e., cells with names and labels from original publications) are categorized into expert-approved cell types and cells belonging to a given cell type are further integrated and curated through four modules: removal, correction, subdivision and mining (see Supplementary Text). For unannotated cells, they are clustered and assigned the labels by training CellTypist models on annotated cells and later propagating cell type labels from the models to the unannotated cells.

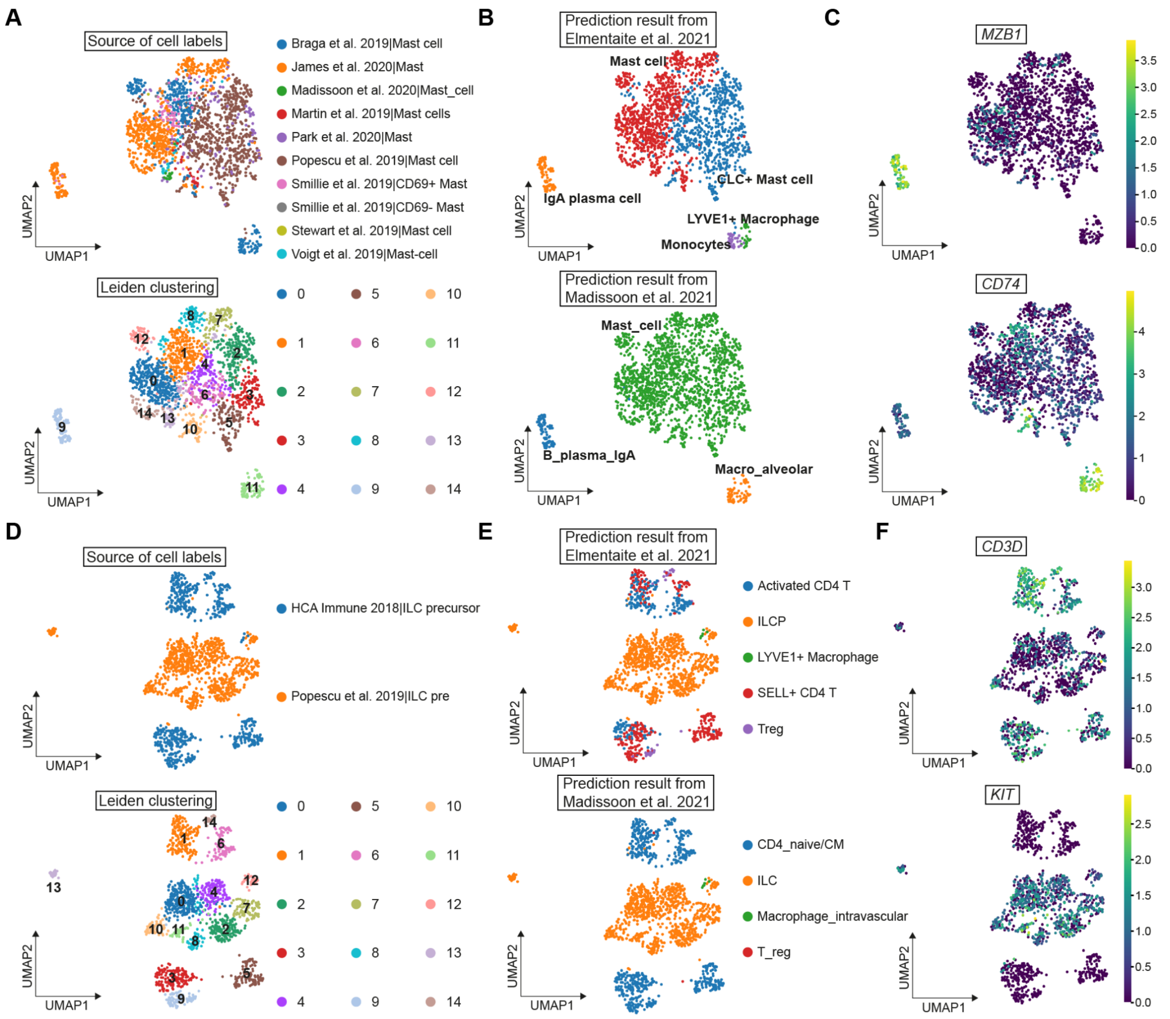

Fig. S4. Two examples of cell type label harmonization through removing cells that are incorrectly annotated by original publications. (**A**) UMAP visualizations of all mast cells in CellTypist training datasets with information of cell sources (datasets followed by original cell type labels, upper) and unsupervised clustering (Leiden clustering based on the neighbourhood graph constructed using scVI-derived latent space, bottom). (**B**) UMAP visualizations of the transferred cell type labels from Elmentaite et al., 2021 (*27*) (upper) and Madissoon et al., 2021(*28*) (bottom) by training the CellTypist models on the two datasets, respectively. Clusters 9 and 11 are consistently predicted as plasma cells and monocytes/macrophages, and thus can be removed from the mast cell category. (**C**) Expression of plasma cell marker *MZB1* (upper) and mononuclear phagocyte marker *CD74* (bottom) overlaid onto the UMAP representations, supporting the identities of clusters 9 and 11 as plasma cells and monocytes/macrophages, respectively. (**D** to **F**) As with (A), (B), and (C), but for innate

lymphoid cell (ILC) precursors. Only cells that are annotated as ILC precursors from Popescu et al., 2019 (*1*) are kept after the removal process.

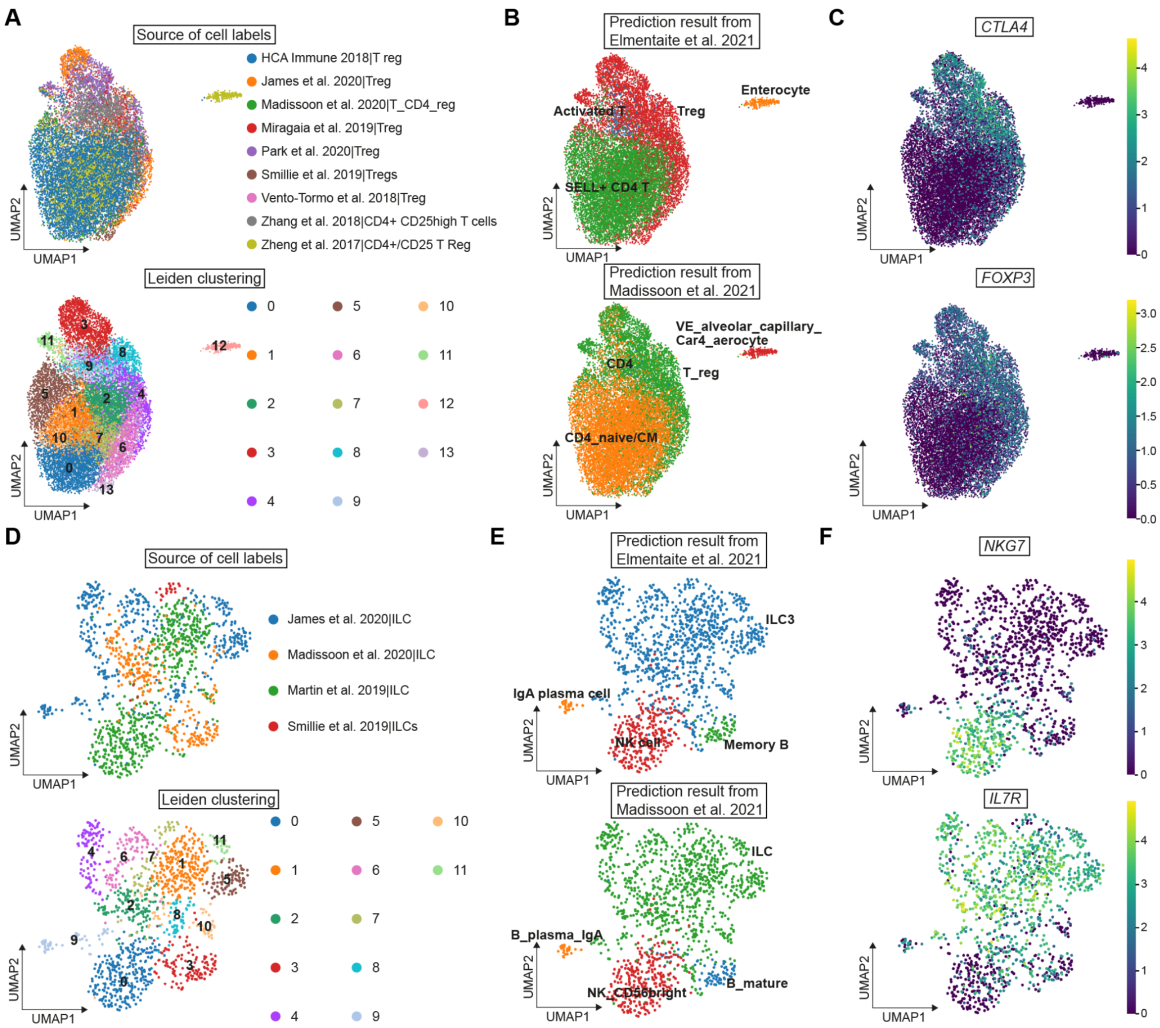

Fig. S5. Two examples of cell type label harmonization through correcting labels of cells that are misclassified by original publications. (**A**) UMAP visualizations of all regulatory T cells in CellTypist training datasets with information of cell sources (datasets followed by original cell type labels, upper) and unsupervised clustering (Leiden clustering based on the neighbourhood graph constructed using scVI-derived latent space, bottom). (**B**) UMAP visualizations of the transferred cell type labels from Elmentaite et al., 2021 (*27*) (upper) and Madissoon et al., 2021 (*28*) (bottom) by training the CellTypist models on the two datasets, respectively. Clusters 0, 1, 2, 5, 7 and 10 are consistently predicted into naive central memory CD4 T cells instead of regulatory T cells, and thus can be renamed and relabelled. (**C**) Expression of regulatory T cell markers *CTLA4* (upper) and *FOXP3* (bottom) overlaid onto the UMAP representations, supporting the exclusion of clusters 0, 1, 2, 5, 7 and 10 from the

regulatory T cell category. (**D** to **F**) As with (A), (B), and (C), but for innate lymphoid cells (ILCs). Cells from cluster 0 are relabelled as NK cells after the correction process.

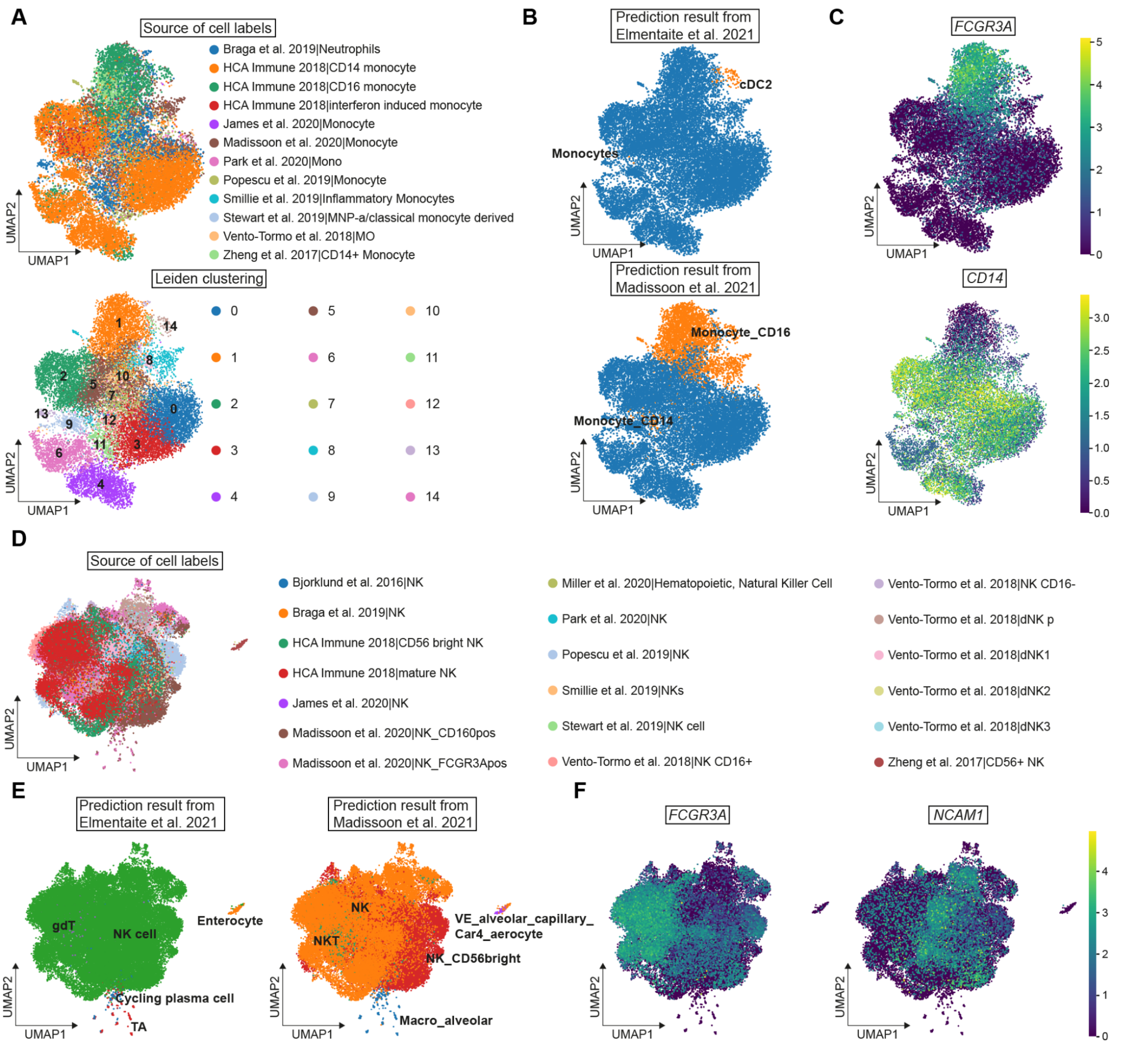

Fig. S6. Two examples of cell type label harmonization through subdividing cell types into well-recognized subtypes. (**A**) UMAP visualizations of all monocytes in CellTypist training datasets with information of cell sources (datasets followed by original cell type labels, upper) and unsupervised clustering (Leiden clustering based on the neighbourhood graph constructed using scVI-derived latent space, bottom). (**B**) UMAP visualizations of the transferred cell type labels from Elmentaite et al., 2021 (*27*) (upper) and Madissoon et al., 2021 (*28*) (bottom) by training the CellTypist models on the two datasets, respectively. Clusters 1 and 8 are predicted into non-classical (CD16+) monocytes, and most of the remaining cells are predicted into classical (CD14+) monocytes. (**C**) Expression of non-classical monocyte marker *FCGR3A* (upper) and classical monocyte marker *CD14* (bottom) overlaid onto the UMAP representations, supporting the subdivision of monocytes into the two subtypes. (**D** to **F**) As with (A),

(B), and (C), but for natural killer (NK) cells. A subtype division is found between CD16+ and CD16- (CD56+) NK cells.

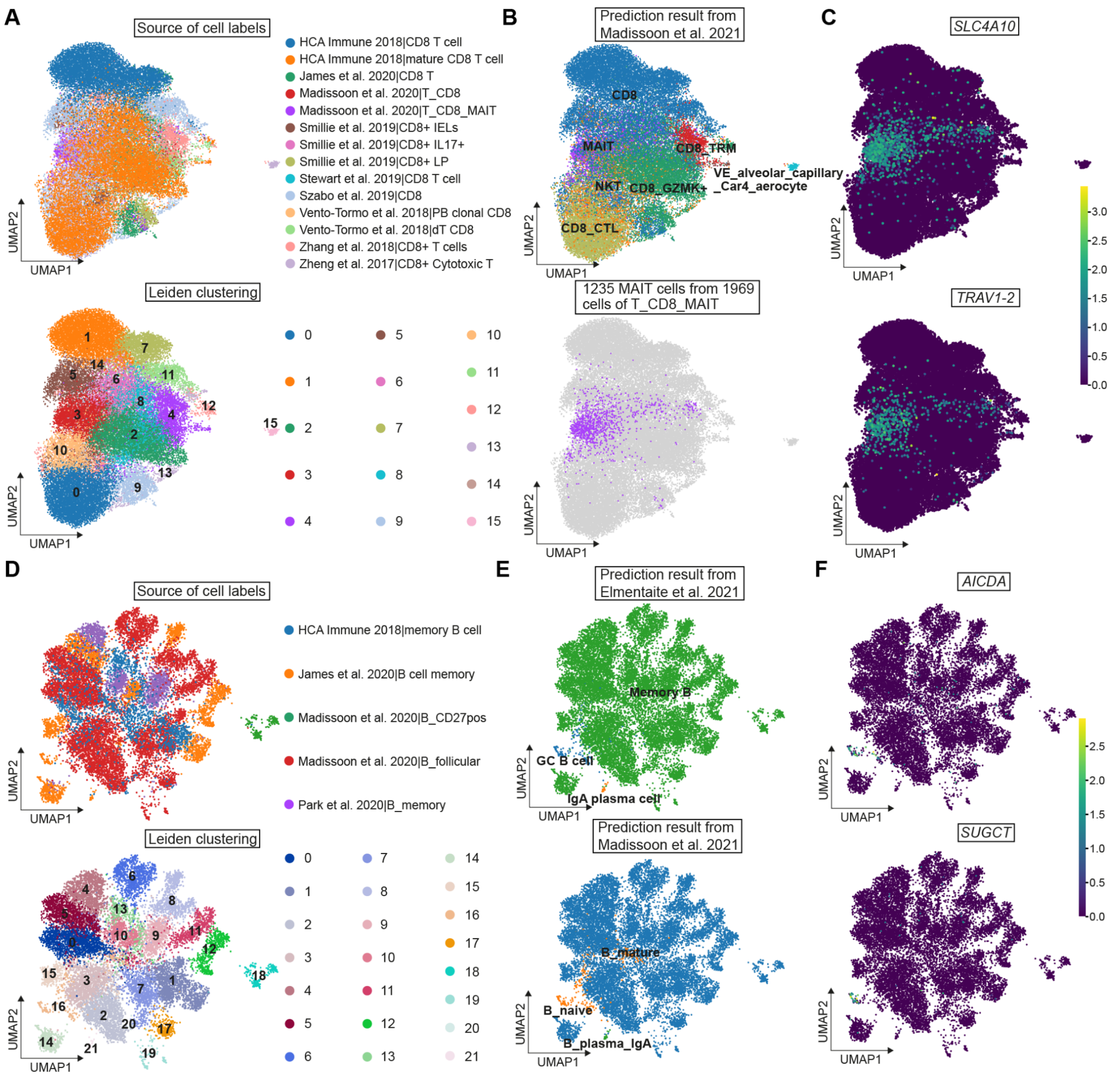

Fig. S7. **Two examples of cell type label harmonization through identifying hidden populations among given cell types.** (**A**) UMAP visualizations of all cytotoxic T cells in CellTypist training datasets with information of cell sources (datasets followed by original cell type labels, upper) and unsupervised clustering (Leiden clustering based on the neighbourhood graph constructed using scVI-derived latent space, bottom). (**B**) UMAP visualizations of the transferred cell type labels from Madissoon et al., 2021 (*28*) by training the CellTypist model on this dataset. A group of 1,235 cells are projected as mucosal-associated invariant T (MAIT) cells out of 1,969 cells with the original cell type label 'T_CD8_MAIT'. (**C**) Expression of MAIT cell markers *SLC4A10* (upper) and *TRAV1-2* (bottom) overlaid onto the UMAP representations, supporting the identity of these cells as MAIT cells. (**D** to **F**) As with (A), (B), and (C), but for identifying germinal center B cells from memory B cells. Cells from cluster 16

are relabelled as germinal center B cells. Note that due to the lack of germinal center B cells in Madissoon et al., 2021, cells of cluster 16 are predicted as naive B cells instead.

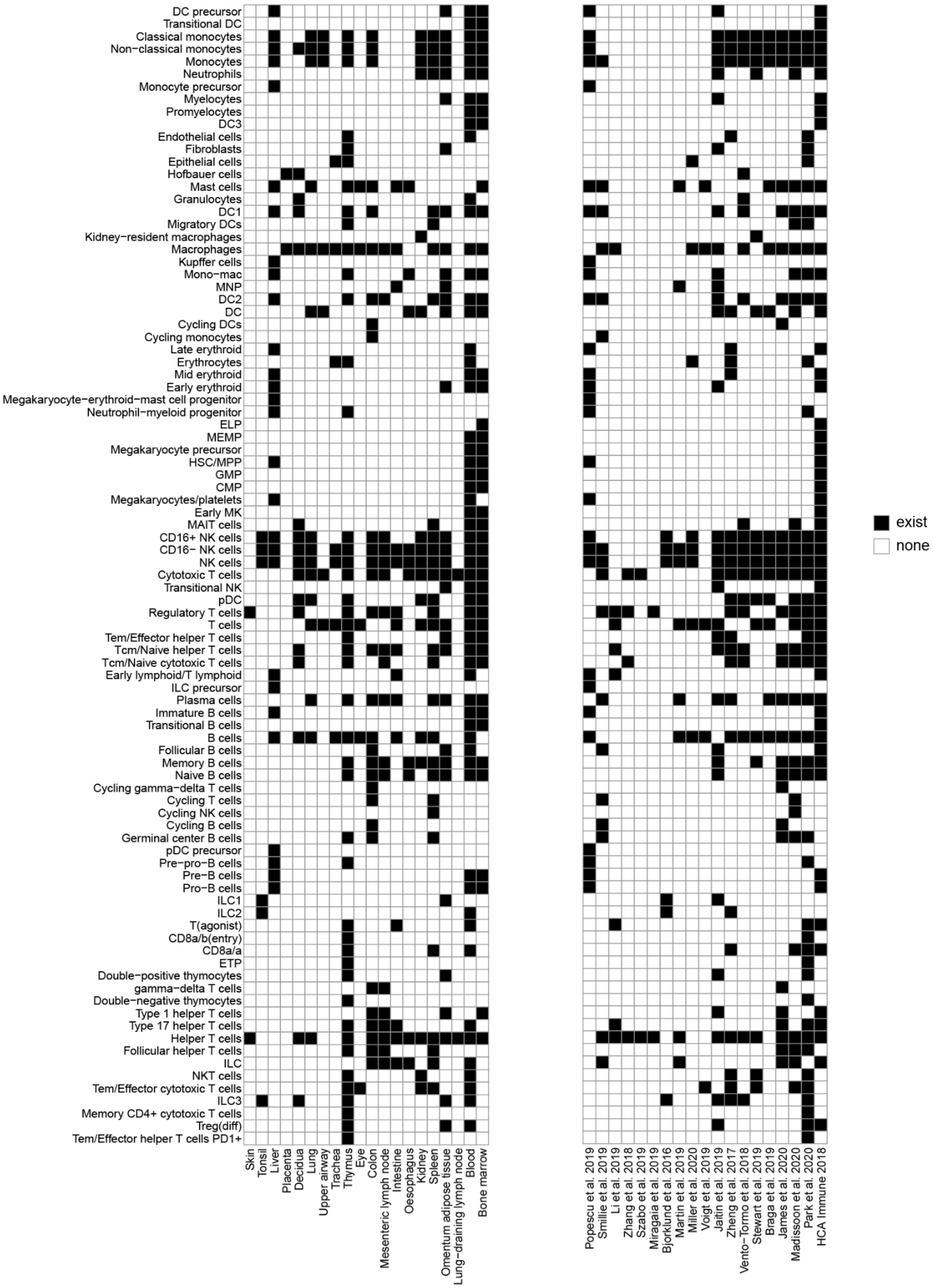

Fig. S8. **Summary of cross-dataset and cross-tissue harmonized cell types.** Binary heat maps showing the distributions of harmonized cell types across tissues (left) and datasets (right). Black grids denote the presence of cell types.

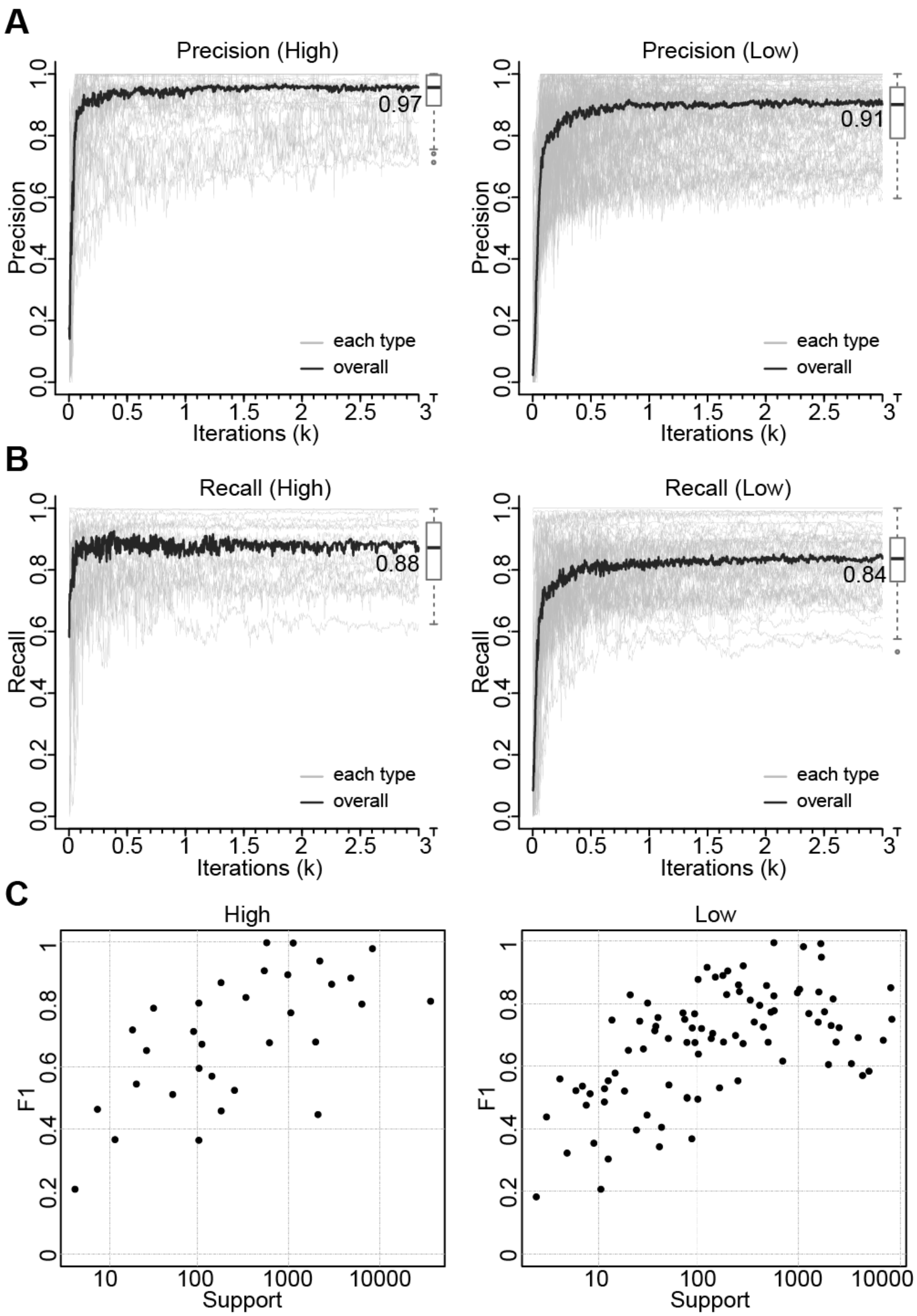

Fig. S9. **Building a human immune reference to predict immune cell identities.** (**A** and **B**) Performance curves showing the precision (A) and recall (B) scores at each iteration of training using mini-batch stochastic gradient descent for high- and

low-hierarchy CellTypist models, respectively. The black curves represent the median scores averaged across the individual scores of all predicted cell types (grey curves). (**C**) F1-score for each tested high-hierarchy (left) or low-hierarchy (right) cell type as a function of its representation in the compiled human immune datasets (corresponding to 10% of the total cells).

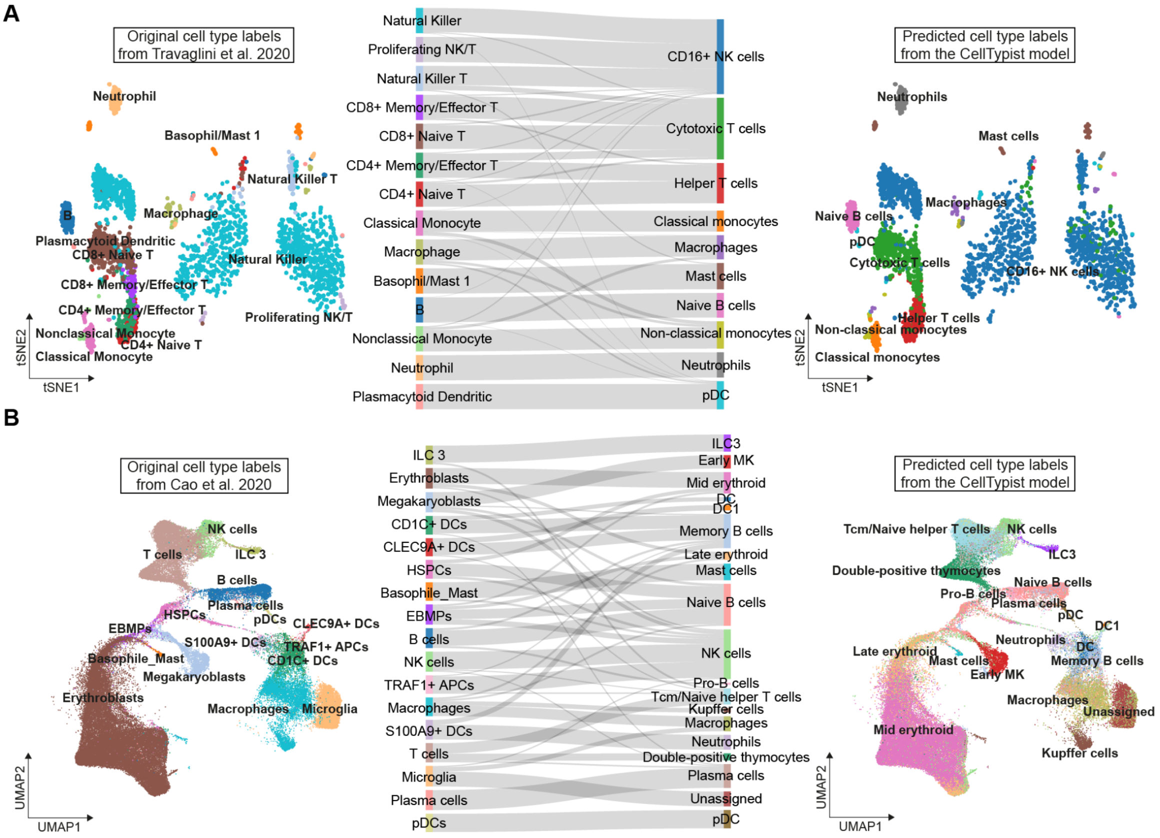

Fig. S10. Performance of CellTypist models on datasets with different levels of gene expression sparseness. (**A**) UMAP representations of a Smart-seq2 immune dataset (*91*) colored by original cell types (left), as well as colored by predicted cell types after over-clustering and majority-voting using the CellTypist pipeline. Sankey plot in the middle shows the correspondence between the two sets of cell type labels. (**B**) As with (A), but for a sci-RNA-seq3 blood and immune dataset (*92*).

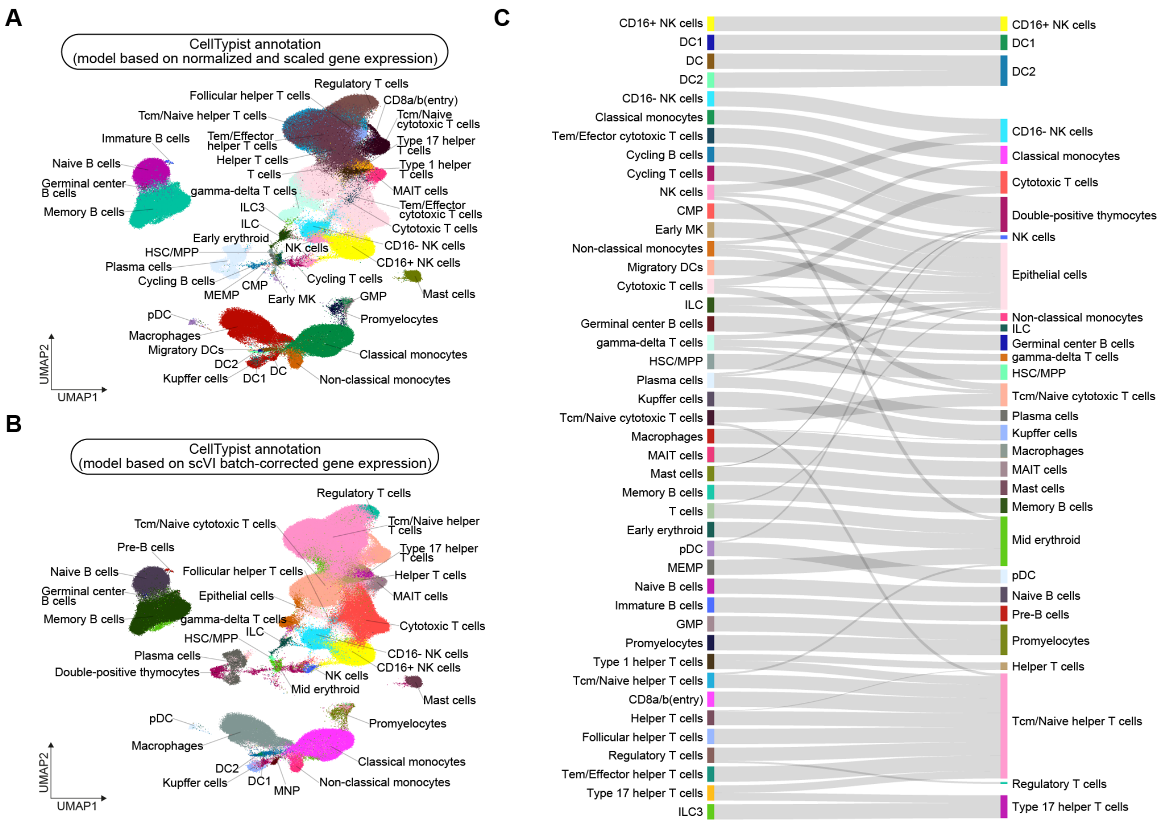

Fig. S11. Comparisons of CellTypist model performance with and without batch correction. (**A**) UMAP visualization of the immune cell compartment colored by cell types predicted using the CellTypist pipeline based on normalized and scaled gene expression matrix. (**B**) As with (A), but colored by cell types predicted using the CellTypist pipeline based on scVI batch-corrected gene expression matrix. (**C**) Sankey plot showing the correspondence between cell types in (A) and (B).

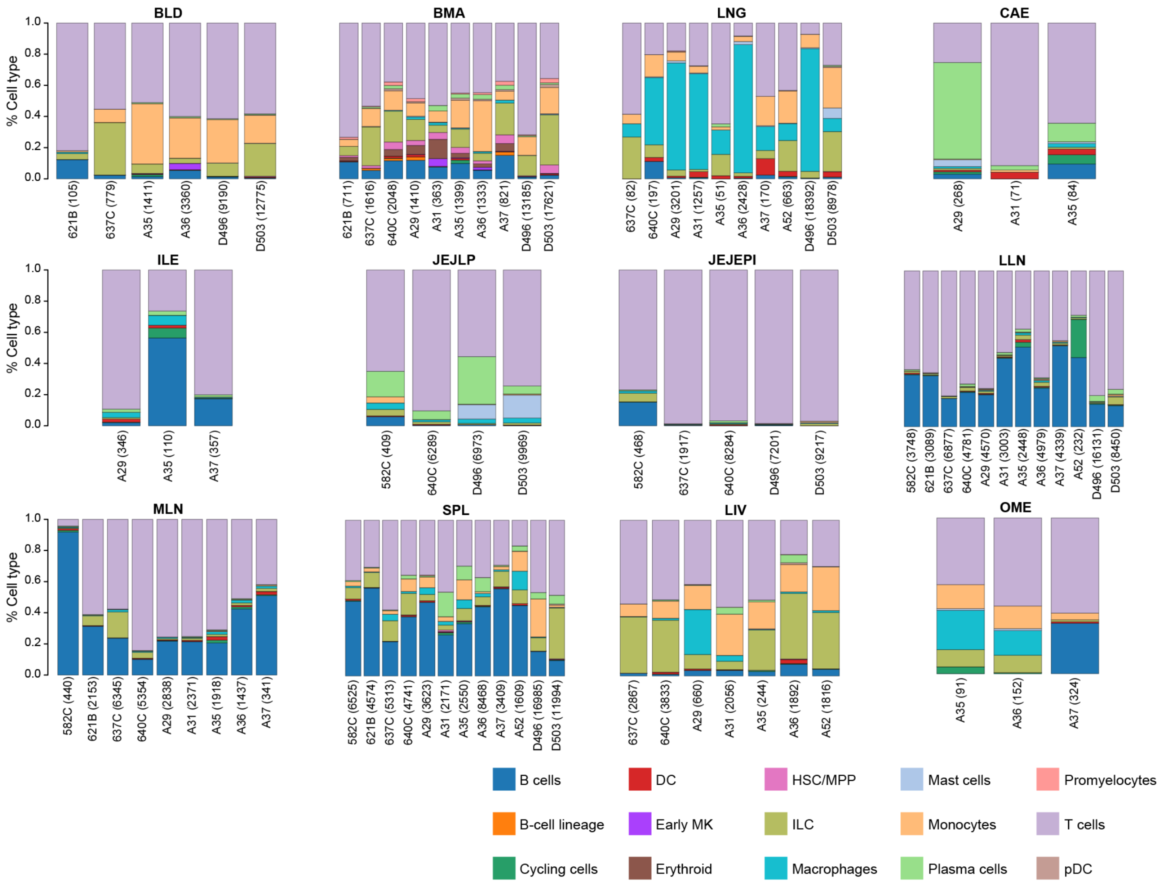

Fig. S12. Overview of the donors and cell types contributing to each tissue. Stacked bar plots demonstrating the cell type compositions across donors in each tissue/organ. Only tissues with cell numbers of greater than 50 in at least two donors are shown.

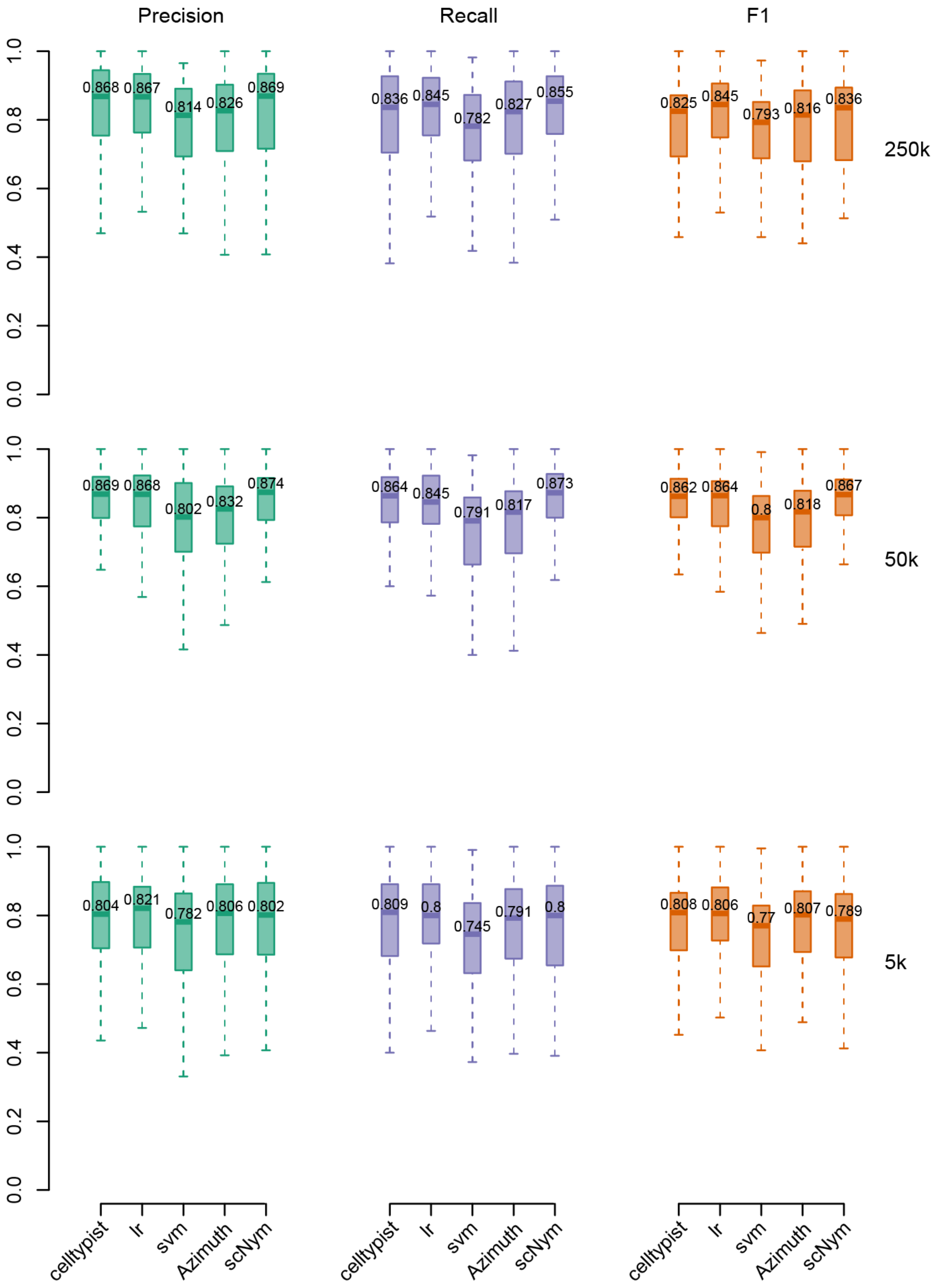

Fig. S13. Benchmarking of CellTypist accuracy with other methods. Box plots showing the prediction precision (left), recall (center) and F1 score (right) for the training dataset with 5,000 (lower), 50,000 (middle), and 250,000 (upper) cells,

respectively. Five methods are assessed and the median value of these metrics across individual cell types is shown for each method.

A

	CellTypist	lr	svm	Azimuth	scNym
**Training**	*celltypist.train*	*LogisticRegression.fit*	*LinearSVC.fit*	*FindTransferAnchors*	*scnym_api(task='train')*
**Prediction**	*celltypist.annotate*	*LogisticRegression.predict*	*LinearSVC.predict*	*TransferData*	*scnym_api(task='predict')*
**User**	*celltypist.annotate*	*LogisticRegression.predict*	*LinearSVC.predict*	*FindTransferAnchors*<br>*TransferData*	*scnym_api(task='train')*<br>*scnym_api(task='predict')*

B

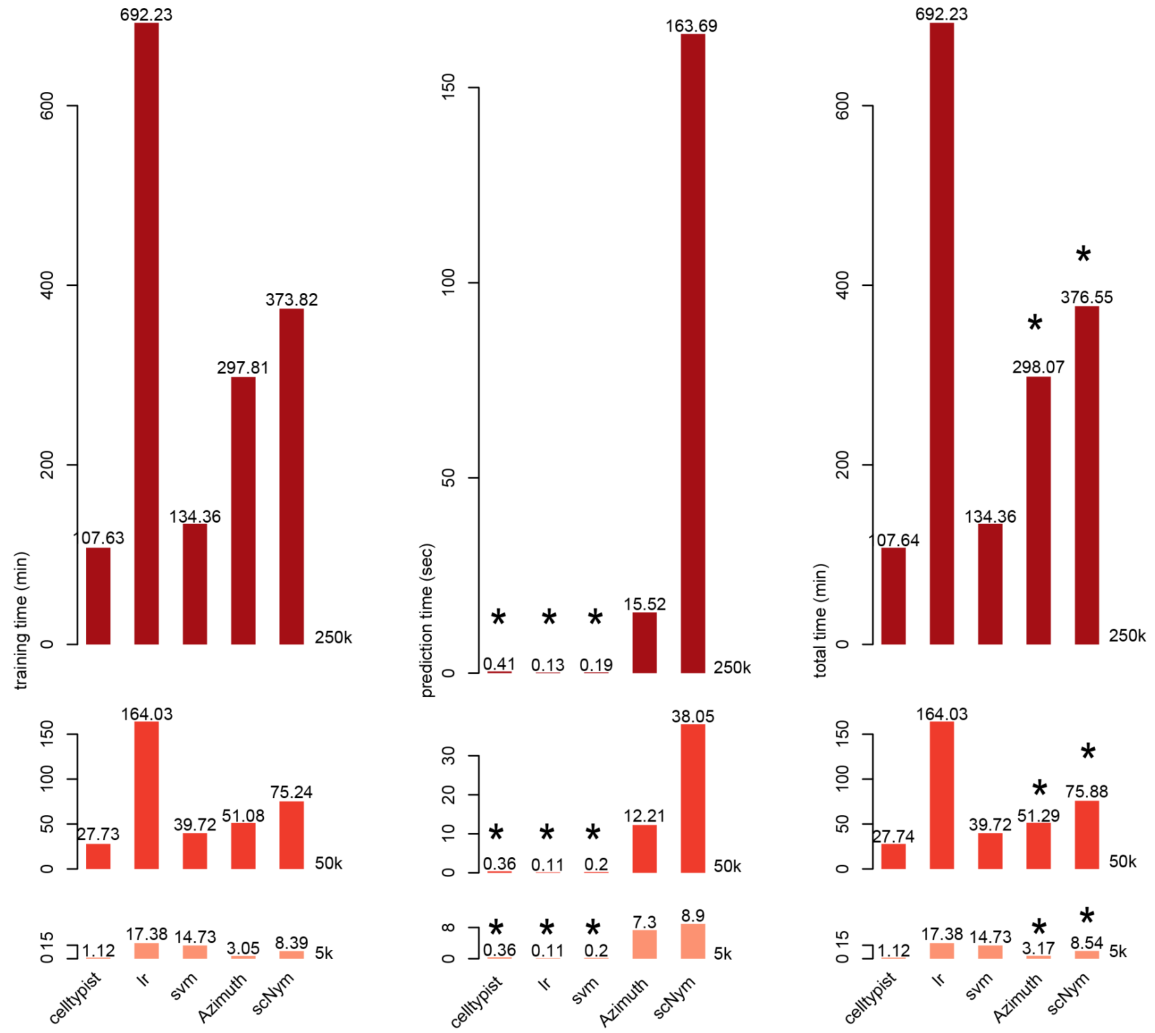

Fig. S14. Benchmarking of CellTypist time complexity with other methods. (**A**) Table summarizing the split of different label transfer methods into the training and prediction steps. The "user" row shows the step/steps a user needs to get their prediction results after inputting the query data. (**B**) Bar plots showing the training time in minutes (left), prediction time in seconds (center) and total time in minutes (right) for the training dataset with 5,000 (lower), 50,000 (middle), and 250,000 (upper) cells, respectively. Five methods are assessed and the time is shown for each combination of training data and methods. Asterisks mark the user time for different methods.

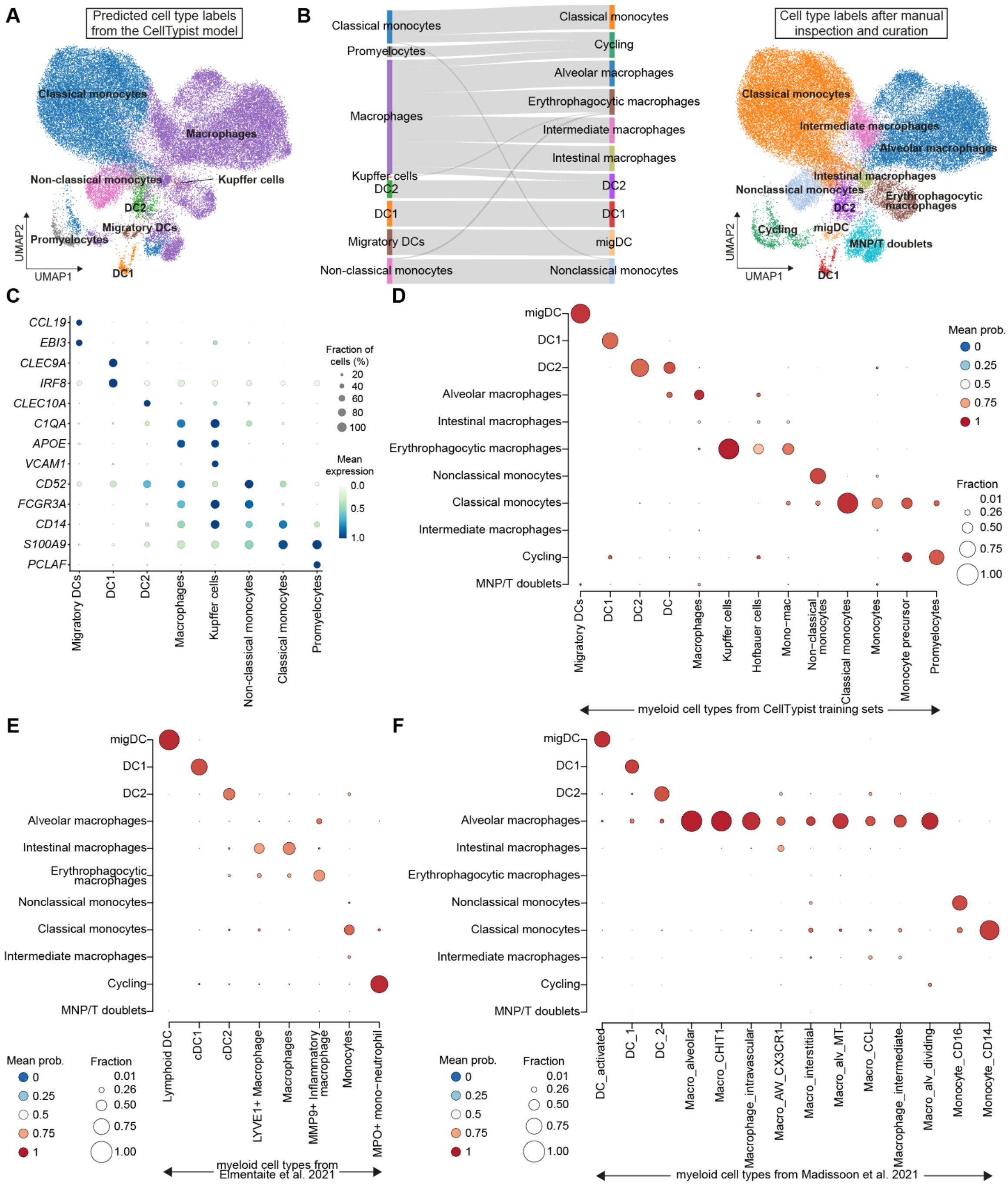

Fig. S15. CellTypist prediction of the myeloid compartment and cell type cross-validation with external datasets. (**A**) UMAP visualization of the myeloid compartment colored by predicted cell types from CellTypist. (**B**) As in (A), but colored by manually annotated cell types after curation of the CellTypist prediction result (right). Sankey plot on the left shows the correspondence between the two sets of cell type labels. MNP/T doublets are not shown in the Sankey plot to avoid strong noise signals from this cluster for the sake of visual inspection. (**C**) Dot plot displaying the expression of CellTypist-derived marker genes for the predicted myeloid populations.

Color gradient represents maximum-normalized mean expression of cells expressing the marker genes, and size represents the percentage of cells expressing these genes. (**D**) Dot plot showing the cell type cross-validation by transferring cell type labels from our resource (row) to cells from the CellTypist training datasets (column). For each column (each cell type from the CellTypist training sets), size of a dot denotes the proportion of cells assigned to a given cell type of the resource and color denotes the average probabilities calculated from CellTypist. (**E** and **F**) As with (D), but for cross-validation with the gut myeloid populations from Elmentaite et al. 2021 (*27*) and with the lung myeloid populations from Madissoon et al. 2021 (*28*).

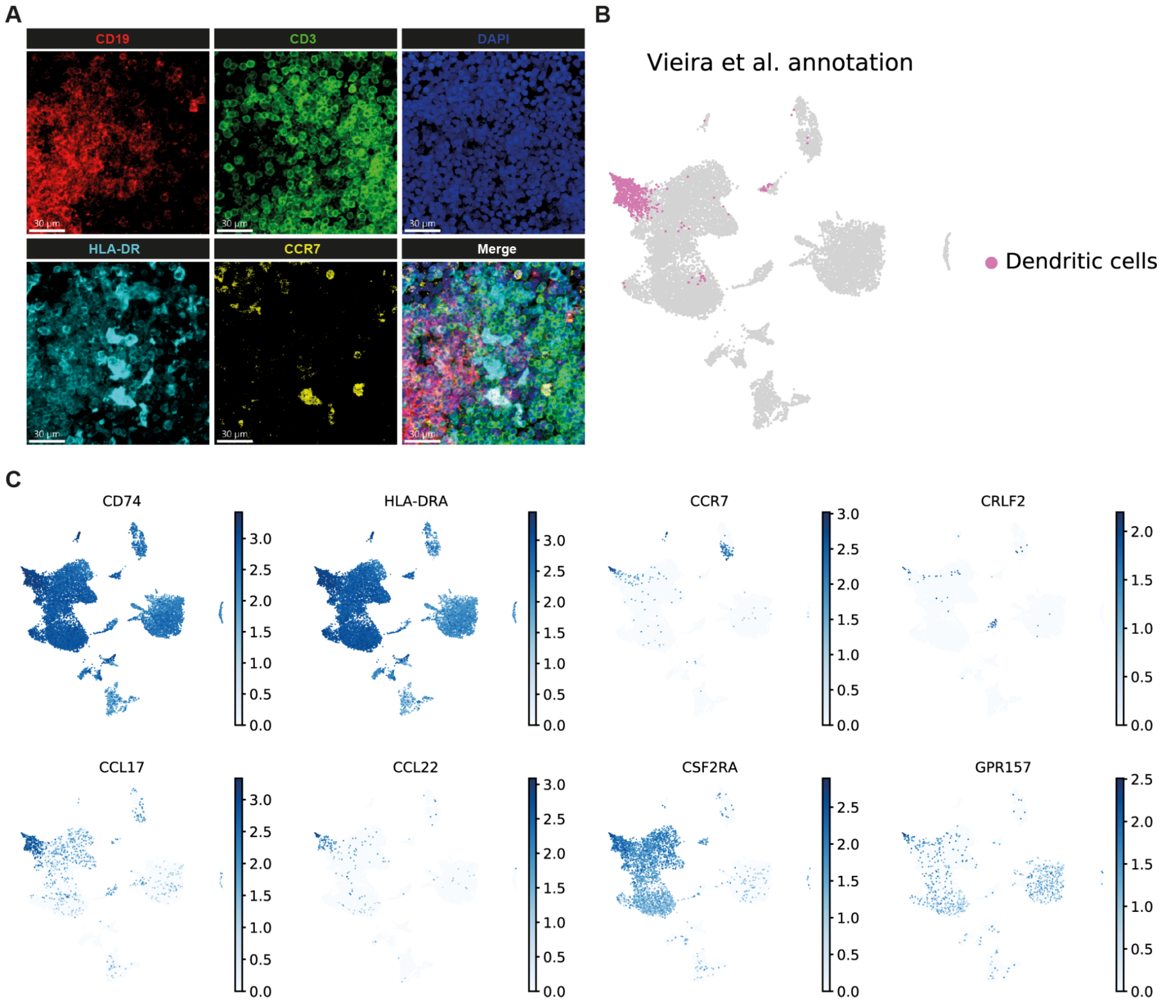

Fig. S16. Migratory dendritic cells in the lymph nodes and lung. (**A**) Immunofluorescence validation of the CCR7+ migratory DCs in the thoracic lymph nodes. (**B**) UMAP showing the distribution of lung dendritic cells in the scRNA-seq dataset from Vieira et al., 2019 (*45*). (**C**) UMAP plots of cells from Vieira et al., 2019(*45*) as in (B), overlaid by expression of genes highly expressed by the migratory DCs we identified in our data.

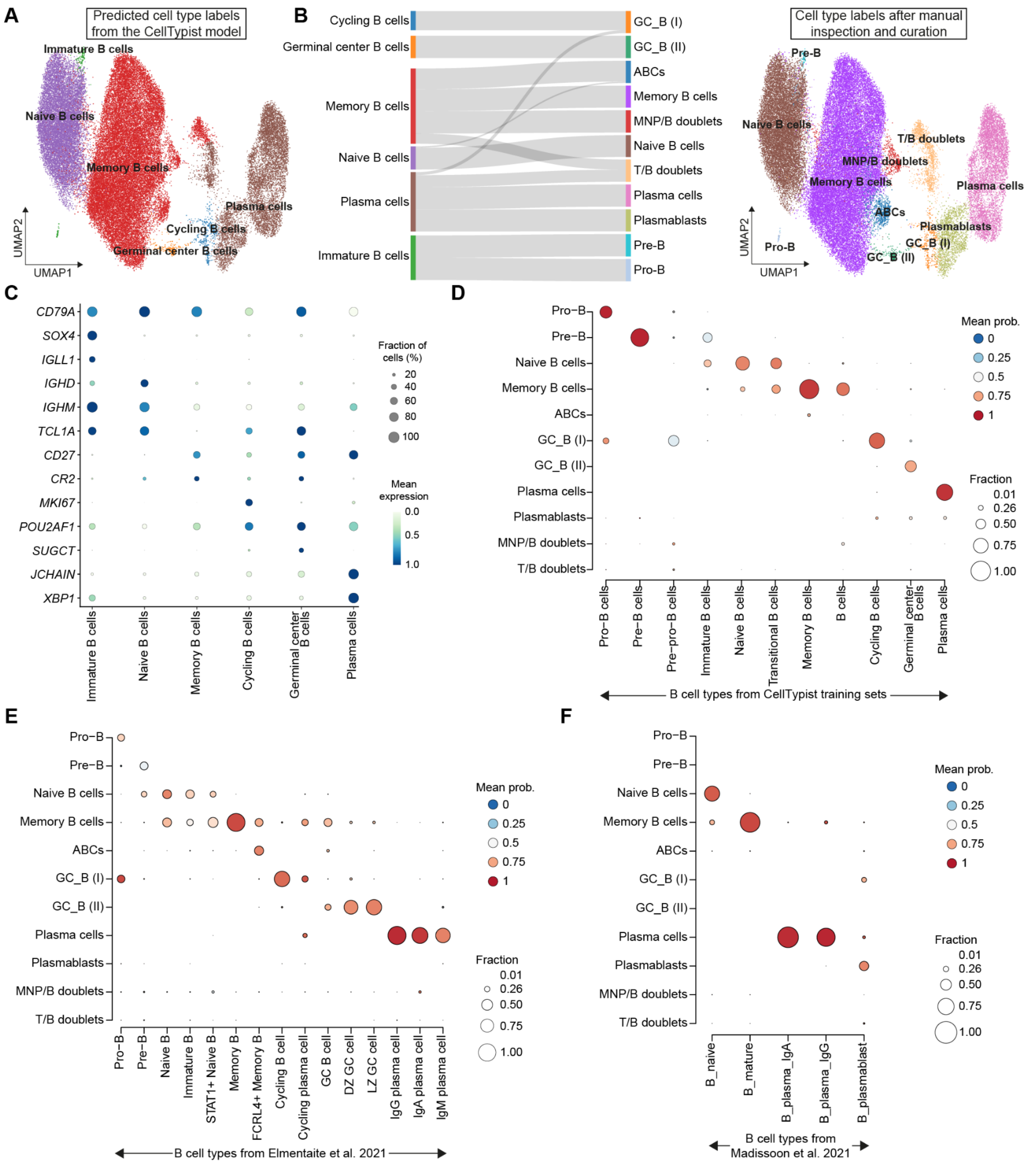

Fig. S17. CellTypist prediction of the B cell compartment and cell type cross-validation with external datasets. (**A**) UMAP visualization of the B cell compartment colored by predicted cell types from CellTypist. (**B**) As in (A), but colored by manually annotated cell types after curation of the CellTypist prediction result (right). Sankey plot on the left shows the correspondence between the two sets of cell type labels. (**C**) Dot plot displaying the expression of CellTypist-derived marker genes for the predicted B cell populations. Color gradient represents maximum-normalized mean expression of cells expressing the marker genes, and size represents the percentage of cells expressing these genes. (**D**) Dot plot showing the cell type

cross-validation by transferring cell type labels from our resource (row) to cells from the CellTypist training datasets (column). For each column (each cell type from the CellTypist training sets), size of a dot denotes the proportion of cells assigned to a given cell type of the resource and color denotes the average probabilities calculated from CellTypist. (**E** and **F**) As with (D), but for cross-validation with the gut B cell populations from Elmentaite et al. 2021 (*27*) and with the lung B cell populations from Madissoon et al. 2021 (*28*).

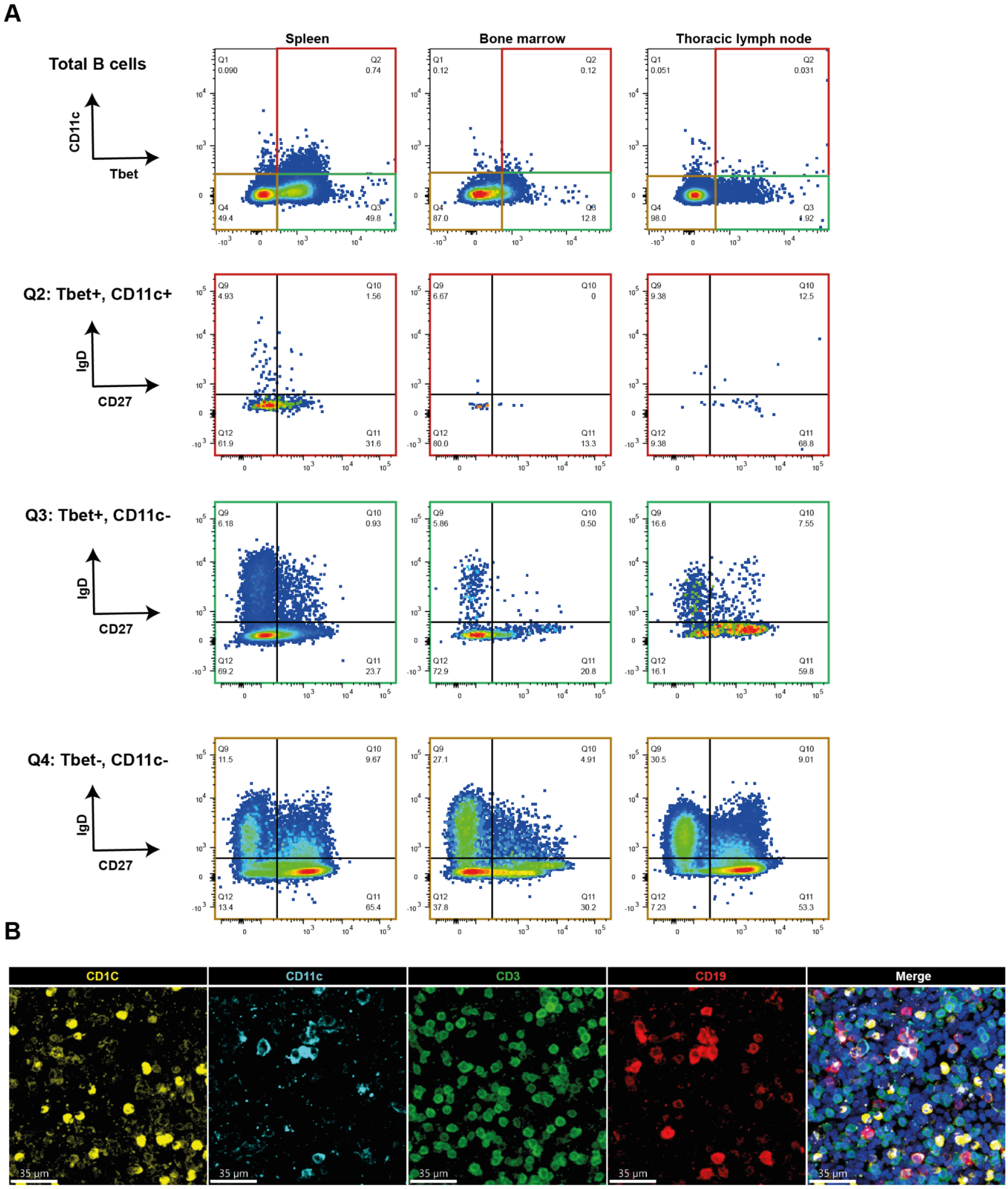

Fig. S18. Validation of the *ITGAX*+ memory B cells. (**A**) Flow cytometry analysis of memory B cells expressing CD11c (encoded by *ITGAX*) and T-bet (encoded by *TBX21*). (**B**) Immunofluorescence of spleen tissue showing colocalization of CD11c with CD19 (marking age-associated B cells).

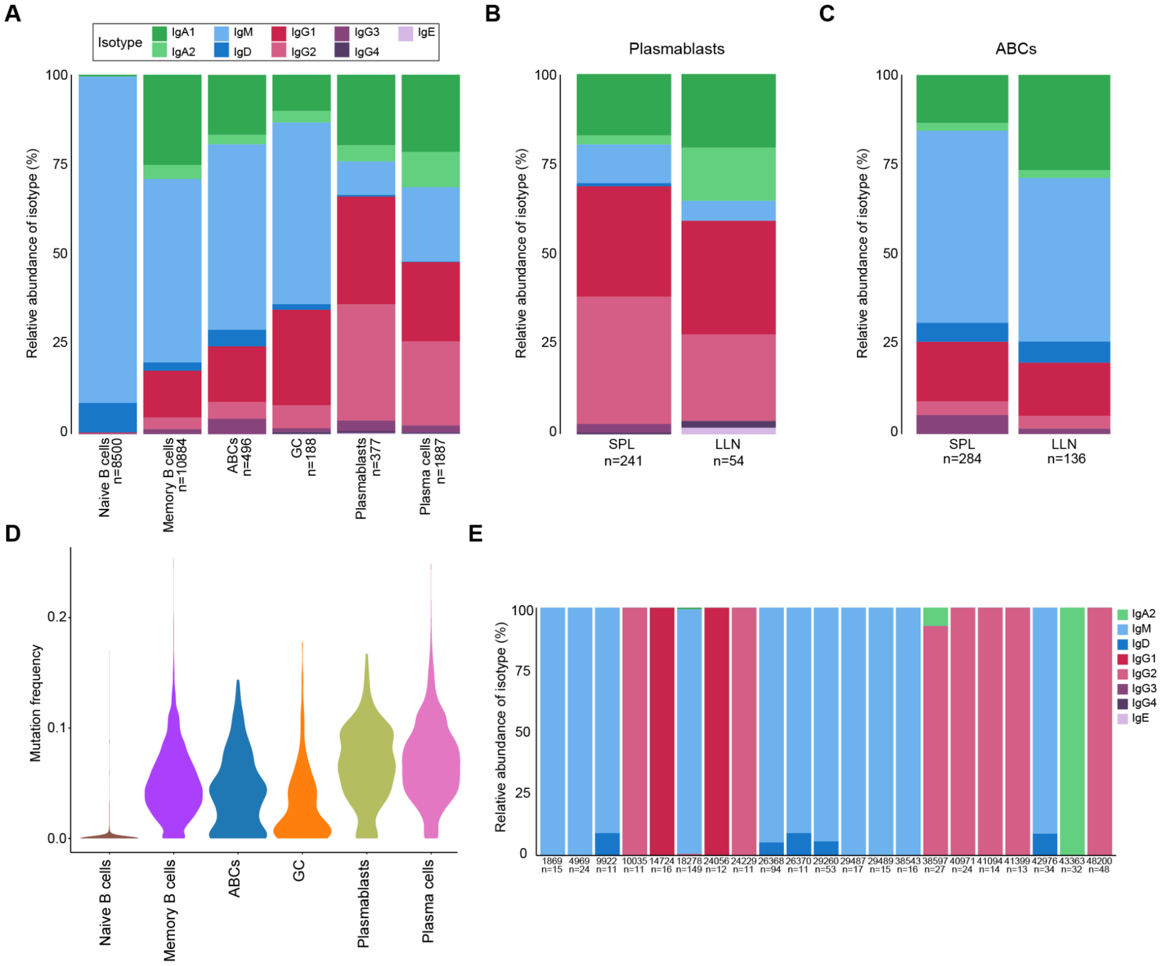

Fig. S19. Assessment of isotype features and somatic hypermutation levels in the B cell compartment. (**A** to **C**) Stacked bar plot showing the isotype distribution across B cell subsets (A), within the plasmablasts across tissues (B), and within the age-associated memory B cells (ABCs) across tissues (C). (**D**) Violin plot showing hypermutation frequency across B cell subsets. (**E**) Stacked bar plot of the isotype distribution across 21 expanded clonotypes.

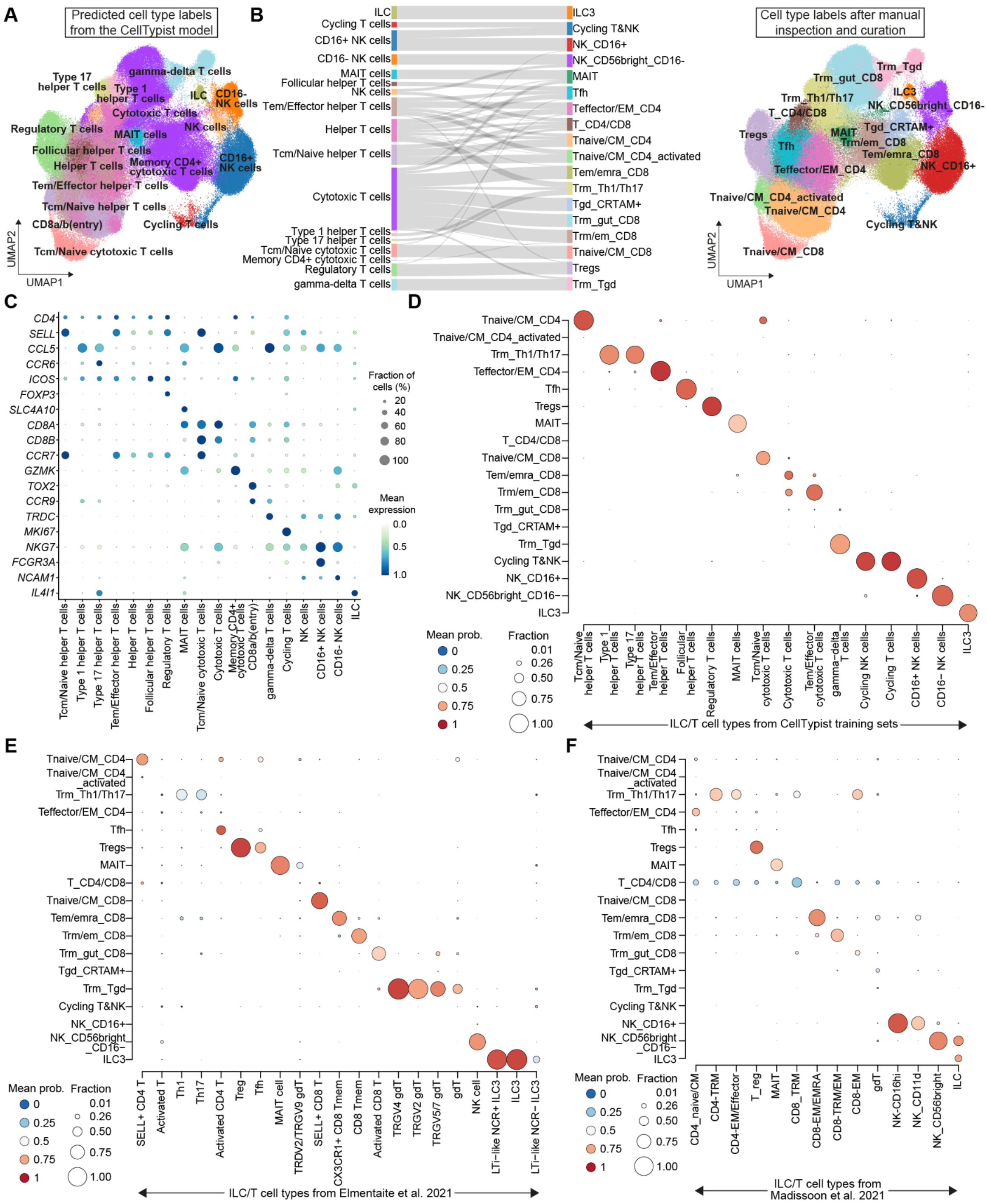

Fig. S20. CellTypist prediction of the T cell compartment and cell type cross-validation with external datasets. (**A**) UMAP visualization of the T cell compartment colored by predicted cell types from CellTypist. (**B**) As in (A), but colored by manually annotated cell types after curation of the CellTypist prediction result (right). Sankey plot on the left shows the correspondence between the two sets of cell type labels. CD8a/b(entry) with a weak strength of link (<0.01) is not shown in the Sankey plot. (**C**) Dot plot displaying the expression of CellTypist-derived marker genes

for the predicted T cell populations. Color gradient represents maximum-normalized mean expression of cells expressing the marker genes, and size represents the percentage of cells expressing these genes. (**D**) Dot plot showing the cell type cross-validation by transferring cell type labels from our resource (row) to cells from the CellTypist training datasets (column). For each column (each cell type from the CellTypist training sets), size of a dot denotes the proportion of cells assigned to a given cell type of the resource and color denotes the average probabilities calculated from CellTypist. (**E** and **F**) As with (D), but for cross-validation with the gut T cell populations from Elmentaite et al. 2021(*27*) and with the lung T cell populations from Madissoon et al. 2021(*28*).

Donor 1

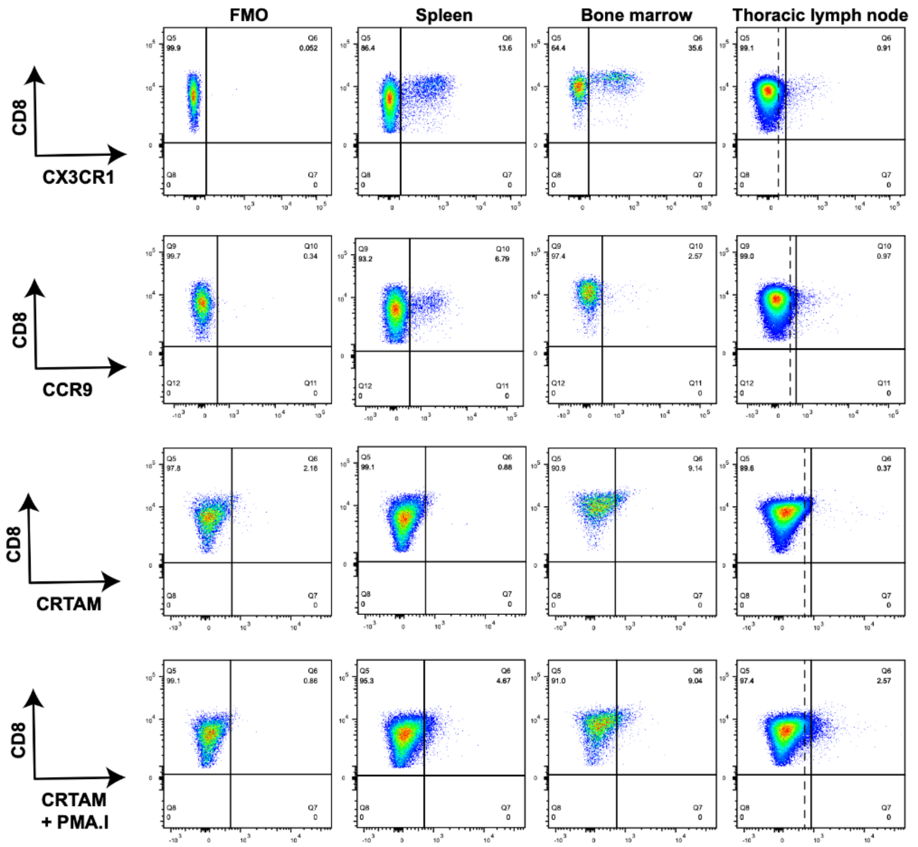

Donor 2

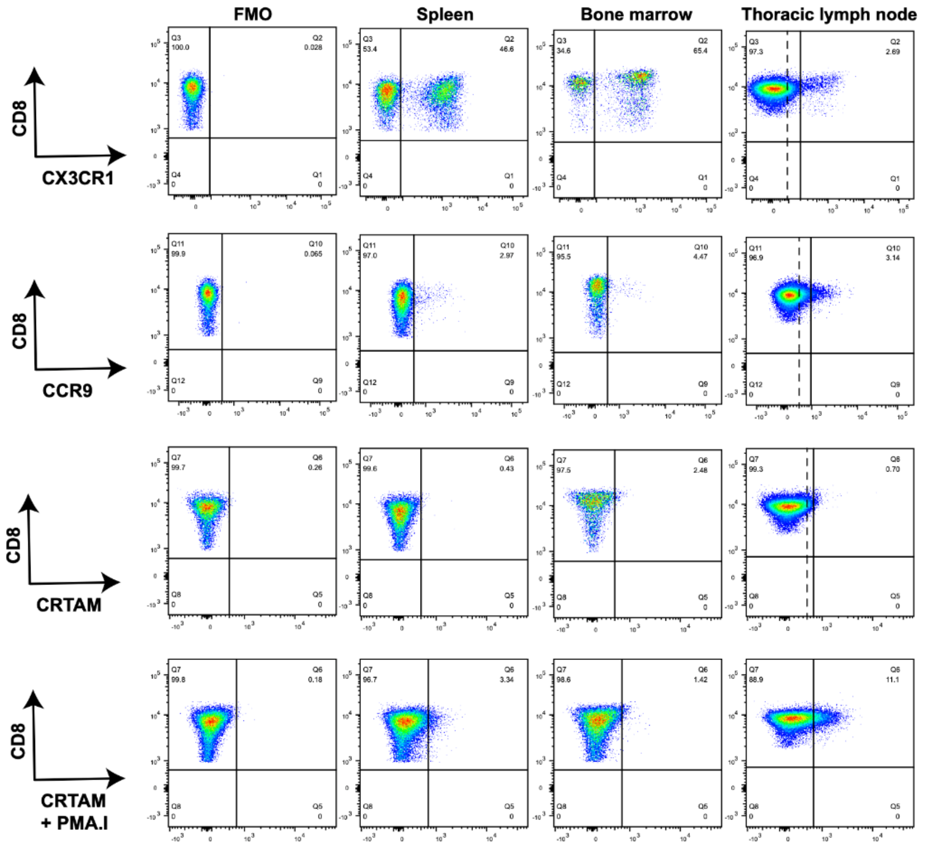

Fig. S21. Validation of CD8+ CX3CR1+, CD8+ CCR9+ and CD8+ CRTAM+ cells in mononuclear cells (MNCs) from spleen, bone marrow and thoracic lymph nodes of two donors. Spleen MNCs were used to generate FMO stains due to cell availability. CRTAM staining was performed +/- 2 hour stimulation with PMA/Ionomycin. The position of the quadrant gate was manually adjusted for autofluorescence in thoracic lymph node samples.

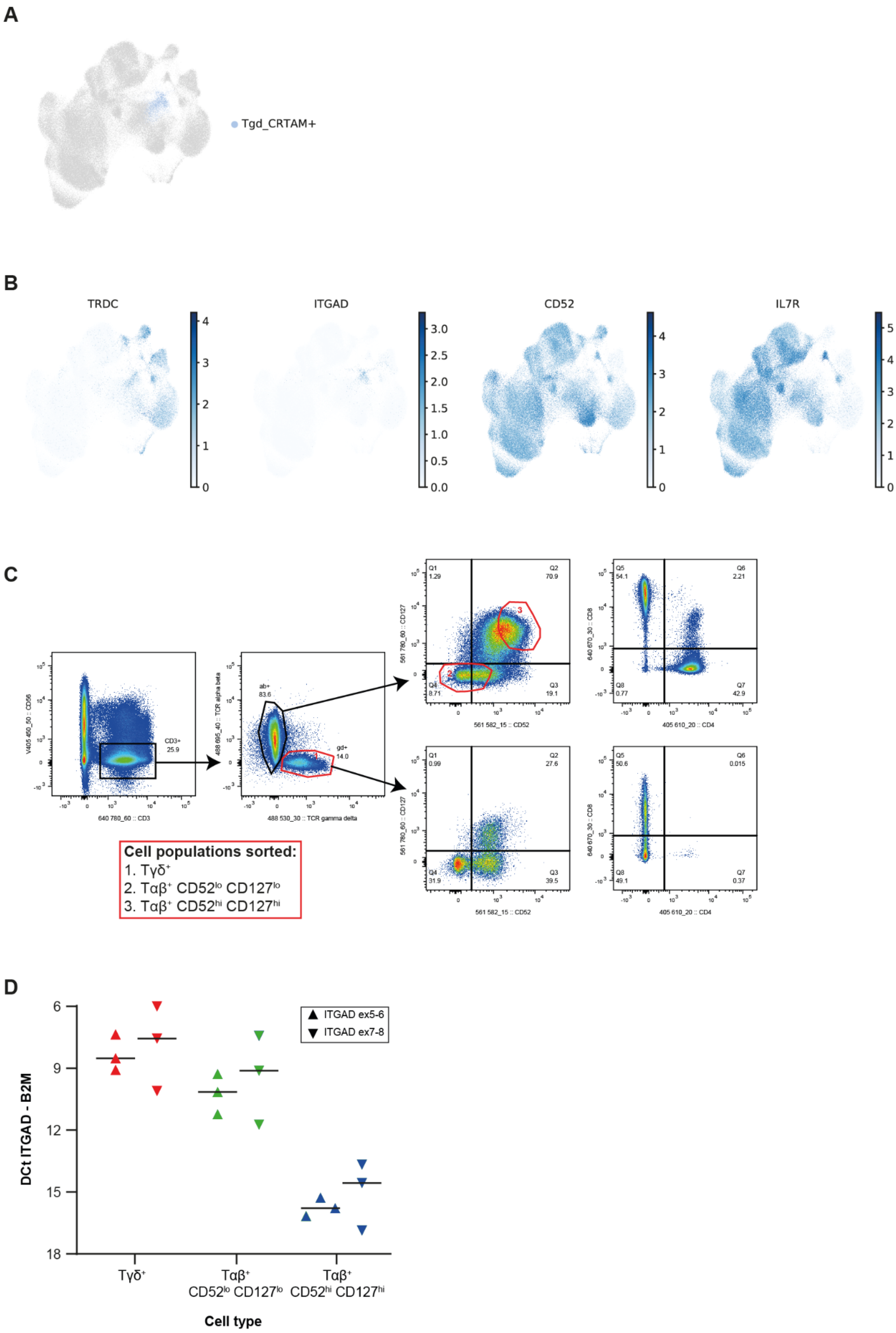

Fig. S22. Validation of ITGAD expressing γδT cells. (**A**) The location of the Tgd_CRTAM subset is shown on the UMAP visualization of T cells and ILCs. (**B**)

CD52 and CD127 low expression correlated with ITGAD expression on γδT cells and was used to enrich ITGAD expressing cells as no conjugated antibody was available commercially. (**C**) Tγδ and Tαβ cell populations were sort purified (orange polygon gates) from spleen mononuclear cells (MNCs) and an example of the sort strategy is shown. The right hand plots display CD4 and CD8 expression of the Tαβ and Tγδ cells for the reader's information and were not used as part of the sort strategy. (**D**) qPCR validation of *ITGAD* in T cell populations as measured by two assays to *ITGAD* and normalised to the housekeeping gene *B2M*.

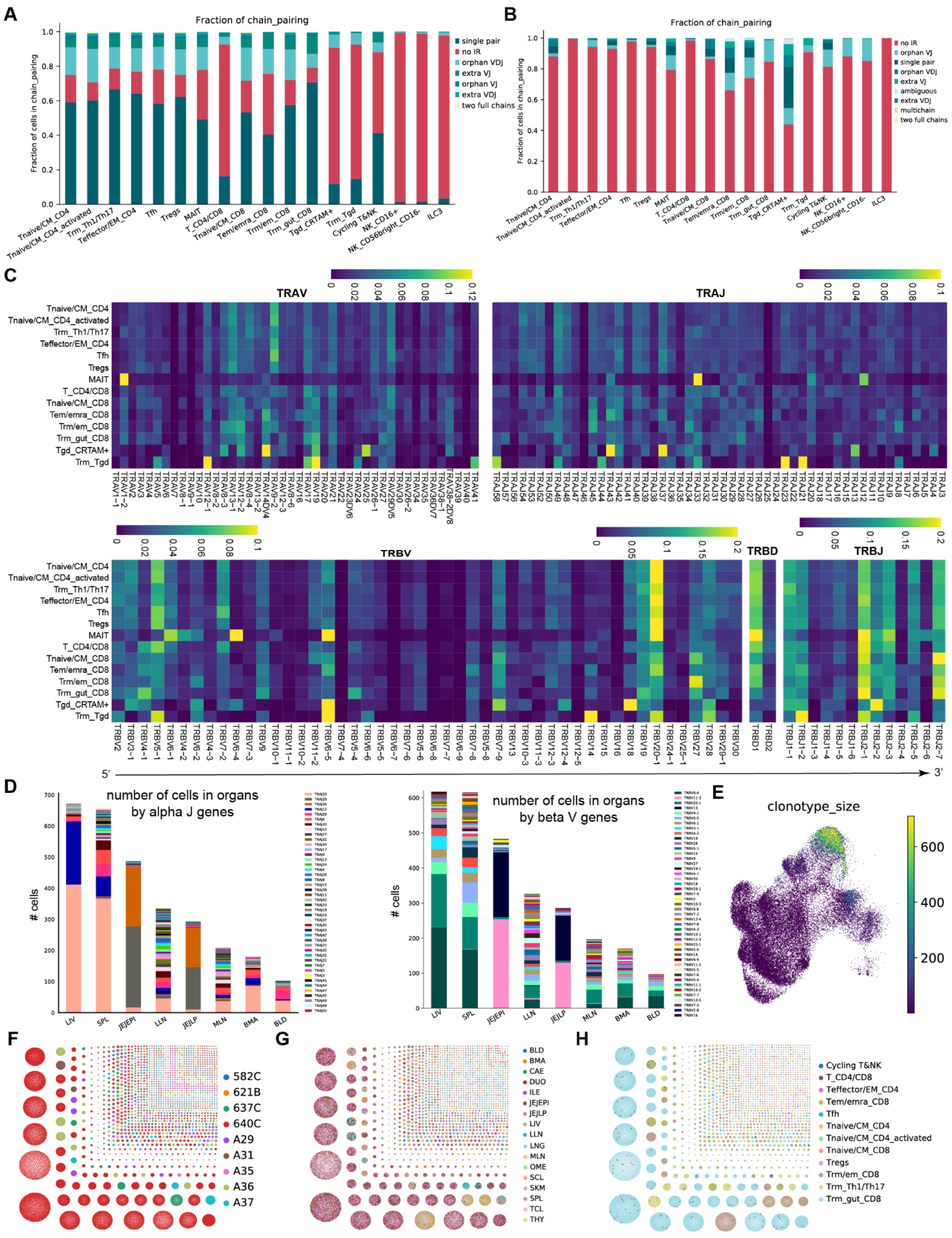

Fig. S23. V(D)J gene usage and clonal architecture of the T cell compartment. (**A**) Proportion of TCRαβ chain pairing across cell types. Only samples that underwent TCRαβ sequencing are shown. (**B**) Proportion of TCRγδ chain pairing across cell types. Only samples that underwent TCRγδ sequencing are shown. (**C**) Heatmap showing relative usage of V(D)J genes across T cell subsets. (**D**) Stacked bar plot showing the

TRAJ and TRBV usage in TRAV1-2+ cells across tissues. **(E)** UMAP showing clonal expansion of the TCRαβ T cells. (**F** to **H**) Clonotype network color coded by donor, tissue and T cell subsets.

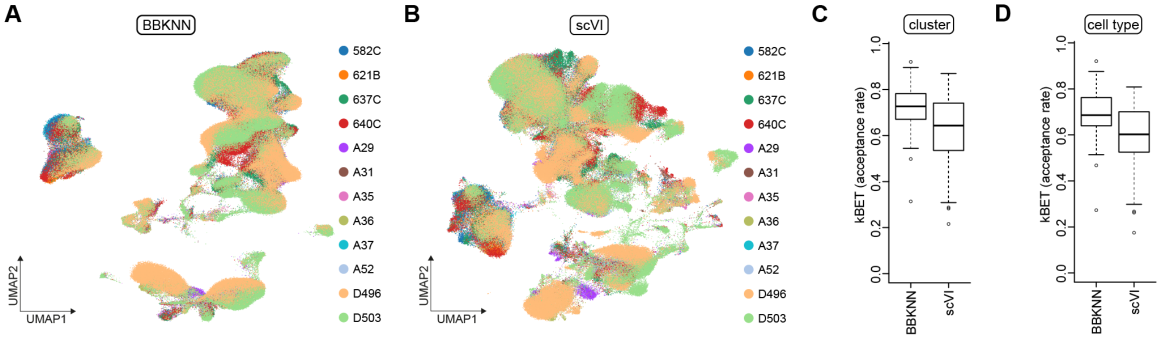

Fig. S24. Comparisons of data integration using BBKNN and scVI. (**A**) UMAP visualization of all immune cells integrated using BBKNN, colored by the donor information. (**B**) As with (A), but integrated using scVI. (**C**) Box plot showing the kBET acceptance rates across clusters derived from the neighborhood graph of BBKNN (left) versus scVI (right). (**D**) As in (C), but across cell types predicted from CellTypist.

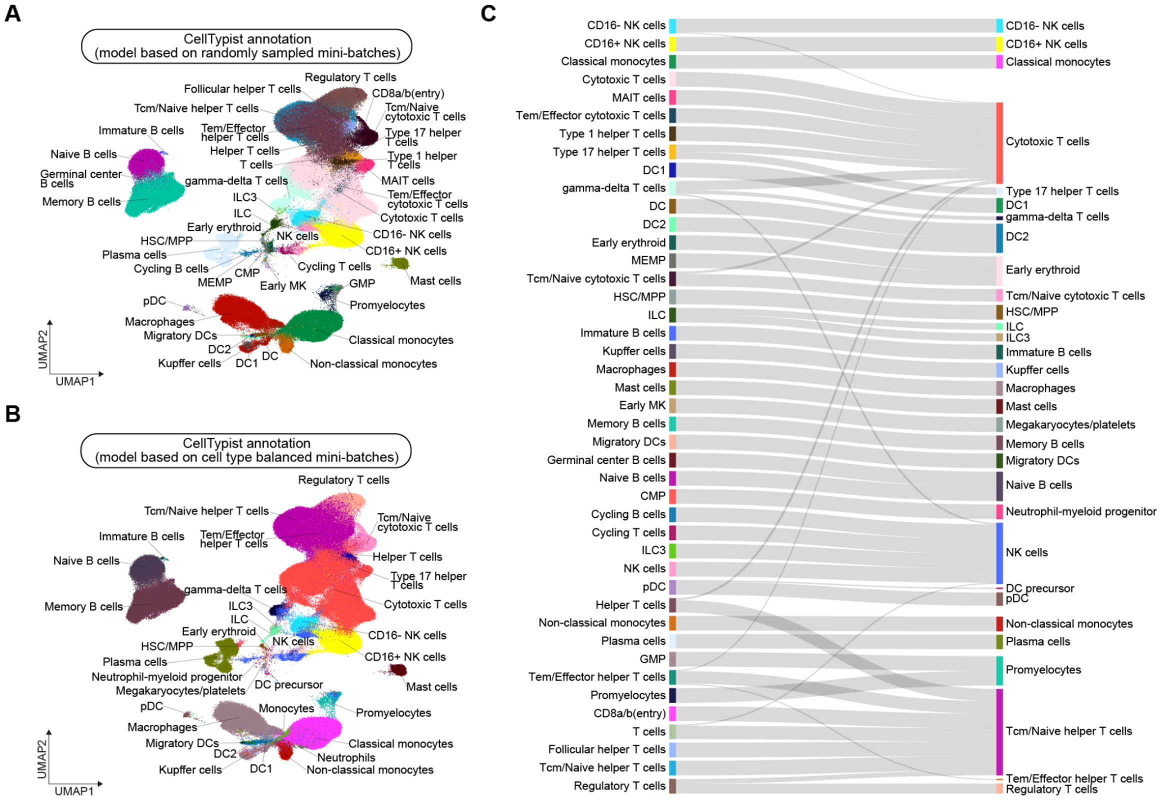

Fig. S25. Comparisons of CellTypist model performance with and without balancing cell type compositions in mini-batches. (**A**) UMAP visualization of the immune cell compartment colored by cell types predicted using the CellTypist pipeline based on randomly sampled mini-batches. (**B**) As with (A), but colored by cell types predicted using the CellTypist pipeline based on cell type-balanced mini-batches. (**C**) Sankey plot showing the correspondence between cell types in (**A**) and (**B**).

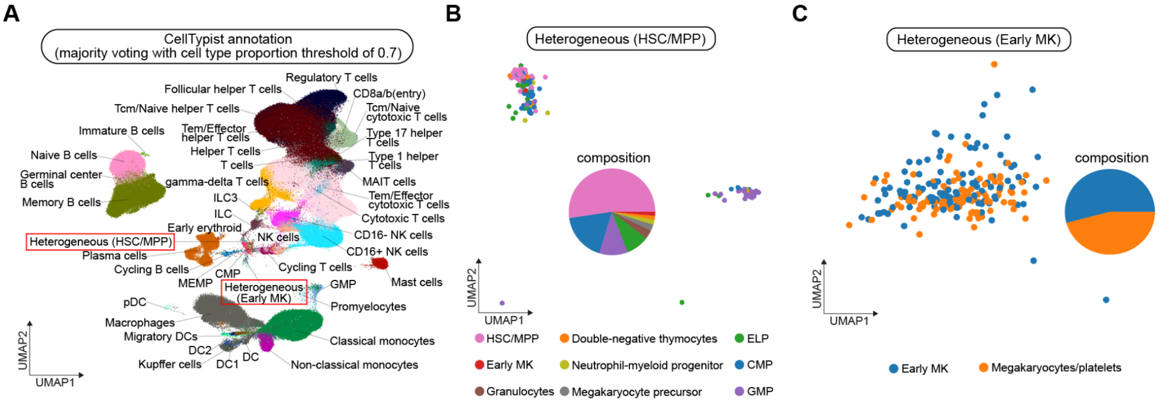

Fig. S26. Detection of heterogeneous cell clusters using CellTypist. (**A**) UMAP visualization of the immune cell compartment colored by cell types predicted using the CellTypist pipeline based on a proportion threshold of 0.7. Two heterogeneous clusters are marked, with the dominant cell types being ‘HSC/MPP’ and ‘Early MK’, respectively. (**B**) Zoomed-in view of the heterogeneous ‘HSC/MPP’ populations in two-dimensional UMAP representations. Middle pie chart shows the cell type compositions within this cell cluster. (**C**) As with (B), but for the heterogeneous ‘Early MK’ populations.

Table S1. Donor metadata.

Donor ID	Age Sex	Donor Type	Primary cause of death	Multi-trauma	Days in hospital	BMI	CMV EBV TOXO	Smoker	Alcohol (u/day)	Medication
A29	66-70 F	DCD	ICH	Y	2	30-35	CMV+ EBV+ TOXO-	N/K	<1	Desmopressin (Vaso)<br>Metaraminol (Vaso)
A31	51-55 M	DCD	ICH	Y	8	30-35	CMV+ EBV+ TOXO-	Y	<1	Noradrenaline (Vaso)<br>Co-amoxiclav (Ab)<br>Tazocin (Ab)
A35	61-65 M	DCD	ICH	N	2	20-25	CMV- EBV+ TOXO-	Y	>9	Noradrenaline (Vaso)<br>Desmopressin (Vaso)<br>Gentamicin (Ab)<br>Flucloxacillin (Ab)<br>Dexamethasone (Steroid)
A37	56-60 F	DCD	ICH	N	3	20-25	CMV- EBV+ TOXO-	Y	>9	Noradrenaline (Vaso)<br>Metaraminol (Vaso)<br>Co-amoxiclav (Ab)
A36	71-75 M	DCD	ICH	N	5	25-30	CMV- EBV+ TOXO +	Y	<2	Noradrenaline (Vaso)<br>Metaraminol (Vaso)<br>Amoxicillin (Ab) (pre-admission)<br>Flucloxacillin (Ab)<br>Gentamicin (Ab)<br>Clarithromycin (Ab)<br>Co-amoxiclav (Ab)<br>Prednisolone (Steroid) (pre-admission)
A52	61-65 M	DCD	ICH	N	2	35-40	CMV- EBV+ TOXO-	N/K	N/K	Noradrenaline (Vaso)<br>Metaraminol (Vaso)<br>Desmopressin (Vaso)<br>Co-amoxiclav (Ab)
582C	56-60 F	DCD	HBI due to CA	N	8	30-35	CMV- EBV+ TOXO-	N	<1	Noradrenaline (Vaso)<br>Co-amoxiclav (Ab)
621B	51-55 M	DBD	ICH	N	6	30-35	CMV- EBV+ TOXO-	N	N/K	Noradrenaline (Vaso)<br>Metaraminol (Vaso)<br>Vasopressin (Vaso)<br>Desmopressin (Vaso)<br>Gentamicin (Ab)<br>Flucloxacillin (Ab)
637C	51-55 M	DCD	ICH	N	2	25-30	CMV+ EBV+ TOXO-	Ex (>3yrs)	N/K	Noradrenaline (Vaso)<br>Vasopressin (Vaso)<br>Co-amoxiclav (Ab)<br>Methylpred (Steroid)
640C	71-75 F	DCD	ICH	Y	2	30-35	CMV+ EBV+ TOXO-	Ex (>40yrs)	>3	Noradrenaline (Vaso)<br>Co-amoxiclav (Ab)

D496	56-60<br>M	DBD	ICH	N	5	25-30	CMV+<br>EBV+<br>TOXO-	Y	<1	Norepinephrine (Vaso)<br>Vancomycin (Ab)<br>Piperacillin (Ab)<br>Tazobactam (Ab)<br>Solumedro (Steroid)
D503	66-70<br>F	DBD	HBI	N	5	25-30	CMV+<br>EBV+<br>TOXO-	N	<1	Norepinephrine (Vaso)<br>Vasopressin (Vaso)<br>Ab not known<br>Steroid not known

F = Female; M = Male; DCD = Donation after Circulatory Death; DBD = Donation after Brain Death; ICH = intracranial haemorrhage; HBI= Hypoxic Brain Injury; CA= Cardiac Arrest; BMI= Body Mass Index; CMV = Cytomegalovirus; EBV = Epstein-Barr virus; TOXO = Toxoplasmosis; Y = Yes; N = No; N/K=not known; Vaso = Vasoactive agents; Ab = Antibiotics within 2 weeks of death.

Table S2: Hashtags used in this study, supplied by BioLegend.

10X Genomics Chemistry	Hashtag	Catalog number	Barcode sequence
5'	TotalSeq-C0251	394661	GTCAACTCTTTAGCG
5'	TotalSeq-C0252	394663	TGATGGCCTATTGGG
5'	TotalSeq-C0253	394665	TTCCGCCTCTCTTTG
5'	TotalSeq-C0254	394667	AGTAAGTTCAGCGTA
5'	TotalSeq-C0255	394669	AAGTATCGTTTCGCA
5'	TotalSeq-C0256	394671	GGTTGCCAGATGTCA
5'	TotalSeq-C0257	394673	TGTCTTTCCTGCCAG
3'	TotalSeq-A0251	394601	GTCAACTCTTTAGCG
3'	TotalSeq-A0252	394603	TGATGGCCTATTGGG
3'	TotalSeq-A0253	394605	TTCCGCCTCTCTTTG
3'	TotalSeq-A0254	394607	AGTAAGTTCAGCGTA
3'	TotalSeq-A0255	394609	AAGTATCGTTTCGCA
3'	TotalSeq-A0256	394611	GGTTGCCAGATGTCA
3'	TotalSeq-A0257	394613	TGTCTTTCCTGCCAG

Table S3. Sequencing metrics from each sample in this study

sample	average_n_genes	average_umi_counts	qc-pass_cell_count
BLD_621B	1,704	2,429	108
BLD_637C	1,867	2,746	784
BLD_A35	1,766	2,443	1,413
BLD_A36	2,008	2,586	3,372
BLD_D496	1,939	2,528	9,192
BLD_D503	1,661	2,291	12,827
BMA_621B	1,792	2,186	1,186
BMA_637C	2,047	2,592	2,050
BMA_640C	2,302	2,684	2,259
BMA_A29	1,651	2,197	1,870
BMA_A31	1,888	2,334	433
BMA_A35	2,174	2,667	1,515
BMA_A36	1,874	2,342	1,685
BMA_A37	1,705	2,149	1,083
BMA_D496	1,896	2,591	13,187
BMA_D503	2,166	2,660	17,659
CAE_A29	2,305	2,037	885

CAE_ A31	1,526	1,733	818
CAE_ A35	1,417	1,846	375
CAE_ A36	1,152	1,891	137
DUO_ A31	1,461	2,100	1,141
DUO_ A35	1,371	1,790	41
DUO_ A37	1,507	2,368	29
ILE_A 29	1,754	1,982	4,494
ILE_A 31	2,200	2,189	80
ILE_A 35	1,384	1,918	317
ILE_A 36	2,404	2,369	337
ILE_A 37	1,686	2,236	1,208
JEJEPI _582C	1,211	2,058	677
JEJEPI _637C	1,381	2,075	7,932
JEJEPI _640C	1,904	2,662	9,218
JEJEPI _D496	1,746	2,599	7,224
JEJEPI _D503	1,722	2,486	10,043
JEJLP_ 582C	1,483	2,190	449
JEJLP_ 637C	1,576	2,401	65
JEJLP_ 640C	1,770	2,422	7,960

JEJLP_D496	2,152	2,380	8,048
JEJLP_D503	1,859	2,438	10,764
LIV_637C	1,941	2,800	2,888
LIV_640C	1,998	2,752	3,885
LIV_A29	1,423	2,202	706
LIV_A31	1,385	2,184	2,423
LIV_A35	2,167	2,772	252
LIV_A36	792	1,751	1,898
LIV_A52	1,958	2,721	1,818
LLN_D496	1,823	2,358	16,138
LLN_D503	1,715	2,338	8,504
LNG_637C	2,150	2,872	82
LNG_640C	2,550	2,648	206
LNG_A29	1,851	2,480	3,301
LNG_A31	1,778	2,394	1,802
LNG_A35	1,393	2,248	51
LNG_A36	3,237	2,796	2,618
LNG_A37	1,510	2,269	180
LNG_A52	1,847	2,516	696

LNG_ D496	3,657	2,927	18,513
LNG_ D503	2,151	2,591	9,724
MLN_ 582C	1,694	2,496	441
MLN_ 621B	1,410	2,299	2,158
MLN_ 637C	1,648	2,543	6,451
MLN_ 640C	1,821	2,624	5,374
MLN_ A29	1,181	2,079	2,838
MLN_ A31	1,686	2,388	2,374
MLN_ A35	1,633	2,390	1,920
MLN_ A36	1,844	2,481	1,450
MLN_ A37	1,504	2,218	343
OME_ A31	1,763	2,342	79
OME_ A35	2,087	2,566	216
OME_ A36	2,496	2,875	262
OME_ A37	1,841	2,417	347
SCL_A 29	1,658	1,973	1,129
SKM_ A29	1,347	2,275	22
SKM_ A35	2,046	2,666	809
SKM_ A37	1,473	2,303	21

SPL_5 82C	1,387	2,279	6,536
SPL_6 21B	1,808	2,492	4,590
SPL_6 37C	1,815	2,634	5,347
SPL_6 40C	2,017	2,636	4,761
SPL_A 29	2,074	2,576	3,627
SPL_A 31	1,669	2,278	2,175
SPL_A 35	2,064	2,547	2,551
SPL_A 36	1,866	2,413	8,479
SPL_A 37	1,507	2,155	3,411
SPL_A 52	1,492	2,285	1,610
SPL_D 496	2,082	2,615	16,987
SPL_D 503	1,901	2,483	12,034
TCL_ A29	1,774	1,964	1,829
THY_ A31	1,556	2,408	354
TLN_5 82C	1,321	2,190	3,770
TLN_6 21B	1,563	2,331	3,096
TLN_6 37C	1,759	2,499	6,899
TLN_6 40C	2,045	2,680	4,787
TLN_ A29	1,614	2,282	4,572

TLN_ A31	1,165	2,086	3,005
TLN_ A35	1,742	2,443	2,450
TLN_ A36	1,705	2,383	4,979
TLN_ A37	1,742	2,301	4,340
TLN_ A52	1,010	2,089	232

75. D. J. Carpenter, T. Granot, N. Matsuoka, T. Senda, B. V. Kumar, J. J. C. Thome, C. L. Gordon, M. Miron, J. Weiner, T. Connors, H. Lerner, A. Friedman, T. Kato, A. D. Griesemer, D. L. Farber, Human immunology studies using organ donors: Impact of clinical variations on immune parameters in tissues and circulation. *Am. J. Transplant*. **18**, 74–88 (2018).

76. E. P. Mimitou, A. Cheng, A. Montalbano, S. Hao, M. Stoeckius, M. Legut, T. Roush, A. Herrera, E. Papalexi, Z. Ouyang, R. Satija, N. E. Sanjana, S. B. Koralov, P. Smibert, Multiplexed detection of proteins, transcriptomes, clonotypes and CRISPR perturbations in single cells. *Nat. Methods*. **16**, 409–412 (2019).

77. B. Pijuan-Sala, J. A. Griffiths, C. Guibentif, T. W. Hiscock, W. Jawaid, F. J. Calero-Nieto, C. Mulas, X. Ibarra-Soria, R. C. V. Tyser, D. L. L. Ho, W. Reik, S. Srinivas, B. D. Simons, J. Nichols, J. C. Marioni, B. Göttgens, A single-cell molecular map of mouse gastrulation and early organogenesis. *Nature*. **566**, 490–495 (2019).

78. H. W. King, N. Orban, J. C. Riches, A. J. Clear, G. Warnes, S. A. Teichmann, L. K. James, Single-cell analysis of human B cell maturation predicts how antibody class switching shapes selection dynamics. *Sci Immunol*. **6** (2021), doi:10.1126/sciimmunol.abe6291.

79. J. A. V. Heiden, J. A. Vander Heiden, G. Yaari, M. Uduman, J. N. H. Stern, K. C. O'Connor, D. A. Hafler, F. Vigneault, S. H. Kleinstein, pRESTO: a toolkit for processing high-throughput sequencing raw reads of lymphocyte receptor repertoires. *Bioinformatics*. **30** (2014), pp. 1930–1932.

80. J. Ye, N. Ma, T. L. Madden, J. M. Ostell, IgBLAST: an immunoglobulin variable domain sequence analysis tool. *Nucleic Acids Res.* **41**, W34–40 (2013).

81. D. Gadala-Maria, G. Yaari, M. Uduman, S. H. Kleinstein, Automated analysis of high-throughput B-cell sequencing data reveals a high frequency of novel immunoglobulin V gene segment alleles. *Proc. Natl. Acad. Sci. U. S. A.* **112**, E862–70 (2015).

82. N. T. Gupta, J. A. Vander Heiden, M. Uduman, D. Gadala-Maria, G. Yaari, S. H. Kleinstein, Change-O: a toolkit for analyzing large-scale B cell immunoglobulin repertoire sequencing data. *Bioinformatics*. **31**, 3356–3358 (2015).

83. V. Svensson, E. da Veiga Beltrame, L. Pachter, A curated database reveals trends in single-cell transcriptomics. *Database* . **2020** (2020),

doi:10.1093/database/baaa073.

84. A. Haque, J. Engel, S. A. Teichmann, T. Lönnberg, A practical guide to single-cell RNA-sequencing for biomedical research and clinical applications. *Genome Med.* **9**, 75 (2017).

85. T. Abdelaal, L. Michielsen, D. Cats, D. Hoogduin, H. Mei, M. J. T. Reinders, A. Mahfouz, A comparison of automatic cell identification methods for single-cell RNA sequencing data. *Genome Biol.* **20**, 194 (2019).

86. M. Lotfollahi, M. Naghipourfar, M. D. Luecken, M. Khajavi, M. Büttner, M. Wagenstetter, Ž. Avsec, A. Gayoso, N. Yosef, M. Interlandi, S. Rybakov, A. V. Misharin, F. J. Theis, Mapping single-cell data to reference atlases by transfer learning. *Nat. Biotechnol.* (2021), doi:10.1038/s41587-021-01001-7.

87. N. D. Köhler, M. Büttner, N. Andriamanga, F. J. Theis, Deep learning does not outperform classical machine learning for cell-type annotation. *bioRxiv* (2021), p. 653907.

88. G. La Manno, D. Gyllborg, S. Codeluppi, K. Nishimura, C. Salto, A. Zeisel, L. E. Borm, S. R. W. Stott, E. M. Toledo, J. C. Villaescusa, P. Lönnerberg, J. Ryge, R. A. Barker, E. Arenas, S. Linnarsson, Molecular Diversity of Midbrain Development in Mouse, Human, and Stem Cells. *Cell*. **167**, 566–580.e19 (2016).

89. R. Elmentaite, A. D. B. Ross, K. Roberts, K. R. James, D. Ortmann, T. Gomes, K. Nayak, L. Tuck, S. Pritchard, O. A. Bayraktar, R. Heuschkel, L. Vallier, S. A. Teichmann, M. Zilbauer, Single-Cell Sequencing of Developing Human Gut Reveals Transcriptional Links to Childhood Crohn's Disease. *Dev. Cell*. **55**, 771–783.e5 (2020).

90. F. Pedregosa, G. Varoquaux, A. Gramfort, V. Michel, B. Thirion, O. Grisel, M. Blondel, P. Prettenhofer, R. Weiss, V. Dubourg, Others, Scikit-learn: Machine learning in Python. the Journal of machine Learning research 12 (2011).

91. K. J. Travaglini, A. N. Nabhan, L. Penland, R. Sinha, A. Gillich, R. V. Sit, S. Chang, S. D. Conley, Y. Mori, J. Seita, G. J. Berry, J. B. Shrager, R. J. Metzger, C. S. Kuo, N. Neff, I. L. Weissman, S. R. Quake, M. A. Krasnow, A molecular cell atlas of the human lung from single-cell RNA sequencing. *Nature*. **587**, 619–625 (2020).

92. J. Cao, D. R. O'Day, H. A. Pliner, P. D. Kingsley, M. Deng, R. M. Daza, M. A. Zager, K. A. Aldinger, R. Blecher-Gonen, F. Zhang, M. Spielmann, J. Palis, D. Doherty, F. J. Steemers, I. A. Glass, C. Trapnell, J. Shendure, A human cell atlas of fetal gene expression. *Science*. **370** (2020), doi:10.1126/science.aba7721.

93. Y. Hao, S. Hao, E. Andersen-Nissen, W. M. Mauck 3rd, S. Zheng, A. Butler, M. J.

Lee, A. J. Wilk, C. Darby, M. Zager, P. Hoffman, M. Stoeckius, E. Papalexi, E. P. Mimitou, J. Jain, A. Srivastava, T. Stuart, L. M. Fleming, B. Yeung, A. J. Rogers, J. M. McElrath, C. A. Blish, R. Gottardo, P. Smibert, R. Satija, Integrated analysis of multimodal single-cell data. *Cell*. **184**, 3573–3587.e29 (2021).

94. J. C. Kimmel, D. R. Kelley, Semisupervised adversarial neural networks for single-cell classification. *Genome Res.* **31**, 1781–1793 (2021).
